# Supplementary material for: Exploring what is important during burn recovery: a qualitative study investigating priorities of patients and healthcare professionals over time
Source: BMJ Open. 2023 Feb 10;13(2):e059528. doi: 10.1136/bmjopen-2021-059528 (PMC9923305; doi:10.1136/bmjopen-2021-059528)
Supplement: Supplementary data [file bmjopen-2021-059528supp001.pdf]

Supplemental File - Table S1: Exemplary interview topic guide for patient participants

Topic	Questions
**Intro**	• Introduction to researcher, topic, aims and objectives of the study.<br>• Ask if any questions<br>⇒ *Check that participant is happy to audio record the interview and check consent*
**Background/ warm-up**	• First of all, could you tell me a bit about you?<br>o *Prompt: do you work, do you have children etc*<br>o *Prompt for children: how old are you?*<br>• Could you tell me about how you/your child came to have the burn?
**Short-term: Immediately after injury**	• Thinking about the time when you were burned, what were your worries about the immediate effects of the burn at that time?<br>o *Prompt: e.g. survival, pain*<br>o *Anything else you can think of? Any other concerns you may have had?*<br>• Still thinking about the time when you were burned, what were your concerns about future effects?<br>o *Prompt: scarring, movement*<br>*Areas of your life that it was most important to ensure the burn didn't affect eg function (eg related to work, school), the look of the burn (confidence, work, school, friends)? Anything else?*<br>• **What would you say were the most important aspects during that time?**
**Medium-term: After discharge**	• Thinking about the time when you started to recover/got home/were discharged, what were your concerns in relation to your recovery?<br>o *Was your daily life affected? If so, in what ways? Return to normal.*<br>▪ *Prompts could include work life; social life; family life; relationships (including intimacy)*<br>o *Were there any issues that affected you that the doctors didn't ask about at your out-patient appointments?*<br>o *Were there issues that bothered you more than they seemed to bother the medical staff?*<br>o *Were there issues that the doctors asked about that didn't worry you?*<br>• During the recovery (at home), what were your concerns specifically about the future?<br>o Prompt: QoL, physical,<br>• Were your family and friends (and if applicable, partner) worried about any effects of your burn at any time after the burn injury? If so, what were they worried about?<br>o *Prompt: (as above; work life; social life; family life; relationships (including intimate relationships)*<br>o *Anything else? What other concerns were there?*<br>• **What, in your view, was most important to you during the time after discharge?**
**(Current) Recovery & Future**	• What matters to you (now) while you are getting better?<br>o *Prompts: similar to above and laddering questions*<br>• And thinking about the future, what are you particularly worried about?<br>• **And again, thinking about your situation now, what is most important to you at the moment in relation to your recovery?**<br>o Do you think this will matter further down the line?<br>o If there are any areas of healthcare that could have been improved, what would that/they be?<br>o *Prompt: treatment of infection, management of pain, improving success of skin-grafting?*
**Wrap-up questions**	• To finish with, could we summarise what the most important areas of your recovery after the injury were to you?<br>⇒ *Wrap up interview*
