# Supplementary material for: Exploring what is important during burn recovery: a qualitative study investigating priorities of patients and healthcare professionals over time
Source: BMJ Open. 2023 Feb 10;13(2):e059528. doi: 10.1136/bmjopen-2021-059528 (PMC9923305; doi:10.1136/bmjopen-2021-059528)
Supplement: Supplementary data [file bmjopen-2021-059528supp002.pdf]

Supplemental File - Table S2: Exemplary interview topic guide for professional participants

Topic	Questions
**Intro**	• Introduction to researcher, topic, aims and objectives of the study.<br>• Ask if any questions<br>⇒ *Check that participant is happy to audio record the interview and check consent*
**Background/ warm-up**	• First of all, could you tell me a bit about you and your role as a healthcare professional?<br>o *Prompt: how long have you been at the hospital for, what are your responsibilities?*<br>o *What is your involvement with burns injuries? How long working in burns?*<br>• What are your experiences been with burns patients?<br>• *Which part of the recovery journey are you involved in? When/how regular do you see them?*
**Short-term: Immediately after injury**	• Going back to the very beginning, when patients are admitted to the hospital, what are the relevant outcomes from a clinical perspective?<br>o E.g. survival, scaring, pain?<br>• **What is the most important outcome?**<br>• What do you think patients are worried about the most during that time?<br>• **What do you think is the most important outcome for patients at that time?**
**Medium-term: After discharge**	• Moving on to when the patient gets discharged for recovery at home, what are relevant outcomes for you from a clinical viewpoint?<br>• **And what was the most important one taking into consideration the recent discharge?**<br>• And again, what do you think the patients are concerned about when they get discharged from hospital?<br>o *Prompt: daily life, QoL, work, social,*<br>• **What do you think is most important to the patients at the time they are being discharged?**<br>• How do you think do the patients views differ from their families and friends? What are they worries about?<br>• *Prompt: (as above; work life; social life; family life; relationships (including intimate relationships)*
**(Current) Recovery & Future**	• Thinking about burns injury outcomes further down the line, what is important for you in the long run? What is most important?<br>o What do you think is the most important to patients when thinking about the really long-term effects in the future?
**Wrap-up questions**	• To finish with, could we summarise what the most important areas of patient recovery after the injury?<br>⇒ *Thanks to interviewee*
