# Supplementary material for: Exploring what is important during burn recovery: a qualitative study investigating priorities of patients and healthcare professionals over time
Source: BMJ Open. 2023 Feb 10;13(2):e059528. doi: 10.1136/bmjopen-2021-059528 (PMC9923305; doi:10.1136/bmjopen-2021-059528)
Supplement: Supplementary data [file bmjopen-2021-059528supp003.pdf]

Supplemental File – Table S3: Outcome classification for burn care research (adapted from Young et al. 2019)

Outcome category	Outcome domain
**Patient-reported**	Ability to carry out daily tasks
	Anxiety about medical procedures and appointments
	Generalised anxiety
	Appearance
	Blister fluid
	Burden of care
	Comfort of dressings
	Psychological well-being
	Mental ability
	Quality and quantity of sleep
	Effect of scar on movement (contractures)
	Return to work/school or previous function
	Burn wound pain
	blister fluid
	Pain during procedures
	Scar pain
	Itch
**Pathophysiological**	Ability to fight infection
	Body weight maintenance
	Bone strength
	Breathing and lungs
	Donor site problems after healing
	Effect of burn on genes
	Effect of burn on how the body uses energy
	Effect on heart and blood circulation
	Fitness
	Growth in children
	How well muscles work
	Mobility
	Kidney function
	Liver function
	Medical tests to indicate how unwell a patient is
	More than one organ failing (multiorgan failure)
	Muscle strength
	Stomach and bowel function
	Burn wound healing
	Donor site healing
**Complications**	Complications of drug treatment
	Blood product transfusion
	Burn wound infection
	Death from burn injury
	Death from any cause
	Effects of fluid from a drip
	Infections other than burn wound infection

	Sepsis
**Scar-related**	Scar colour
	Scar texture
	Scar size
	Treatment for scars
**Healthcare-related**	Costs of treatment for NHS/hospital
	Length of hospital stay
	Length of stay in intensive care unit
	Length of time on life support machine
	Use of medicines to treat symptoms

Young AE, Davies A, Bland S, Brookes S, Blazeby JM. Systematic review of clinical outcome reporting in randomised controlled trials of burn care. BMJ open. 2019 Feb 1;9(2):e025135.
