# Supplementary material for: Exploring what is important during burn recovery: a qualitative study investigating priorities of patients and healthcare professionals over time
Source: BMJ Open. 2023 Feb 10;13(2):e059528. doi: 10.1136/bmjopen-2021-059528 (PMC9923305; doi:10.1136/bmjopen-2021-059528)
Supplement: Supplementary data [file bmjopen-2021-059528supp004.pdf]

Supplemental File – Descriptive reports

1. Interviews with patients

Scarring

Worries about future scarring were apparent for some participants and their families in the early days following the injury.

> *Well the worries were the scarring and whether we'd need to have- at that point in time we didn't know- no-one could tell us how bad it was. Before we got to (city) burns no-one could tell us how bad it was, so our worry was whether she needed to have a skin graft or was she going to have permanent scars because it was so horrible on her face-[BCH 01, parent]*

> *Just wondering what the scarring would do, whether the skin would be very tight. Because you do see people who've had burns, like facial burns and sometimes their skin is very pink and red and then you do notice it. But yes, I think that was about it really. It's just how functional your hands would be when they're recovered.[NBT 27]*

The majority of participants interviewed went on to develop some scarring as their wound healed. Many of these participants described the physical appearance of their scars, talking about the colour (*'really pink' [BCH FW] 'quite red, it's not a natural skin colour [NBT 005])* and the shape (*'quite gnarly' [NBT 02], 'pretty bumpy' [BCH 19]*). One participant talked about how the appearance of their scar was getting worse during the healing process.

> *you look progressively worse as your function gets progressively better. So I had a- I struggled with the fact that I was physically feeling up to it, going out, doing things and I was looking more scarred, more bumpy because scar tissue takes a while to form and, you know, you just end up, as the peeling process happens, looking worse as time goes on until a point where then you'll stop and it will start to improve but you need to do a lot to improve the appearance so for the last three to four months I've been looking progressively worse.[C&W 004]*

Several participants talked about how their scars negatively affected their feelings about their appearance.

> *I felt I looked like Frankenstein [CW 002]*

> *How it looked was a big- I wouldn't be able to go out on a beach and just feel confident in a bikini or something [NBT 002]*

> *I worry this will be it, it's not going to get any better but I've said that every month since I was discharged and there's always been changes. But I cry… every time I go in I cry going is this it? I can't look like this for the rest of my life. [NBT 013]*

The reactions of others to their scars also had an impact. Several participants described being stared at by others. For some, the reactions of others were a strong determinant of how they themselves felt about their scars.

> *some guy on the bus, this was a grown man in his thirties, I was on the bus on my way to hospital once and I was standing 'cos there were no seats, he was looking at me and doing wretch noises as though he was vomiting, looking at me and doing-*

if other people were like accepting of it I would be more accepting of it, I wouldn't be as embarrassed but you get these people that are just really naïve. [C&W 002]

It's more a question of having an overall appearance that causes people to look at you in the street which is uncomfortable. If you had scars that were maybe subtle, not a big deal, some people might notice when they're close up but I think, you know, as the healing process happens with burns you go very red, very bumpy, it's really obvious and I think anything obvious in terms of the reaction you see from other people affects you more than how you see yourself. [C&W 004]

However, some participants said that they were not concerned about their scars.

Personally doesn't bother me, doesn't bother me at all. I can see why it might bother some people but personally no. [NBT 08]

Yeah, don't get me wrong, its not nice to have a scar like that but its not the end of the world, I'm not too vain that I can't still put shorts on sort of thing, but yeah, it is what it is. [NBT 12]

Older participants tended to be less affected by their scarring. The location of the scar was also important – participants with scarring in less visible places tended to be less bothered by it.

It could have been on my face which the ward that I was in at the time, there was a lady opposite me in the ward that had had full body burns from her Christmas lights going up and I looked at her and I thought ok, you need to be happy about this, it could have… its an impact but its… I'm left with a scar on my leg that is not the end of the world. [NBT 12]

So it's quite visible, it's from my chin, it's very thick on my neck, you can't miss it. I get funny looks from children and so yeah, and just… I feel like I'm being remembered for the girl with the burns and stuff like that. [NBT 13]

Pain

In the immediate aftermath of the burn, pain was an important concern, often the most important concern, for the majority of patients.

'Yeah. Yeah I don't think I would have thought of anything else. Yeah, just concerned about the pain.'

'At first its just, when it first happens its just about the pain. That's it. You can't really… because it wasn't… it was only that area, I was still completely compos mentis and I was in pain but really that is just all you're focussing on at the time, its just trying to mentally manage the pain and…'

Pain was also a concern for other family members

'It is just the realisation of sort of thinking [inaudible] when I realised how much pain he was in, how painful it was to clean it, how painful it was to dress it and you he is only tiny.' [parent]

However, some participants reported that pain was not an early concern.

'Yeah, my adrenalin was going so much that I didn't notice the pain actually.'

'There was no pain really. It's really strange, yeah. I think because I think the wound got quite deep with the acid burn it sort of killed away part of the nerves-'

Pain experienced by participants occurred not just as a result of the burn but also due to treatments received such as cleaning the wound, dressing changes, surgeries and skin grafts.

Adequate pain management was identified as important both in the short term and after discharge from hospital.

> *‘Yeah, they kept me very well medicated. I don’t remember being in terrible pain. I remember when they would ask me every day what my pain scale (inaudible) one to whatever and I’m sure I always answered quite low because they kept it so well’.*

> *‘they literally just on the Saturday morning came up and said consultant will be coming round, he might be able to sign you off. He didn’t come round, the nurse came up and said ok you’re good to go and just pushed me out the door with no medication, no occupational therapist, no guidance as to what expect from the wound, no knowledge as to what to expect with pain, no pain management tablets, no nothing, they literally just pushed me out the door.’*

For some participants, adequate pain management needed to be balanced against its potential side effects

> *‘But for me I preferred to cope with that for five minutes [of wound being washed down] than take a lot of painkillers which can give you other problems. ‘*

For participants who experienced deeper burns, pain increased in the medium term due to nerve regeneration

> *‘I thought that it doesn’t bother me because I’ve got on a previous burn, um, I couldn’t feel anything to start off with either but gradually I’m getting little shooting pains and I spoke to one of the doctors and he said it’s because all the nerves are finally like reattaching and then it’s a bit confused as to where to kind of attach to so that’s where the little itchy kind of sharp pain is coming from so I’m hoping, ‘cos this is only two months old-‘ [NBT009]*

Although pain was less commonly reported as an issue in the long-term, it was clear that some participants continued to experience pain or sensitivity in the affected area and this was accepted as a part of life.

> *‘because it's just sensitive, it's the sensitivity of it. Yeah, it's very sensitive on the little finger, my thumb and the palm of my hand. But I think as she said, give that another year and it will get better. But it's fine. ‘ [NBT027]*

> *‘Um, I don’t know if its psychological but there’s always a bit of pain, I don’t know but you get used to it but I was worried at first could I do any movement or not, you know, because I didn’t want to damage- ‘cos they’ve done such good work as well.’ [NBT007]*

> *‘So biggest problem is sometimes, you know, a bit of pain in the leg for example, but it doesn’t stop me doing things, you know, like walking but sometimes like this morning I couldn’t (inaudible) and its more painful than- it is perhaps because its tighter than it should be, I don’t know, but it was more painful but its easing.’ [NBT007]*

Itching

Itching was a commonly reported problem amongst participants. For most, itching was not experienced immediately after the injury but commenced as the wound began to heal.

> *Oh itching was terrible! (laughs) And not being able to actually scratch it, so the only way I could do that was to move my foot around and the dressing would sort of work its way off (laughs) so, yeah I did get a lot of itching.[NBT 014]*

> *sometimes I think the itch was worse than the pain [C&W 06]*

Not initially. Probably after, ooh I reckon a month. Obviously when it started to heal I think, or that's what I assumed anyway. [NBT 014]

In addition to being unpleasant, some participants reported that itching interfered with daily living. Itching was described as being worse at night which affected sleep. Two participants described their anxiety about itching in public.

Yes. Yes. I mean it itches much less than it was but when I get hot, particularly at night that's when it's such- it's worse [NBT 011]

Itchiness. I feel like that was one of the most unbearable things ever and the constant anxiety that I'm going to start itching randomly in public and I can't control it [C&W 02]

Itching continued to be a problem for a long time for some participants, although most reported that it did ease in the long term.

would say, nearly five years in now, but the itch has gone. I can remember talking to another patient that I met that had a burn injury previous to mine and they said that it took quite a few years and it did, I think it took about four years to actually- [C&W 06]

Occasionally it itches. Very occasionally. Not very often though, no, but I still get that fizzy feeling sometimes, very rarely now but occasionally [NBT 014]

Tightness

Several participants talked about tightness in the area of their burns as they healed. For some participants this tightness was a source of discomfort and distress.

when I think back to it now my burns were actually rigid, they would not move. They were literally as if they were set in stone- [C&W 002]

I think there was- the skin was quite taut so therefore uncomfortable. [NBT011]

so once I started getting the movement back I was just scared, I literally thought that all my skin was going to just rip in half. It's quite a scary experience and I'm not- I don't think I'm alone on that, she said quite a lot of people feel that way. [NBT 013]

Tightness restricted participants ability to move and impacted on their activities. It was also a source of pain.

like if I go in the garden and do some weeding because it's (inaudible)- So to then down ways, I used to bend down ways with the legs straight but they say no you have to bend down like you would so it's trying to build down- now to protect your-[NBT 007]

So biggest problem is sometimes, you know, a bit of pain in the leg for example, but it doesn't stop me doing things, you know, like walking but sometimes like this morning I couldn't (inaudible) and its more painful than- it is perhaps because its tighter than it should be, I don't know, but it was more painful but its easing. [NBT 007]

Several participants described having treatments to help with the tightness which reduced over time. However, some participants reported that they continued to experience tightness in the longer term.

Infection

Several participants reported experiencing one or more infections in the period following their injury. Participants described the appearance of the infected area, smell and general feelings of being unwell. The appearance and smell were also a source of distress for some patients and family members.

> *'and then on the afternoon I looked and I could see all my wrist and hand getting quite puffy and red and swollen and I thought oh I don't like the look of that' [NBT 009]*

> *'cos the infection just made me feel really ill as well.' [NBT 002]*

> *'I'm sure dad won't mind me saying, when the infection was there the smell was horrendous,' [NBT 010]*

Infections sometimes led to a lengthened hospital stay or readmission to hospital. Participants were also concerned about the possibility that infection could spread from the burn site.

> *'But I think I was most worried about the infection, when they said that it was infected and obviously it could be in his bloodstream, I think that scared me the most.' [BCH 019]*

> *'Well when the nurse mentioned sepsis and things like that you start to panic.'[NBT014]*

Worries about developing an infection were a common theme for many participants in the early period following their injury, although there was some variability in this.

> *'Yeah, so pain it was ok and you could see it was slowly getting better and I was really panicked about infections. Infections really bothered me but once the sort of the weeping stopped it was ok, you could see it was healing so that went.' [BCH 018]*

> *'I worried an awful lot about that. I did worry an awful lot about infection.' [C&W 006]*

These worries were greatest in those who had experienced a prior infection.

> *'And then I think it was probably about a month later it looked- I wondered if it was going to be infected again so I went off to the A&E and they reassured me it wasn't.' [NBT014]*

Participants were also worried about keeping the injury clean in order to prevent infection

> *'I think it was more that he has trouble staying clean, you know, he's very hands on and, you know, likes playing out in the garden and when we went swimming and things like that, I think that was-' [BCH 03]*

Worries about developing an infection were not a concern in the long-term.

Appearance of the wound

Several participants talked about the appearance of their wound in the days following their injury. Participants described the blistering, the colour and the smell of their wound. Wound appearance was a source of distress for some patients and their significant others.

> *I was pretty horrified when I saw it. I don't know whether it was just 'cos it was just raw flesh. It was just raw. It was disgusting. [C&W 005]*

> *I think initially it was the look of it because that was pretty scary, that was pretty frightening.[BCH 020]*

The effect of the wound on their appearance was also a source of distress.

> *she didn't want it [the dressing] taken off because it hurt her and then when she saw her face she just broke down. She said I'm really ugly mummy.[BCH 001]*

> *I was just getting too upset and depressed over the fact that I had burns and I looked so different [C&W 002]*

The appearance of the wound informed participants views on the seriousness of their injury.

> *But when it comes to over 24 hours or whatever it started to look way worse, and I was much more worried about the seriousness of it. [BCH 017]*

One participant remarked that they did not realise the seriousness when the wound was dressed preventing them from being able to see it.

> *I had no idea how bad it was. I was very bandaged up so no. [NBT 013]*

For many participants, the appearance of the wound was linked to their feelings about the time it was taking to heal.

> *like when its fresh you're like oh god, this is never going to heal but yeah.[NBT 002]*

> *I don't know because although its healed lovely, it's still loitering because I can still see- I mean where the skin's purple and sometimes I get like a white raised area in the middle and I think oh what's that? And when I (inaudible) the cream it's gone. It's almost like it's sort of hang on a minute, I'm taking my time, you know. [NBT 014]*

Time to healing

Some participants expressed feelings of surprise, frustration or worries about the amount of time it was taking for the wound to heal. Many seemed to feel that the healing took longer than they had expected.

> *I don't worry about the scar side of it, it was just the… the worry side of it was actually just the time it was taking. [NBT 012]*

> *I was just saying to (husband) the other day, he was like I can't believe how long it's all taking 'cos it happened, I don't know when it happened, like beginning of August and now we're in October and I feel like we're still kind of still doing it. [BCH F.W.]*

> *At the time it took ages and it was a drag coming up here every other day. [NBT 019]*

Uncertainty, lack of knowledge and the need for information

A lot of participants described feelings of uncertainty following their injury. These uncertainties related to the how the injury happened, the treatments needed and their possible outcomes as well as the likely course of their recovery.

> *And I suppose as well, because during the time that we were kind of up in the air as to whether he would have the operation or not, we saw loads of different people on different rotas and stuff, and I kind of felt like there were lots of different opinions flying around about how well it was healing, how well it wasn't healing, what the next course of treatment would be. And although I understand why that was the case, because in the main they were kind of waiting to see what the wound was going to do, we felt very up in the air about how… what was going to happen, and whether what happened eventually would have actually been the right thing. [BCH 0017]*

Yeah, you've got no information, no knowledge and the pain materialises, you don't know whether that's supposed to happen, was that not supposed to happen, is something going wrong. [NBT 12]

A clear need for information to lessen feelings of uncertainty was apparent. Many participants spoke positively about staff and services who met their information needs and explained things in a non-technical manner. Some participants felt that they did not get enough information and might have coped better had they had done so. For participants with more severe burns requiring a hospital stay, adequate information at the point of discharge home was important as was the availability of a point of contact for any future questions.

Very good. (A) was particularly good because he spent so much time with me 'cos he was in and out and he explained everything so thoroughly in a way that you could understand yourself, if you know what I mean, rather than having to look words up. (laughs) It's when they talk in jargon, if you know what I mean.[NBT 014]

one bit of advice that I would give throughout this is to give the patient clarity as to what's happening and the expectations where I wasn't given enough information and knowledge of what's happening now, what's going to happen, what could happen, what might not happen, I was signed off from the hospital and they hadn't said to me that the nerve endings will come back and then you'll start to suffer pain. So when I was signed off I didn't have really very much pain within 36 hours, it nearly broke me where the- going from there down to stood up, the rush of blood to the burn, I didn't know to expect it so it was quite a shock to the system. So I would just say just information, information, information. [NBT 012]

Although increased knowledge was generally seen as a positive thing, some patients mentioned that there were downsides to having too much information.

me and my family are very inquisitive people that need, you know, to know every possible scenario so there were a couple of times when we thought I'd have to have operations on things that I didn't need to have an operation on in the end so I think on the one hand it's great to be told of the things that are potentially going to go wrong or are going wrong and on the other it gave us maybe unnecessary cause for concern about procedures I ended up not needing. [C&W 004]

Several patients acknowledged that some aspects of their treatment and recovery were simply uncertain and that it would never be possible to have complete information.

I'm just obviously concerned about the future and things that no-one can tell me like in two years' time will he still need to wear the sleeve or is he still going to have to be creaming but in regards to everything that I need to know now, I feel that they've been brilliant, they've explained everything to me and to (child). [BCH 019]

Although themes of uncertainty and the need for knowledge and information were most commonly described by patients during the earlier stages of their recovery, some participants who had been left with more serious scarring described long-term term uncertainties and informational needs, particularly in relation to scar management treatments.

the problem I'm having at the moment is that there's no (sighs) there's still so few guarantees, or there are none so it's always so difficult 'cos you don't want to be making something worse if what you care about is the aesthetic. [CW 001]

And then I think in terms of, this is a big ask really but I think in terms of kind of follow-up, if there are new things, you know, I took myself off to my GP and said can you re-refer me back into the system

'cos I want to see if there are new things but if there was ever an opportunity to be a forum or something to be kept abreast of new developments-[CW 001]

Psychological effects

Several participants reported feelings of shock or fear in the immediate aftermath of their injury. These feelings arose as a result of the circumstances in which the injury occurred or due to the extent of the injuries received.

The first part, on the day, it was just, yeah, the whole scorch thing, the instant impact of it all going off, the shock factor.[NBT 12]

Amongst participants whose injury required a hospital stay, several participants commented on the value of psychological support being available.

just to know someone was there actually so it didn't mean that I had to deal with it on my own. I didn't make full use of it, I didn't feel the need but it was there. [C&W 004]

In the weeks following the injury, some participants reporting difficulty sleeping or of having nightmares or flashbacks.

Yeah. I do remember having quite a lot of hallucinations and I now know they're not real but at the time, for the first couple of weeks when I was up on the burns ward I didn't sleep unless they knocked me out because I lived in fear that someone was going to come and get me and put me on fire again.[NBT 0013]

Some participants also experienced guilt as they blamed themselves for the injury.

I suppose because I was so upset, and because I felt completely responsible for what had happened, I was predicting that, and I was quite vocal about that to a few people. Not many people, you know, two or three people. So that in turn made them more worried about how much kind of guilt I was taking on and I [inaudible] responsible I felt for what happened. [BCH 017]

In some cases, participants' experiences of receiving treatments also had some psychological consequences. This was more common when the person who had the burn was a child. Parents described how their child was now fearful of situations that reminded them of unpleasant or painful treatments (e.g. bathing) or of their experiences of being in hospital.

I don't know if it's got anything to do with it but like he mentally changed when he was in hospital I think because so many people were just coming into our room and hurting him he was just really upset with everybody… and I'm becoming emotional. Yeah, so even when people came to our house he was really scared of everybody so that kind of ruined our everyday life because we couldn't have friends over 'cos he'd just scream constantly at people and I think because having the nurses at home as well he couldn't trust anybody coming to our house.[BCH F.W.]

Several participants described feelings of anxiety related to general uncertainties.

Night sweats, worrying about all sorts, just want, you know, really just completely in the dark about what was going to happen and I think because there's so much forewarning that it [infection] can happen, you know, then the worst does. It's a very upsetting thing to go through. [C&W 04]

Several participants talked about the importance of having a positive mental attitude throughout the process in order to cope with what was happening to them. Some remarked that this was something that they felt that they had to actively work to achieve.

> *I've tried to stay positive, stay mentally positive the fact that it could have been worse, it could have been a lot worse, yes its happened, I can't do anything about it… From day one I was trying to get a positive mental approach on it and on that second staff infection I have to admit I was starting to get down with it because I had tried and tried and tried to… I was on the sofa for four weeks without even really moving just to try and let the skin heal and seal and maximise its recovery. So that was tough but I still stayed positive all the way through but at the end, where I was… I think I went back on the 12th of September so June, July, I was probably two and a half months on those last couple of weeks was just- was an emotional struggle of just… just get to a stage where you can only stay positive so long (laughs) before I started to get a little bit down on the second staff infection, that it got to a stage where I was just thinking I've had enough of this now, I just want it to go away so I can get back on with day to day life.[NBT 012]*

> *With burns, what you don't realise is that as horrific and as awful as it is, if you've got that mentality you won't let anything get in your way.[C&W 02]*

It was clear that participants' mood states during recovery were not constant and many reported having periods where they felt very down.

> *But it is a long- you just don't… I have really down days where I think nothing's ever going to change and I get really frustrated. [NBT 013]*

In the longer-term, there was variability among participants with respect to how well they were coping. Many participants reported that they were coping well emotionally. The end of active treatment and/or a return to normal functioning was sometimes associated with a more positive mood state.

> *Yeah, when he knew we didn't have to come back anymore he was really happy and the sleeping got a bit better, so he used to really panic about coming in 'cos it would hurt. [BCH 018]*

> *.. once I got back to work and got back to the gym and all the day to day things, I'm totally fine with it now. Totally fine with it. [NBT 12]*

Some participants described feeling more cautious or fearful of situations that were similar to those in which the injury occurred.

> *and I would test it like eight times and he will be really- won't get near anything if it's hot. He is a bit more over-cautious with stuff. Very obsessive but- So that might be a long-term thing that comes from it. [BCH 18]*

Psychological impact on significant others (family and friends)

Many participants talked about the psychological impact that their injury had had on significant others. Family members often experienced distress at the time of the injury. Some parents expressed guilt that their child had been burned.

> *once we got to the medical tent we were just screaming so I just felt really guilty I must admit, that he'd burnt himself. And to be honest I should know better. Hot drinks. And I felt really bad 'cos I'm vegan so they had milk in a hot chocolate and I'd normally test but I didn't test it. [BCH 18]*

The psychological impact on other family members continued beyond the short-term.

> *sleep-wise as well, (female sibling) was waking up in the night, obviously she's 11 so she normally sleeps fine, and she was waking up in the night and having nightmares and putting lights on and (son, patient) didn't sleep so between them both I was up every hour pretty much for weeks and then obviously I had panic attacks… it all went a bit…[BCH 018]*

Several participants also discussed how family or friends had been a source of support to help them cope with what has happened to them.

> *my mates do the usual reminders of sending me stupid videos of people having vapes exploding in their pocket and this catching on fire because that's what blokes do don't they really? … and the canoeing trip that we went on, they had a playlist of everything, Come on Baby Light My Fire, its just everything but that's what blokes do. But that's their way of making sure, I know it sounds stupid, making sure I'm emotionally alright because if I'd had turned around and told them to turn it off or punch them in the face they would realise that I'm not taking it well at the moment so…[NBT 012]*

Return to normal functioning/ability to perform daily activities

The desire to return to their normal levels of functioning was a very common theme amongst participants. In the short-term period immediately after the injury, this was often about being able to perform basic tasks of self-care. Participants described difficulties feeding themselves, moving around, using the bathroom. These activities were difficult to carry out because participants were unable to move the affected area or suffered too much pain when doing so. Tiredness and feeling weak were also identified as limiting factors.

> *particularly bathroom and (inaudible), I absolutely hated the bedpans but I was too weak to get out of bed- [C&W 06]*
>
> *The eating was very difficult and I had to be fed through a tube which I really didn't like.*
>
> *Yeah, I really didn't like being fed through a tube but then eventually when they took it out I was so weak I couldn't actually feed myself. [C&W 06]*

Some participants were also worrying at this stage about being able to perform activities in the future such as being able to return to work or education.

> *I was concerned with my hands because firstly I work with my hands. These, all these three fingers, the skin was right off down to the raw bone, seriously raw flesh on this where it had all come off. And obviously both palms, you can't really see it now because they've healed so well but I was concerned with my hands to start off with because that is my livelihood.[NBT 012]*

For patients who were hospitalised following their injury, the ability to be able to perform basic care tasks was also linked to being able to be discharged from hospital and return home.

> *like cooking, shopping and, yeah, so all the practical things, like (hospital) tried to help me with that before I left and then I went to (burns unit) and I had to live in a flat on my own but that never worked out 'cos somebody was in the apartment so I never actually (inaudible). So if I'm honest I didn't feel fully prepared at the time but anyway, that was (inaudible) it's just the everyday practical stuff and washing, that was difficult, getting in and out of the bath [C&W 06]*

In the weeks following the injury, many participants still found that their injuries limited their abilities to perform basic tasks. Several participants described having to make adaptations, take extra time or have help in order to function.

I had to wake up at five thirty because getting dressed was really difficult for me. Although it's not too far, obviously back then my arms and my hands couldn't move as well as it could today, they were still very rigid sometimes, they didn't move very well and if I wanted to unbutton my shirt, 'cos the dress code was smart, if I wanted to button up my shirt it took me a good ten, fifteen minutes to work on the buttons [C&W 02]

I made- I had like an extendable backscratcher and I Sellotaped- I got a bit of plaster tape off the nurses (laughs) and Sellotaped my fork to it so I could-[NBT 08]

Yeah, (partner) would usually cut everything up and then I would have my scoop and- [NBT 08]

In the medium to long-term, participants described how their injures impacted upon their ability to take part in recreation or leisure activities. Some participants talked about how limited movement of the affected area or fear of causing damage to their healing wound restricted their ability to play sports. Swimming was also commonly identified by participants as an activity that they were not confident to do. Concerns about chlorine stinging or the visibility of their scars when in a swimsuit were mentioned.

At the moment I've got a friend who does some swimming, some aqua aerobic and I used to do some aqua aerobic with her but I don't think I would now because at first I was worried about chlorine in the water, but then after it's not just for that, it's as well the look of it.[NBT 007]

A lot of participants talked about how their burn limited their ability to go out in the sun. This impacted on playing sports, swimming and also, for some participants, their holiday choices. Several participants mentioned having been advised to keep the area covered to protect it from sun damage, but for some participants they were also motivated to keep the area covered in hot weather due to concerns about appearance.

Just occasionally, you know, like yesterday, I took the grandchildren to the park, finding a seat that wasn't in the sun because I'm anxious- if I feel the sun on it I feel anxious because I've been told at least a year but it's going to be more than a year 'cos its obviously it is more than a year.[C&W 005]

You need to wear sunscreen wherever you go and now I have to wear double sunscreen not only for the burns 'cos it can burn but like 'cos of the discoid lupus and vitiligo could get worse. You can't go to beaches, you can't go out in the heat, your body can't tolerate certain things.[C&W 02]

Moving into the long-term, some participants talked about how they felt they coped much better psychologically once they were able to return to their normal activities. For others, the extent of their injury meant that they would never return to their pre-injury level of functioning and this was harder to accept.

so, you know, four, five months ago but over the last two or three months I've really been just back in normal life and probably the things I had decided are important have now disappeared into the (inaudible) and the normal functioning life and its less mulling over existential questions and just getting on with the day to day tasks. [C&W 04]

I think what troubles me now I've got carers and that means I've got to go and ask them and I've never been a person- [NBT 10]

Physiological effects of burn or treatment

Temperature – several participants reported feeling too hot in hospital in the short-term. Temperature problems were an issue in the longer term for a few patients. Two reported rapidly changing temperatures.

For one participant, the loss of sweat glands has caused long-term permanent issues with temperature control.

Thirst – feelings of thirst and dehydration were reported by several hospitalised participants.

Tiredness – tiredness an issue in the short-term but persisted beyond that for some interviewees.

Nutrition – Several participants mentioned that staff had impressed upon them the importance of eating to help with their short-term recovery. They also described issues they had with using feeding tubes or a lack of appetite. In the longer-term, one participant reported having lost a lot of weight and a second described speaking with a nutritionist to get advice regarding optimum nutrition for healing.

Length of hospital stay

Participants whose injuries necessitated a stay in hospital expressed differing views about the length of their stay. Some participants found it difficult and expressed the desire to go home.

> *For me I would- the thing that brought me down the most was having to be in the hospital- [NBT 08]*

> *Oh no, not that I wanted to get out of the hospital but it's not nice being- I mean they're very good, I mean I couldn't have been in a better place, they're absolutely amazing but you do don't you naturally want to go home? [NBT 014]*

One participant described how she refused to be admitted and insisted on receiving treatment (antibiotics) at home instead.

> *so they wanted to keep me in the hospital but I refused and I managed ok at home 'cos of antibiotics at home because I didn't want to stop in the hospital. But my first initial thing here I was upset because they wanted to keep me in hospital. [NBT 019]*

For others, the length of hospital stay felt just right or was experienced as a positive.

> *It didn't really bother me particularly because I had all the- I just slept and I was feeling so ill about it all and, um, and I just chatted to visitors and stuff like that but I think if I was in any longer I would have gone a bit stir crazy. I was getting to the point where I was like no I just need to go home now. [NBT 002]*

> *Like being in hospital I found, even though it's kind of a long time and everyone is kind of like I want to go home, I think being in hospital a long time is handy. I don't know if (inaudible) but I wouldn't have wanted to go home any earlier.[BCH F.W.]*

2. Interviews with professionals

Initial treatment, preventing infection, promoting healing and reduce scarring

Interviewed staff members mentioned a range of factors that are crucial around the time patients are admitted to hospital. Staff discussed reasons for importance of these factors which can jointly be seen as a chain reaction of events where each is an important influencing factor for the next.

To begin with, staff involved in the treatment of smaller burns were mostly worried about adequate management of the injury and caring for the wound during the very acute phase. This included a quick and smooth process of assessment, cleaning, scrubbing and dressing.

> "[…] they need initial scrubbing, whether that be in theatre or in the ward, and getting them dressed up so that's the initial phase I would say is only to do acutely without thinking anything else." [BCH012]

Further prompting revealed that the underlying reason is the prevention of infection. Infections were mentioned by the majority of staff as the most important concern at the point of admission to hospital.

> "For infection, just to make sure there's no infection. If there's a lot of dead skin hanging around or blisters aren't de-roofed you could potentially get an infection just in the fluid that's still around or the dead skin." [BCH001]

Staff often mentioned that the prevention is important to avoid prolonging the healing process. Healing was seen as an important outcome, especially in connection with smaller burns.

> "[…]or that they do get a small infection, you know, the process is delayed then isn't it and the healing time is prolonged" [MH001]

> "[…] quite often we're very focused on dealing with the physical aspects of the burn injury, so making sure the burn wound heals, preventing infection. […] we're very focused on managing that wound and […] making sure the wound heals […]. [BCH014]

> "Infection is what we keep an eye out for and that the right dressings are going on to maximise healing." [BCH013]

> "[…] having them being cleaned and assessed and then you kind of do think about the healing as well because they're smaller, they'll heal within two weeks and then you kind of do think more about the healing […]" [BCH012]

Whilst scarring was not important for staff at the acute stage of the injury, it was often mentioned by staff as one of patients' major concerns. Participant BCH014 described the whole chain of events nicely:

> "You know, or if you're going along that journey of the wound healing, you know, sometimes you can come into problems like infection, for example. If there's anything delaying healing, then scarring can obviously still happen. […] And that's something that is important to us is dressing the wound in a way that will kind of protect the wound [...] [a]nd help it heal quickly." [BCH014]

Pain and distress management

Almost all staff considered pain management a vital part during the acute phase of injury treatment. For smaller burns a similar chain reaction of events was observed, where the underlying reason to control pain was to reduce the distress that patients and their carers were subject to.

> "Make things worse in time... in terms of like dealing with their pain management, can be quite distressing for the family. So I think feelings of, yeah, distress for the parent and in terms of managing that child's pain is probably the most paramount when they first see us.

> […] I've... remember once I had one parent who did panic when I was cleaning the wound, and wasn't quite expecting for me to be removing skin. So she felt as though I was doing something that I perhaps shouldn't..." [BCH014]

Further prompting elicited that reduced psychological trauma was a key motivation to manage the distress.

> "Because being burnt is painful and we scrub the wound and take the blisters off which is very painful. And the children need to come back for repeat dressing changes so we need to make the first one as good as it can be. It's never nice but we need to make it as well managed and painless as possible." [BCH013]

Being in control

A small number of staff mentioned that feeling in control of the procedural steps and the situation is most important to them to ensure a high standard of burns treatment delivery. Participants mentioned the importance of underlying factors such as being well prepared for the treatment or presence of a well-functioning team.

> "It's being in control of that procedure I feel as a clinical professional, making sure that, yeah, that if you lose- I think once you've lost the control of the procedure in some way then you've lost the parent confidence in you." [*BCH006]*

Other consequences of a potential lack of control were acknowledged by two staff members. These included personal impacts on self-confidence, patients' confidence in the treatment and potential further causes of stress. In this sense, this theme may be linked to previous findings where interviewees mentioned that a well-managed procedure also contributes to reducing distress (see also quote from BCH013 above).

> "You don't want them to leave distressed and overwhelmed by it all. And then in the future you know that they're not going to mind so much coming back for future dressings so it just helps" [BCH001]

Survival

Interestingly, only two members of staff (BCH007, NBT006) mentioned survival as the most important outcome and BCH007 noted "*the most important outcome is survival because there are those patients that are very poorly and you just want them to survive*". This is only applicable for severe and larger body surface burns.

Facilitate independence from healthcare

Following discharge, it was important to the majority of staff to help patients gain independence from the care of the hospital. Several sub-themes emerged that could contribute to facilitating independence.

Self-management of wound

A major concern for staff was that patients are able to manage their wound when returning home. This included continued appropriate wound care to prevent further scarring and getting the wound to "a normal state" [MH002].

> *"I would say our main concern is will the families be able to continue with the treatment that we've taught them. So, it might be creaming and massage […] and protecting it from the sun." [BCH30102019]*

> *"[…] making sure that we instruct them […] on the massage and moisturisation at home, which they should be doing several times a day to kind of help." [BCH014]*

This included the self-efficacy and confidence to carry out the wound treatment and providing the tools and information to carry it out in the right way (e.g. how to do the dressings, cleaning etc).

> *I guess that they feel equipped and empowered to be the main carer for their child [BCH006]*

to take ownership of their own wounds from the outset so that they can manage their own lives 'cos of course they've come here, you have these rather complicated looking dressings put on and everything looks kind of amazingly difficult and complicated whereas commonly it's really straightforward and a lot of what we do isn't necessarily super sterile, its clean and these are things often times that can be managed very well at home with a bit of support from family [MH001]

Patient knowledge and understanding

At the point of discharge, it was considered critical that patients are given the advice needed so they would be *"well equipped with the information that they need" [BCH006]* to help patient understanding of the wound and treatment. This encompassed process-related information related to the self-management of the wound as described above.

> *"It's explaining to them what they need to be doing to achieve this wound to heal, and like I say before, being honest about it" [NBT026]*

> *I think that's quite important to pass that on and advice about sun protection is really important because it's new skin so that they need to be advised about that as well. [BCH008]*

Staff repeatedly mentioned it was necessary to ensure that patients understand signs of infection and closely monitor the wound for e.g. redness, swelling or changes of the wound and starting to feel unwell.

> *"It's just knowing that the patient understands how to look after their wounds, and to look out for signs of infection […] if they start [t]o feel unwell and it's their understanding of that really" [NBT026].*

It was vital for staff to emphasise that patients' understanding includes the management of their expectations with regards to the outcome of their injury.

Knowledge of availability of support

After discharge, staff frequently mentioned it was important to them to communicate the constant support available to patients and when to make use of it.

> *"My biggest goal and concern for them is can they access everything they want to access." [BCH007]*

> *"Identify when they need help and be able to just call us and we would say come back straight away if there's a problem" [BCH006]*

Independence from healthcare was less of a priority as time progressed, however, availability of psychological support was mentioned as important in the long-term and is further described in the 'Psychology' theme.

Discomfort during healing

Concerns about contractures and the desire to improve any tightness of the skin were common concerns for larger burns.

> *"So giving them a contractual release to allow them to return to as normal a state as possible, even if it's not specifically function in terms of what they do but how they can stand, how they can hold their heads" [NBT001]*

A minority of staff were concerned about patients' pain control after discharge or experiencing itching.

> *"When they heal can get quite itchy, so by putting the moisturiser on, keeping it... you know, preventing it from drying, scabbing, you are going to help that itchiness." [BCH014]*

“We want them to go home pain-free, or very near pain-free, where they’re able to control their pain at home”. [NBT026]

Other interviewees had different opinions and focused solely on self-management of the wound:

“I mean that’s the only real priority there is then because they shouldn’t be in any pain, they’re not going to need any further follow-up so it’s just making sure that they manage the healed wound” [BCH006]

Psychology

A large number of interviewees mentioned the importance of potential psychological impacts in the long-term.

“I mean long-term the important outcomes really are how they are psychologically” [BCH30102019].

“I suppose what I’m saying is the psychology, […] can be long-term, so we would be perhaps seeing families where could be a long time previous that the injury… when their children were toddlers and then they perhaps go into puberty and if they’ve had chest scarring or whatever or where teenagers are wanting to wear strappy tops or bikinis or proms, that kind of thing, it all floods back and that’s when could be years ago that they had the actual burn but we would then perhaps get involved.” [BCHRP30102019]

“I think their general wellbeing and happiness is also a long-term goal.” [BCH007]

“In some, you know, the injuries that had really knocked someone for six and kind of really turned their lives upside down in the process.” [NBT0004]

Psychological wellbeing and impact of injury & scarring

In particular, concerns were raised most frequently in connection with how the injury happened or the appearance of the scar (cosmesis). Probing revealed several aspects affecting the psychological wellbeing. Specifically, underlying internal (e.g. self-esteem, acceptance and normalisation of the injury) and external influences (e.g. bullying, worry related to intimate relationships and other people’s views) were mentioned by staff.

“I would say cosmesis is the most one ‘cos burns can be disfiguring, they can leave scars which are not very nice and not very slightly, so it affects patients psychologically and in kind of their social groups as well and also the parents as well.” [BCH012].

“I’d be still cautious about psychology and how they’ve coped, especially if it is going to scar, what impact that’s going to have on their self-esteem and bullying potentially at the school.” [BCH013]

“Some people cope with it, some people don’t. Everybody’s different, and just giving them coping mechanisms for the community really.” [NBT026]

“Definitely anxiety… yeah. Just the whole impact of the injury, I think it’s just frightens them in many ways.” [BCH008]

In particular, staff thought it was important that psychological support was available in the long-term which recognised signs of PTSD (e.g. sleeping problems, flashbacks and impacts on relationships).

[…] that psychologically they’re supported.”

"I suppose it's some sort of way of keeping an eye on people without being intrusive so that if stuff emerges down the line […] It's about, A, if they're still getting post traumatic symptoms and, B, the impact it's having on them and if, for example, […] they're frightened to go to sleep or it's affecting their relationship or whatever then you'd start to pay more attention." [NBT022]

Impact on families and daily activities

Professionals often mentioned the psychological impact of the burn on daily activities.

"[…] even if they haven't got scarring but then just integrating back into going to school, that might be hard so that could impact their lives that way." [BCH30102019]

When the injury involved children, staff highlighted the added psychological impact on parents and the family as a whole.

"So I think there's a huge psychological aspect on all the patients and actually the mobility and the functional side is only the more severe side of a patient 'cos obviously the less injured patients will get better very quickly from the function and mobility point of view and it's never a problem. So I think there's, yeah, psychological with all of them and mobility and function with most of them I'd say." [BCH007]

"it depends how the injury happened, if there's any like psychological impact on either the child... I mean, quite often the children we see are under the age of five and they don't often remember what happened. But there can be quite big like load of guilt on either the parent... if it involves the parent or quite often involves grandparents who have put, you know, a cup of coffee on the side and the child has grabbed it and sort of tipped it down themselves, if it's created... if it caused a large burn and a large injury, and they had quite a big treatment period in hospital, then that person, the grandparent or the parent, can have a lot of guilt and a lot of distress around that situation. So quite often it's, I guess, dealing with that." [BCH014]

Functional impacts

Impacts of the injury (or scar) on how it affects patients returning to normal function was an important concern for staff after discharge and in the long-run.

"Well to be living as normal a functioning, happy life as possible after having a burn injury." [BCH006]

"Function essentially, yeah […] Returning to their pre-burn level of function." [MH003]

Return to normal activities

One of the most important outcomes for staff during the medium-term (for smaller burns) and in the long-term (for larger burns) was that patients can return to their normal activities. This predominantly included the ability to return to work and school, and carry out day-to-day tasks and hobbies or other leisure activities.

"It's just encourage them just to get back into normal life now, just carry on things as normal." [BCH008]

"The most important outcome is, for me personally and professionally, it's their physical outcome. So it's are they walking, are they active, are they engaging in every activity that they would want to engage in." [BCH007]

"So initially I think it's kind of their return to their sort of family roles and their normal activities, when can I go back to work, when can I do my leisure activities." [MH002]

Movement restrictions and contractures

Staff directly involved in the treatment of burns affecting relevant areas (OT, Physios) were worried about the impact of the wound on movement.

> *"[…] hoping that the burn isn't affecting them anymore and that it is all settled down and, yeah, just living a normal life. [BCH008]*

> *"[…] the focus is very strongly on functionality. In other words my scar is preventing me from doing this, can you sort my scar out." [NBT001]*

> *"I would say mainly functional is the later one. […] I think the ultimate aim is them to be not functionally restricted with the scars and the scar that is down and not very angry and almost back to normal skin. " [BCH012]*

> *"Long-term would be the scarring and whether contracture is affecting movement" [BCH013]*

Specific location of burns were frequently mentioned as requiring additional attention because they affect mobility.

> *just being mindful [...] that [...] if the burn injury went over a certain area, so if it goes over a joint, sometimes we might book [...] an appointment with the OTs anyway, like three months down the line.[BCH014]*

> *"And then… especially around joints, so they lose their mobility of their joint as well, and it may cause… maybe, you know, they've been quite into their sport, and that may impact, so they… they can't any longer play the sport that they were doing before, or maybe the job…" [NBT026]*

Scarring and continued scar management

A commonly mentioned important long-term concern for staff was the level of scarring and associated treatments to manage it.

> *"I think scarring probably is like the biggest." [BCH014]*

Worry about scarring was twofold: staff were concerned about the functional impacts in the medium to long-term as well as the appearance of the scar.

> *"I think the ultimate aim is them to be not functionally restricted with the scars and the scar that is down and not very angry and almost back to normal skin" [BCH012]*

Concerns about the appearance were determined by patients' perception and worry about scarring, linked to potential psychological impacts.

> *"From a clinician's point of view it's a great outcome, her scar wasn't lumpy or bumpy, there's not really much there that we can see but from their point of view it affects them and how they cope, especially in teenage years as well." [BCH012]*

For larger burns, getting the wounds to "their optimum" [BCH30102019] is a priority for staff involved in the ongoing longer-term management of scars. This includes the appropriate treatment under the scar management path, e.g. pressure garments or surgery to "just get[…] the best outcome from that injury that you can." [BCH30102019]

> *"scarring, needing scar management help, perhaps like pressure garments or steroid creams" [BCH001]*

> *"So in terms of therapies by scar management teams and maybe pressure garments, silicones, that sort of thing, and then ultimately potentially like steroid injections or some form of surgery […]." [BCHRP30102019]*

Psychological effects of the continued and potentially years-long treatment were also acknowledged by staff.

> *"So, not only do they have to get over that initial situation, they have to live with their… their scars and ongoing surgery, and things like that. So some people it's lifelong." [NBT026]*

> *"The size of the injury is important to a certain extent and the amount of scarring someone's going to have and the potential reconstructive surgery because of course we're potentially re-traumatising people or people are having new things that they've got to kind of cope with." [MH002]*

> *"Some children have a really tough journey in that way, for years, who are under scar management, under therapy for repeated […] reconstructive surgery [BCH006].*

3. Combining themes

Information from the descriptive report were compared and contrasted and eventually condensed into combined themes. During this process, decisions were made to combine, separate or discard themes and suitable labels were agreed in team discussions. Tables S4 and S5 list the identified themes, how these are linked to combined themes and which outcome domains could be mapped to themes' conceptual content.

Table S1: Patient interviews themes, combined themes, and mapped outcome domain

**Themes (sub-themes)**	**Combined themes**	**Outcome domains (outcome category) as per outcome classification by Young et al (2019)**
Scarring	Scarring	• Scar texture (scar-related)<br>• Scar colour (scar-related)<br>• Scar size (scar-related)<br>• Appearance (patient-reported)
Pain	Pain and discomfort	• Burn wound pain (patient-reported)<br>• Pain during procedures (patient-reported)<br>• Itch (patient-reported)<br>• Scar pain (patient-reported)<br>• Effect of scar on movement [contractures] (patient-reported)<br>• Comfort of dressings (patient-reported)
Tightness		
Itching		
Infection	Infection	• Burn wound infection (complications)<br>• Sepsis (complications)
Appearance of the wound	Healing	• Burn wound healing (pathophysiological)<br>• Appearance (patient-reported)
Time to healing		
Psychological effects (Psychological impact on significant others [family and friends])	Psychological wellbeing	• Psychological well-being (patient-reported)<br>• Generalised anxiety (patient-reported)<br>• Mental ability (patient-reported)<br>• Quality and quantity of sleep (patient-reported)

Return to normal functioning/ability to perform daily activities	Function	• Ability to carry out daily tasks (patient-reported)<br>• Return to work/school or previous function (patient-reported)<br>• Appearance (patient-reported)
Uncertainty, lack of knowledge and the need for information	Uncertainty	None identified
Length of hospital stay	Discarded	
Physiological effects of burn or treatment	Discarded	

Table S2: Professional interviews themes, combined themes, and mapped outcome domain

**Themes (sub-themes)**	**Combined themes**	**Outcome domains (outcome category) as per outcome taxonomy**
Initial treatment, preventing infection, promoting healing and reduce scarring	Healing	• Burn wound healing (pathophysiological)
	Infection	• Burn wound infection (complications)
Pain and distress management<br>Discomfort during healing	Pain and discomfort	• Use of medicines to treat symptoms (healthcare-related)<br>• Burn wound pain (patient-reported)<br>• Effect of scar on movement [contractures] (patient-reported)
Scarring and continued scar management	Scarring	• Treatment for scars (scar-related)<br>• Scar texture (scar-related)<br>• Scar colour (scar-related)<br>• Scar size (scar-related)<br>• Appearance (patient-reported)
Functional impacts (Return to normal, Movement restrictions)	Function	• Mobility (pathophysiological)<br>• Ability to carry out daily tasks (patient-reported)<br>• Return to work/school or previous function (patient-reported)
Psychology (Psychological wellbeing and impact of injury & scarring, Impact on families and daily activities)	Psychological wellbeing	• Psychological well-being (patient-reported)<br>• Generalised anxiety (patient-reported)<br>• Mental ability (patient-reported)<br>• Quality and quantity of sleep (patient-reported)
Survival	Survival	• Death from burn injury (complications)<br>• Death from any cause (complications)
Being in control	Sense of control	None identified
Facilitate independence from healthcare (Self-management of wound/ independence, Patient knowledge and understanding, Knowledge of availability of support)	Patient knowledge, understanding & support	None identified
