# Supplementary material for: Exploring what is important during burn recovery: a qualitative study investigating priorities of patients and healthcare professionals over time
Source: BMJ Open. 2023 Feb 10;13(2):e059528. doi: 10.1136/bmjopen-2021-059528 (PMC9923305; doi:10.1136/bmjopen-2021-059528)
Supplement: Author's manuscript [file bmjopen-2021-059528.draft_revisions.pdf]

BMJ Open

BMJ Open is committed to open peer review. As part of this commitment we make the peer review history of every article we publish publicly available.

When an article is published we post the peer reviewers' comments and the authors' responses online. We also post the versions of the paper that were used during peer review. These are the versions that the peer review comments apply to.

The versions of the paper that follow are the versions that were submitted during the peer review process. They are not the versions of record or the final published versions. They should not be cited or distributed as the published version of this manuscript.

BMJ Open is an open access journal and the full, final, typeset and author-corrected version of record of the manuscript is available on our site with no access controls, subscription charges or pay-per-view fees (http://bmjopen.bmj.com).

If you have any questions on BMJ Open's open peer review process please email info.bmjopen@bmj.com

BMJ Open

Exploring what is important during burn recovery: A qualitative study investigating priorities of patients and healthcare professionals over time

Journal:	*BMJ Open*
Manuscript ID	bmjopen-2021-059528
Article Type:	Original research
Date Submitted by the Author:	23-Nov-2021

Complete List of Authors:	Hoffmann, Christin; University of Bristol, Population Health Sciences<br>Davies, Philippa ; University of Bristol, Population Health Sciences<br>Elliott, Daisy; University of Bristol, Population Health Sciences<br>Young, Amber; University of Bristol, Population Health Sciences; University Hospitals Bristol and Weston NHS Foundation Trust

Keywords:	Quality in health care < HEALTH SERVICES ADMINISTRATION & MANAGEMENT, QUALITATIVE RESEARCH, Clinical trials < THERAPEUTICS

SCHOLARONE™
Manuscripts

I, the Submitting Author has the right to grant and does grant on behalf of all authors of the Work (as defined in the below author licence), an exclusive licence and/or a non-exclusive licence for contributions from authors who are: i) UK Crown employees; ii) where BMJ has agreed a CC-BY licence shall apply, and/or iii) in accordance with the terms applicable for US Federal Government officers or employees acting as part of their official duties; on a worldwide, perpetual, irrevocable, royalty-free basis to BMJ Publishing Group Ltd ("BMJ") its licensees and where the relevant Journal is co-owned by BMJ to the co-owners of the Journal, to publish the Work in this journal and any other BMJ products and to exploit all rights, as set out in our licence.

The Submitting Author accepts and understands that any supply made under these terms is made by BMJ to the Submitting Author unless you are acting as an employee on behalf of your employer or a postgraduate student of an affiliated institution which is paying any applicable article publishing charge ("APC") for Open Access articles. Where the Submitting Author wishes to make the Work available on an Open Access basis (and intends to pay the relevant APC), the terms of reuse of such Open Access shall be governed by a Creative Commons licence – details of these licences and which Creative Commons licence will apply to this Work are set out in our licence referred to above.

Other than as permitted in any relevant BMJ Author's Self Archiving Policies, I confirm this Work has not been accepted for publication elsewhere, is not being considered for publication elsewhere and does not duplicate material already published. I confirm all authors consent to publication of this Work and authorise the granting of this licence.

For peer review only

Exploring what is important during burn recovery: A qualitative study investigating priorities of patients and healthcare professionals over time

Submission category: Original research

Authors:
C Hoffmann[1], c.hoffmann@bristol.ac.uk

P Davies[1], Philippa.Davies@bristol.ac.uk

D Elliott[1], daisy.elliott@bristol.ac.uk

A E Young[1,2], amber.young1@nhs.net

Author affiliations:

[1] National Institute for Health Research Bristol Biomedical Research Centre, Bristol Centre for Surgical Research, Bristol Medical School: Population Health Sciences, University of Bristol, 39 Whatley Road, Clifton, Bristol, BS8 2PS, UK

[2] Bristol Royal Hospital for Children, University Hospitals Bristol and Weston NHS Foundation Trust, Upper Maudlin St, Bristol BS2 8BJ

Correspondence: Christin Hoffmann [c.hoffmann@bristol.ac.uk]

Running title: PRIORITIES OF BURNS STAKEHOLDERS OVER TIME

Keywords: Burns; Qualitative; Outcomes; Outcome measurement

Word count: 4993 (excluding title page, abstract, tables and references)

For peer review only - http://bmjopen.bmj.com/site/about/guidelines.xhtml

Abstract

Objectives: This qualitative study aimed to investigate what is most important to patients and healthcare professionals during recovery from a burn injury.

Design: Semi-structured interviews were conducted. Interviews were audio recorded, transcribed and analysed thematically.

Setting, participants: A total of 53 patients and healthcare professionals were recruited from four NHS burn services across England and Wales across England and Wales. Patient participants (n=32) included adults, adolescents and parents of paediatric patients, with a variety of burn injuries in terms of severity and cause of burn injury. Healthcare professionals (n=21) were NHS staff members involved in burn care and included professionals with a range of clinical experience and roles (e.g. nurses, surgeons, occupational therapists, physiotherapist, administration).

Results: A total of ten themes relating to priorities during recovery from a burn injury were identified for patients and professionals. Of those, six were identified for both stakeholder groups ('pain', 'psychological wellbeing', 'healing', 'scarring', 'function' and 'infection'), three were unique to professionals ('patient knowledge, understanding & support', 'sense of control', 'survival') and one was unique to patients ('uncertainty'). Results highlighted that importance of these priorities changes over time (e.g. 'survival' was a concern in the short term but not in the medium to long term). Likewise, it was revealed that priorities differed between patients and professionals (e.g. 'pain' was a concern in the short term for professionals but important to patients throughout their recovery). A total of seven themes overlapped with outcomes commonly assessed in burn research, whereas three themes could not be mapped to reported outcomes ('uncertainty', 'patient knowledge, understanding & support', 'sense of control').

Conclusion: Professionals' and patients' priorities (important outcomes) change over time after injury and differ between stakeholder groups. Burn care research should take into account the time-sensitivity of routinely measured outcomes to accurately reflect complexity of burn recovery.

(295 words)

For peer review only - http://bmjopen.bmj.com/site/about/guidelines.xhtml

Strengths and limitations of this study

- This work compared perspectives of two stakeholder groups (i.e. patients and professionals) regarding the importance of priorities after burn injury
- This study, to our knowledge, is the first to provide empirical evidence of time sensitivity of outcomes after burn injury
- The study recruited a diverse sample of stakeholders from four NHS burn services across England and Wales to elicit a wide range of perspectives.
- The results are relevant to patients with all types of burn injuries, but a more homogeneous sample of burn types may provide more detailed and nuanced insights into priorities of specific groups of patients.

For peer review only

For peer review only - http://bmjopen.bmj.com/site/about/guidelines.xhtml

INTRODUCTION

Each year, approximately 10,000 patients with burn injuries are treated in UK NHS burn services [1]. Burns can affect many aspects of patients' lives, including their function, cosmesis and psychological well-being. Injury management incurs challenges due to their varying complexity (e.g. burn size and location) and factors unique to patients' personal circumstances (e.g. age, profession) [2]. On-going development of treatments to improve outcomes (e.g. quality of life, scarring) means that evidence for optimal strategies for treating burns is still evolving [3]. Accurate measurement of patient relevant outcomes to evaluate strategies is key to achieving high-quality, patient-centred care [3,4].

The importance of relating stakeholder perceptions to measurable patient-relevant outcomes in burn research has been recognised [5–7]. Burns can impact affected individuals and their families in the short and in the long term in different ways [8]. For larger burns, for example, survival can be the main priority in the acute stages of treatment, whilst scarring may be a long term concern. It is probable that perceptions about what matters most to patients and professionals may vary between groups and over time. Consequently, the importance of outcomes may also vary. Current methods in burns research, however, commonly assume equal importance of outcomes at a specific time for all stakeholders. Measuring a certain outcome at a certain time might not, however, provide a true picture of patients' recovery, and decisions about optimal treatment may therefore be ill informed. It is unclear how specific priorities differ across time and stakeholder group as recovery from a burn injury progresses.

There is a need to identify which priorities are important at which time points during burns recovery. This may facilitate optimisation of outcome measurement and ensure evaluation of treatment strategies is patient-centred. Qualitative research is important in burns care [9] and has been widely used to explore perspectives and concepts important to burns survivors [3,6,10]. Previous work has shown that (mostly objective) clinical outcomes thought to be important, may not align with patients' views and perspectives [11]. It is therefore important to understand perspectives of professionals *and* patients as both need to play a role in making decisions about treatment and care [12,13].

Aim

The aim of this qualitative study was to explore what is most important to patients and healthcare professionals during recovery from a burn injury by eliciting perspectives over time.

For peer review only - http://bmjopen.bmj.com/site/about/guidelines.xhtml

METHODS

This study uses qualitative methods, employing in-depth, semi-structured interviews to explore participants' perspectives to:

i. Investigate priorities of burns patients (including adults, adolescents and parents of paediatric patients) and healthcare professionals during burn recovery and explore how they differ.
ii. Explore how priorities of patients and healthcare professionals change over time.
iii. Map identified themes to outcomes currently reported in burns research.

Conduct and reporting of this study adhered to standards for reporting qualitative research (SRQR) [14]. This study adopted an interpretivist approach to understand participants perspective, acknowledging that experiences are unique and perceptions construed within individual's particular setting [15].

Setting and participants

This study recruited two groups of stakeholders (patients and healthcare professionals) from four NHS burn service across England and Wales. Three were adult services and one specialised in paediatric burns. Patient participants were NHS patients with lived experience of burn injuries. These included adults (of 16 years and older), adolescents (10-15 years of age) and parents of paediatric patients (of less than ten years of age) (henceforth simplified to 'patients'). All patients were more than 30 days after burn injury and were not in acute treatment. Healthcare professional participants were multidisciplinary NHS employees of burn services at the selected study sites (henceforth simplified to 'professionals').

Eligible participants were approached by research nurses or clinical / academic researchers (KC, AS, CS, AY) and invited to participate in the study. Information about the study was provided and any questions were answered before written consent was obtained. A clinical (AY) or academic (CH) researcher, both trained and experienced in qualitative research, contacted participants to arrange a suitable interview method, location, date and time.

Purposive sampling was employed to ensure maximum variation between patients (e.g. age, gender, severity and cause of burn injury) and professionals (e.g. clinical experience, role). Recruitment continued until no new ideas and concepts evolved from the interviews (data saturation).

For peer review only - http://bmjopen.bmj.com/site/about/guidelines.xhtml

Data collection

Interviews with patients and professionals were completed face-to-face or over the phone, depending on participant preference. Face-to-face interviews were carried out in a quiet space in one of the four hospitals. In-depth interviews were conducted independently by two study members (AY, CH). AY is a consultant anaesthetist in paediatric burn care with 23 years of clinical experience and substantial experience of burns research. CH is a social scientist and qualitative researcher with no involvement in burns care or research prior to this study.

Interviews followed a semi-structured format using a topic guide with open-ended questions to guide discussions (see Supplementary File, Table S1 for an example). An initial topic guide was developed based on the study objectives of exploring priorities over time. To ensure consistency of explored perspectives, time periods were defined *a priori*: a) short term: immediately after the injury, b) medium term: from around the time of discharge and/or wound healing c) long term: during rehabilitation. The topic guide was piloted prior to study commencement but allowed to evolve during the study whereby iterative refinements were made as data collection progressed.

Analysis

All interviews were audio-recorded using an encrypted device, and de-identified during transcription. Random samples of verbatim transcripts were checked for accuracy by one researcher (CH).

Analysis of qualitative data was undertaken using a qualitative data management software (NVivo Version 12). In a first step, transcripts were analysed using inductive thematic analysis [16] to understand priorities of patients and professionals (study objective i). Interview transcripts were analysed separately for patients and professionals to accommodate concepts unique to each group and facilitate later examination of differences. Transcripts were read and re-read to allow familiarisation with the data. This study used deductive and inductive approaches to coding [17]. Codes for short, medium and long term priorities were developed *a priori*, to explore perspectives over time (study objective ii). Additional codes were identified through line-by-line coding following principles of inductive theme development. In a second step, differences in priorities between stakeholders were examined by comparing narrative accounts of each group. Connections were drawn between patients and professionals by qualitatively contrasting themes identified for each group to find similarities and differences in their perspectives (study objective i). Coding and analyses were undertaken independently by two researchers (CH, PD) who met regularly to discuss

For peer review only - http://bmjopen.bmj.com/site/about/guidelines.xhtml

emerging themes from the data. Close contact between the coders during data analyses ensured consistency in coding and agreement of interpretation of the data. Independent double-coding was also performed for 25% of transcripts by the two reviewing authors. A purposive sample of transcripts that underwent double-coding was used to ensure maximum variation by stakeholder group, socio-demographic background and injury severity. Data from double-coding were compared and discussed to ensure consistency in approaches to further coding. Any uncertainties and discrepancies during this process were discussed with the wider study team and in consultation with an experienced qualitative researcher who was independent to the study team (DE). Coded excerpts were grouped into themes of similar meaning and organised into a hierarchical structure of themes and sub-themes. This thematic structure was refined as new themes emerged. Interim interpretation and structure of themes was discussed with the wider study group and if necessary, iteratively modified. This process was overseen by the senior author (AY) who has a clinical background in burn care. A final thematic structure and meaning of themes were agreed in team discussions. Findings were collected in two descriptive accounts (one for patients, one for professionals) to be able to identify similarities and differences in key findings.

Themes identified through analyses described above were mapped against examples of existing outcome domains to assess whether perceptions explored in this work align with routinely measured outcomes in burns research (objective iii). A simple search using keywords related to “burns” and keywords for relevant outcomes was conducted in PubMed in November 2021 to identify articles reporting burn trials and relevant systematic review studies. Supplemental hand searching of reference lists of identified articles was performed where applicable. Identified articles were reviewed to compare conceptual content of relevant outcomes reported in studies with themes identified in this work (e.g. by exploring how reported outcomes were defined or measured to ensure that the meaning of the outcome aligns with identified themes). Search and review of articles ceased when a relevant example was identified. Any data supporting themes identified in this work were tabulated.

Participant characteristics were summarised using descriptive statistics. Data related to burn size were grouped into categories (minor, moderate, major) according to percentage total body surface area (TBSA), following established classification guidelines [18].

For peer review only - http://bmjopen.bmj.com/site/about/guidelines.xhtml

Patient and Public Involvement

This study is part of a wider project that aims to standardise outcome reporting and measurement in studies of burns injuries. Patient representatives and carers were actively involved in the design, conduct, analysis and dissemination of aspects of the wider project.

RESULTS

Participant characteristics

A total of 53 participants (32 patient participants, 21 professional participants) took part in semi-structured interviews, lasting between 15 and 45 minutes. Four patient interviews were conducted with two participants (three with young person and their parents, one included a patient and their carer). Participants were recruited from four NHS trusts between June 2017 and February 2020.

Patient participants' age ranged from 1 – 82 years (*Mean*=38 years) and 19 (59%) participants were female. The sample mainly consisted of adults (n=20 participants, 65%). Scalds were the most common burn type (n=10, 36%). Participants primarily suffered from minor burns (n=14, 50%) with six (21%) patients who experienced major burns.

Healthcare professionals sampled were mostly female (n=17, 82%) with the majority of participants in a nursing role (n=11, 52%). Detailed participant characteristics are presented by stakeholder group in Table 1.

Table 1 Participant characteristics (N=52)

	**Patients (N=32)**	**Professionals (N=21)**
**Age, mean (range)**	38 (1-82)	
**Female, n (%)**	19 (59)	17 (82)
**Occupation, n (%)**		
Consultant clinical psychologist		3 (14)
Physiotherapist		1 (5)
Consultant, registrar		2 (10)
Occupational therapist		3 (14)
Research, Specialist, Clinic or Senior nurse		11 (52)
Administration		1 (5)
**Patient type, n (%)**		
Adult (16 years and older)	20 (63)	
Young person (10-15 years)	3 (9)	
Parent (all ages)	5 (19)	

For peer review only - http://bmjopen.bmj.com/site/about/guidelines.xhtml

Parent (of person aged 10-15)	3 (9)
**Burn size, n (%)***	
Minor (<10% TBSA)	14 (50)
Moderate (10-20% TBSA)	2 (7)
Major (>20% TBSA)	6 (21)
Not reported	6 (21)
**Burn reason, n (%)***	
Scald	10 (36)
Flame	8 (29)
Chemical	3 (11)
Flash	3 (11)
Contact	2(7)
Electric	1 (4)
Not reported	1 (4)

** % data based on number of patient interviews (n=28) because this reflects the number of injuries*

Priorities of patients and professionals over time (objectives i and ii)

A total of ten themes were identified as relating to participants' priorities during burn recovery. Six themes were most important for both stakeholder groups ('pain', 'psychological wellbeing', 'healing', 'scarring', 'function', 'infection') and four themes were identified as most important to only patients or professionals (i.e., 'uncertainty', 'patient knowledge, understanding & support', 'sense of control', 'survival'). There were differences in the relative importance of these priorities over time, and differences between patients and professionals. An overview of themes and their importance for patients and professionals at different time points during recovery can be found in Figure 1. Themes are summarised below and presented in order of their prevalence in the data, ascertained by the number of coded references.

Pain

Pain was a concern common to patients and professionals in the short term. Pain was a major cause of distress for the majority of patients and their family members as a result of the injury and initial treatment.

Professionals were particularly concerned about pain and adequate pain management to prevent distress and any effects in the long term.

Some patients were worried about pain throughout their recovery. Especially, patients with larger burns,who complained about different aspects of long term pain. For example, itching, scar tightness

For peer review only - http://bmjopen.bmj.com/site/about/guidelines.xhtml

and contractures causing discomfort, neuropathic pain during nerve regeneration were all mentioned. Professionals, however, did not mention pain as a general concern in the long term.

> *"Managing the pain is the immediate thing." [professional, BCH30]*
>
> *"When it first happens it's just about the pain. That's it. […] that is just all you're focussing on at the time" [patient, NBT012]*
>
> *"So the biggest problem is sometimes, you know, a bit of pain in the leg for example, but it doesn't stop me doing things, you know, like walking but sometimes […] it is perhaps because it's tighter than it should be, I don't know, but it was more painful but its easing.' [patient NBT007]*

Psychological wellbeing

Psychological wellbeing and related impacts were concerns common for patients and professionals in the long term. Both groups' concerns were predominantly linked to scar appearance, mentioning internal (e.g. self-esteem, acceptance and normalisation of the injury) and external (e.g. bullying, worry related to intimate relationships and other people's views) influences on psychological wellbeing.

Professionals were mainly worried about long lasting impacts on patients' psychological wellbeing, manifesting in post-traumatic stress disorder (PTSD) from distress caused by injury and scarring. In particular, patients with larger burns described psychological impacts during the later phases of recovery (e.g. coping, PTSD).

For patients, the psychological effects of having a burn were apparent during all stages of recovery. In the short term, injuries affected individuals (e.g. worry and anxiety) and family members (e.g. anxiety and guilt, in particular parents). Psychological impacts were also highlighted in the medium term by patients (e.g. problems sleeping, flashbacks).

> *"I mean long-term the important outcomes really are how they are psychologically" [professional, BCH031].*
>
> *"When she saw her face she just broke down. She said 'I'm really ugly mummy'." [patient, BCH001]*
>
> *"I was just getting too upset and depressed over the fact that I had burns and I looked so different and had too many worries and thoughts in my head that I just really didn't want to think about" [patient, CW002]*

Healing

For peer review only - http://bmjopen.bmj.com/site/about/guidelines.xhtml

Healing of the wound was a common concern, expressed frequently by professionals and patients. Importance of healing across the stages of injury recovery, however, differed across participant groups.

Professionals worried about healing in the short term. This was evident through narratives about concerns relating to adequate management of the wound during the acute injury phase, including a quick and smooth process of assessment, cleaning and dressing.

Patients' worry about healing was apparent in the medium to long term of recovery and often linked to feelings of surprise, frustration or worries about the amount of time it was taking for the wound to heal. The appearance of the wound was strongly linked to perceived length of healing times.

> *"Quite often we're very focused on dealing with the physical aspects of the burn injury, so making sure the burn wound heals, preventing infection. […] we're very focused on managing that wound and […] making sure the wound heals […]. [professional, BCH014]*
>
> *"When you see it it's quite gnarly, […] like when its fresh you're like oh god, this is never going to heal" [patient, NBT002]*

<u>*Scarring*</u>

Scarring was repeatedly mentioned by both participant groups to be of importance in the long term.

Professionals were mostly concerned about appropriate scar management to optimise their appearance and avoid restrictions on movement.

Similarly, patients' worries in the later phases of recovery spanned any need for further treatment and potential impacts on movement as well as the appearance of scars influencing self-esteem and body image that could affect psychological wellbeing. For patients, the worries about risk of visible scarring were also evident in the short and medium term of the recovery. Those concerns affected individuals and their family members in terms of worry about their appearance and associated future impacts on the patient (e.g. affecting psychological wellbeing).

> *"Long-term would be the scarring and whether contracture is affecting movement" [professional, BCH013]*
>
> *"Well the worries were the scarring and whether we'd need to have- at that point in time we didn't know- no-one could tell us how bad it was." [patient, BCH001]*
>
> *Just wondering what the scarring would do, whether the skin would be very tight. Because you do see people who've had burns, like facial burns and sometimes their skin is very pink and red and then you do notice it. But yes, I think that was about it really. It's just how functional your hands would be when they're recovered. [patient, NBT027]*

For peer review only - http://bmjopen.bmj.com/site/about/guidelines.xhtml

Function

Return to normal functioning was a concern common to patients and professionals in the medium and long term. Participants discussed worries about impacts of the injury (or scar) on daily activities and how it affected patients returning to their normal lives was an important concern following discharge.

Professionals' priorities in relation to function were also focussed on aspects of mobility, primarily ensuring physical movement.

For patients, return to normal function was also a priority in the short term by highlighting concerns about the ability to return to work.

> *"The earlier you can intervene with physio then that's going to help with movement, […] it's important ongoing as well if the wounds are that bad that there is movement issues." [professional, BCH030]*
>
> *"So initially I think it's kind of their return to their sort of family roles and their normal activities, when can I go back to work, when can I do my leisure activities." [professional, NBT004]*
>
> *"I was concerned with my hands to start off with because that is my livelihood." [patient, NBT012]*
>
> *"Particularly bathroom […], I absolutely hated the bedpans but I was too weak to get out of bed" [patient, CW006]*

Infection

Infection was mentioned by both participants. Importance of infection over time, however, differed across participant groups.

Preventing infection was one of the main priorities for professionals in the short and medium term. Following patients' hospital admission, professionals highlighted the importance of adequate cleaning and preparation of the wound to reduce healing time and scarring. Worry about cleanliness extended to the medium term of burn recovery when professionals wanted to ensure *"that the patient understands how to look after their wounds, and to look out for signs of infection" [professional, NBT026].*

Patients discussed worries about infection related to the medium term of their recovery. Whilst most interviewed patients were aware of the need to keep the wound clean, infection was discussed as major concern primarily by those patients who had experienced symptoms of infection.

For peer review only - http://bmjopen.bmj.com/site/about/guidelines.xhtml

> *"Just to make sure there's no infection. If there's a lot of dead skin hanging around or blisters aren't de-roofed you could potentially get an infection just in the fluid that's still around or the dead skin." [professional, BCH001]*

> *"I thought that I had been released for good and was at home and actually I was extremely unwell. Night sweats, worrying about all sorts, just want, you know, really just completely in the dark about what was going to happen and I think because there's so much forewarning that it can happen, you know, then the worst does. It's a very upsetting thing to go through." [patient, CW004]*

Patient knowledge, understanding & support

This theme was identified as a unique priority for professionals. In the medium term, at the point of discharge, professionals felt it was crucial to ensure patients are *"well equipped with the information that they need" [professional, BCH006].* This encompassed information related to support the self-management of the wound (e.g. creaming, massaging, sun protection). Professionals' main objectives were also to help patients understand their injury (e.g. recognise signs of infection) and gain self-confidence in further wound treatment, so they remain as independent from the service as possible. Interviewees also repeatedly mentioned the importance of communicating the constant support available to patients which included readiness to answer any questions and psychological support.

> *"It's explaining to them what they need to be doing to achieve this wound to heal" [professional, NBT026]]*

> *"My biggest goal and concern for them is can they access everything they want to access." [professional, BCH007]*

Uncertainty

Uncertainty was identified as a unique theme for patients and was an important concern during the earlier stages of recovery. Feelings of uncertainty related to how the injury happened, the treatments needed and their possible outcomes as well as the likely course of recovery. Participants associated feelings of anxiety with uncertainty but also described how information provision (e.g. about recovery and further treatment) can lessen those feelings. For patients with more severe burns, uncertainties remained in the long-term and were mostly related to scar management and future treatment plans.

> *"During the time that we were kind of up in the air as to whether he would have the operation or not, we saw loads of different people on different rotas and stuff, and I kind of felt like there were lots of different opinions flying around about how well it was healing,*

For peer review only - http://bmjopen.bmj.com/site/about/guidelines.xhtml

how well it wasn't healing, what the next course of treatment would be. And although I understand why that was the case, because in the main they were kind of waiting to see what the wound was going to do, we felt very up in the air about […] what was going to happen, and whether what happened eventually would have actually been the right thing." [patient, BCH017]

"Yeah, you've got no information, no knowledge and the pain materialises, you don't know whether that's supposed to happen, was that not supposed to happen, is something going wrong." [patient, NBT012]

Sense of control

For a smaller number of professionals, sense of control was identified as a priority in the short term. The need to feel a sense of control over the treatment situation and technical steps involved was mentioned as a major concern. Participants described the importance of feeling well prepared for the patients' treatment and the presence of a well-functioning team to support a smooth treatment.

"It's being in control of that procedure. I feel as a clinical professional, […] once you've lost the control of the procedure in some way then you've lost the parents' confidence in you." [professional, BCH006]

Survival

Some professionals mentioned survival to be the key priority in the short term and was only discussed in the context of larger burns. For those professionals involved in their care, mortality-related outcomes and physiological signs (e.g. organ failure, fever) were a major concern. Only a minority of patients experienced life-threatening injuries, but those with major burns generally did not discuss survival. This may be caused by *"a kind of hole in their memory and they don't remember what happened to them" [professional, MH006]*, aggravated by severe pain relief and reduced levels of consciousness.

"Well you'd be looking at their physical signs and outcomes, things like their vital signs, their bloods, whether they're being ventilated and they're on inotropes, and also whether they're having runs of sepsis and whether they've gone into acute renal failure" [professional, NBT003]

"The most important outcome is survival because there are those patients that are very poorly and you just want them to survive". [professional, BCH007]

Mapping themes to outcomes (objective iii)

For peer review only - http://bmjopen.bmj.com/site/about/guidelines.xhtml

For peer review only

A total of seven out of 10 themes (70%) were identified as recognised outcomes in burn research. These were evident in a recently published systematic review that investigated all outcomes reported in burn care research [19] and identified research studies (see Table 2). An overview of identified exemplary studies assessing an outcome for which conceptual content aligns with themes identified in this study is shown below.

Table 2 Example studies that report outcome and that aligns with present themes

**Theme identified**	**Outcome reported and verbatim quotes from exemplary burn care research to demonstrate how conceptual content aligns with theme identified**
**Pain**	Outcome reported: Pain and distress
	Conceptual content reported: "Pain and distress were assessed by obtaining: (1) the participant's self-report of pain intensity using the Faces Pain Scale-Revised (FPS-R) (if participant was aged 3 years or over); (2) the nurse's observational rating of the participant's pain and distress using the face, legs, activity, cry, consolability (FLACC) scale; (3) the participant's self-report (if aged over 8 years) or the parent's report of the participant's pain intensity using a Visual Analog Scale-Pain (VAS-P); (4) pulse rate; and (5) respiratory rate of the participant, taken immediately prior to and after dressing changes.)" [20]
**Psychological wellbeing**	Outcome reported: Psychological functioning
	Conceptual content: "The Chinese version of the Hospital Anxiety and Depression Scale (HADS) will be used to evaluate psychological functioning (anxiety and depression). The 14-item *HADS* was designed as a tool to screen for anxiety and depressive symptoms in medical settings and has well-demonstrated solid psychometric properties for use in medical settings and commonly used in burn care settings to screen anxiety and depression" [21]
**Healing**	Outcome reported: Wound healing
	Conceptual content: "Wound healing at day 30 was assessed" [22]
**Scarring**	Outcome reported: Burns scar appearance
	Conceptual content: "Outcome measures were recorded at baseline, 6-weeks post final treatment and up to 3 years post-treatment. Measures included blinded assessor VSS, Patient Scar Assessment Scale and histological tissue analysis." [23]
**Function**	Outcome reported: Physical role functioning/performance
	Conceptual content: "The Chinese version of the Disability of the Arm, Shoulder and Hand Symptom Scale (DASH) will be used to evaluate this outcome. The DASH tool is a 30-item questionnaire that asks the patient to grade symptoms and physical function during the preceding week on a five-point Likert scale." [21]
**Infection**	Outcome reported: Wound infection
	Conceptual content: "Investigators also were required to submit documentation of complications their patients encountered during treatment." [24]
**Survival**	Outcome reported: Mortality
	Conceptual content: "Mortality referred to death from any reason" [25]

For peer review only - http://bmjopen.bmj.com/site/about/guidelines.xhtml

There were three themes (30%) to which no outcome reporting in the literature could be found. ‘Uncertainty’, ‘patient knowledge, understanding & support’ and ‘sense of control’ were all themes uniquely identified in this study [19].

DISCUSSION

This study used semi-structured interviews to investigate perspectives of 53 burn patients, and professionals involved in their care, to explore what is most important as the patients recover. The study identified a total of ten themes across both participant groups that related to priorities during recovery from a burn injury. Of those, six were identified across both stakeholder groups (‘pain’, ‘psychological wellbeing’, ‘healing’, ‘scarring’, ‘function’ and ‘infection’), three were unique to professionals (‘patient knowledge, understanding & support’, ‘sense of control’, ‘survival’) and one was unique to patients (‘uncertainty’). The study results also showed that the importance of recovery priorities changes over time (e.g. ‘survival’ was a concern in the short term but not in the medium to long term), and that priorities differed between patients and professionals (e.g. ‘pain’ was a concern in the short term for professionals but important to many patients throughout their recovery). Finally, this work established that seven identified themes overlapped with outcomes commonly reported in burn research, whereas three themes could not be mapped to reported outcomes (‘uncertainty’, ‘patient knowledge, understanding & support’, ‘sense of control’).

This work identified unique differences between what different stakeholders view as most important during burn recovery and supports the temporal sensitivity of priorities of patients and professionals. We were unable to find existing empirical evidence from burn care research that shows similar findings. This work, however, does complement other recent efforts to improve patient-centred outcome measurement in burn care research [3,6,7,10] by highlighting the need to align outcome assessment with the changing importance of priorities for different stakeholder groups, and with the change in relevance of priorities at different times after injury or intervention. Research in other healthcare areas has started to acknowledge these complexities of outcome assessment during recovery. Development of a core outcome set for childhood asthma, for example, reported the differences in perspectives of patients and professionals as to which outcomes are most important [26]. Other core outcome sets for lower limp amputation or induction of labour, for example, included separate outcomes relevant to short and medium term recovery [27–29]. This study supports a recommendation for core outcome sets in burns care to differentiate between different time points [3], but current core outcome sets do not yet incorporate these complexities [12].

For peer review only - http://bmjopen.bmj.com/site/about/guidelines.xhtml

An aim of on-going research is to ensure that frequently measured outcomes adequately capture all important priorities related to burns recovery [6]. The results of this study highlighted the importance of three themes not aligned with commonly measured outcomes in burn care research. Subjective perceptions related to 'uncertainty' (patients), 'sense of control' and 'patient knowledge, understanding & support' (professionals) were all identified as important priorities at varying time points during recovery. The existence and impacts of uncertainty on patients are well known [30] and, as supported by our findings, and have also been linked to the level of information provision [31,32]. Conversely, professionals in our study highlighted that 'patient knowledge, understanding & support' is one of their key priorities during the medium term of patients' recovery from a burn injury. Routine assessment of perceptions relevant to level of uncertainty and information provision as well as assessing patients' level of knowledge and understanding and support received can be important beyond commonly used measures. In addition, these measures of subjective perception might provide useful care quality indicators to highlight where improvements, e.g. in information provision, might be required. Measurement instruments assessing patients' sense of control in healthcare interventions exist [33,34]. Very few studies, however, report the need to feel in control from the perspectives of healthcare professionals [35], and we were unable to find a measure assessing professional specific sense of control. In our study, only a small number of professionals mentioned this as a priority which warrants further research investigating the importance and impacts of perceived sense of control.

Strengths and limitations

Our findings integrated views from a large sample of patients and healthcare professionals (n=53) from various backgrounds, allowing investigation of a variety of perspectives. This means that the findings are broadly representative of commonly occurring burn injuries but simultaneously may not account for unique concerns of specific patient groups. For instance, where burn injuries were related to mental health issues (e.g. self-inflicted injuries), patients may display different patterns of priorities. Similarly, analysis focused on the most important and most commonly raised concerns which implies that individual experiences and priorities may differ to themes identified in this study. Other limitations should be acknowledged. First, there is no consensus on the definition of time frames in burn recovery. This study defined time frames (short, medium and long term) *a priori* which were agreed after expert consultation and discussions within the multi-disciplinary study team. There are, however, challenges in standardising time frames for recovery from burn injury considering the variabilities and complexities of injuries [36–38]. For instance, patients with larger

For peer review only - http://bmjopen.bmj.com/site/about/guidelines.xhtml

burns experience longer healing [37]. This means that individual definitions of short, medium and long term recovery are highly personal and might depend on contextual complexities (e.g. size and location of burn, socio-demographic details of patients, any wound complications), and might therefore differ to the definitions used in this study. Second, our recruitment strategy meant that some patient groups may be underrepresented (e.g. patients with larger burns, adolescent patients). Likewise, professional participants were mainly female and had nursing roles and there were challenges in recruiting surgeons, mainly due to individuals' time constraints. It is also acknowledged that our sample lacks ethnic diversity. Indeed, it would be of value to explore perspectives over time specific to certain ethnic minority groups to reveal potentially unique and important culturally dependent priorities [39,40].

Continued consensus work to refine existing core outcome and core measurement sets is crucial. Modular components of core sets using findings from this study could be developed, whereby different sets of outcomes are considered "core" to address priorities specific to time points and specific to stakeholder groups. The use of applicable modules can accommodate differences in stakeholder priorities and optimise outcome measurement relevant to short, medium and long term priorities. This would reduce research waste and outcome measurement burden. Further research is also needed to better understand impacts of temporal sensitivity of outcomes during the recovery from burn injuries. Additional qualitative work might assess more granular aspects of themes identified in this work amongst more homogenous patient groups. This can include, for instance, exploring more nuanced psychological concepts that are important to specific patient groups (e.g. adolescents or ethnic minority patients) or specific injury patterns (e.g. large burns) over time. Further empirical work is necessary to explore commonly understood time frames for recovery periods (e.g. short, medium, long term) which may facilitate the standardisation of time frames for outcome measurement in trials. This would allow efficient integration of essential and meaningful outcome measures into trial design, avoiding research waste and enabling tailored treatment and intervention development in burns care.

CONCLUSION

The study recommends that important outcomes in burns care are measured at different time points during the recovery from a burn injury. In addition, it is suggested that outcomes are considered for both patients and professionals. Enhanced recovery assessment through the inclusion of priorities not currently considered has the potential to uncover important areas for improvement of outcomes assessment. Further work is needed to elucidate nuances of outcomes for specific

For peer review only

For peer review only - http://bmjopen.bmj.com/site/about/guidelines.xhtml

patient groups and/or specific burn presentations and harmonise time periods across contexts for efficient integration of temporal sensitivity of outcome measurement.

For peer review only

For peer review only - http://bmjopen.bmj.com/site/about/guidelines.xhtml

REFERENCES

1 NHS Digital. HES Data Dictionary: Admitted Patient Care. 2019.

2 NHS England. NHS STANDARD CONTRACT FOR SPECIALISED BURNS CARE (ALL AGES). 2013.

3 Falder S, Browne A, Edgar D, *et al.* Core outcomes for adult burn survivors: A clinical overview. Burns. 2009;**35**:618–41. doi:10.1016/j.burns.2008.09.002

4 T B, S M, M M. If it can't be measured it can't be managed. The paucity of outcome measures in burn care. *Burns* 2003;**29**:757–757. doi:10.1016/J.BURNS.2003.09.006

5 Lemon J, Cooper J, Defres S, *et al.* Understanding parental perspectives on outcomes following paediatric encephalitis: A qualitative study. *PLoS One* 2019;**14**:1–15. doi:10.1371/journal.pone.0220042

6 Mathers J, Moiemen N, Bamford A, *et al.* Ensuring that the outcome domains proposed for use in burns research are relevant to adult burn patients: A systematic review of qualitative research evidence. *Burn Trauma* 2021;**8**:30. doi:10.1093/BURNST/TKAA030

7 Jones LL, Calvert M, Moiemen N, *et al.* Outcomes important to burns patients during scar management and how they compare to the concepts captured in burn-specific patient reported outcome measures. *Burns* 2017;**43**:1682–92. doi:10.1016/j.burns.2017.09.004

8 Spies M, Herndon DN, Rosenblatt JI, *et al.* Prediction of mortality from catastrophic burns in children. *Lancet* 2003;**361**:989–94. doi:10.1016/S0140-6736(03)12824-3

9 Kornhaber RA, De Jong AEE, McLean L. Rigorous, robust and systematic: Qualitative research and its contribution to burn care. An integrative review. *Burns* 2015;**41**:1619–26. doi:10.1016/J.BURNS.2015.04.007

10 Simons M, Price N, Kimble R, *et al.* Patient experiences of burn scars in adults and children and development of a health-related quality of life conceptual model: A qualitative study. In: *Burns*. Elsevier Ltd 2016. 620–32. doi:10.1016/j.burns.2015.11.012

11 MB K, R G, MR E, *et al.* Patients' perspectives on quality of life after burn. *Burns* 2017;**43**:747–56. doi:10.1016/J.BURNS.2016.11.016

12 Young A, Brookes S, Rumsey N, *et al.* Agreement on what to measure in randomised controlled trials in burn care: Study protocol for the development of a core outcome set. *BMJ Open* 2017;**7**:e017267. doi:10.1136/bmjopen-2017-017267

13 Williamson PR, Altman DG, Blazeby JM, *et al.* Developing core outcome sets for clinical trials: Issues to consider. *Trials* 2012;**13**:1–8. doi:10.1186/1745-6215-13-132

14 O'Brien BC, Harris IB, Beckman TJ, *et al.* Standards for reporting qualitative research: A synthesis of recommendations. *Acad Med* 2014;**89**:1245–51. doi:10.1097/ACM.0000000000000388

15 Bunniss S, Kelly DR. Research paradigms in medical education research. *Med Educ* 2010;**44**:358–66. doi:10.1111/J.1365-2923.2009.03611.X

16 Braun V, Clarke V. Using thematic analysis in psychology. *Qual Res Psychol* 2006;**3**:77–101. doi:10.1191/1478088706qp063oa

17 Joffe H, Yardley L. Content and thematic analysis. In: Marks D, Yardley L, eds. *Research methods for clinical and health psychology*. London: : Sage 2004. 56–69.

For peer review only - http://bmjopen.bmj.com/site/about/guidelines.xhtml

18 Edlich RF, Larkham N, O'Hanlan JT, *et al.* Modification of the american burn association injury severity grading system. *J Am Coll Emerg Physicians* 1978;**7**:226–8. doi:10.1016/S0361-1124(78)80381-5

19 Young AE, Davies A, Bland S, *et al.* Systematic review of clinical outcome reporting in randomised controlled trials of burn care. *BMJ Open* 2019;**9**:e025135. doi:10.1136/BMJOPEN-2018-025135

20 Gee Kee EL, Kimble RM, Cuttle L, *et al.* Randomized controlled trial of three burns dressings for partial thickness burns in children. *Burns* 2015;**41**:946–55. doi:10.1016/J.BURNS.2014.11.005

21 Bayuo J, Wong FKY, Chung LYF. Effects of a nurse-led transitional burns rehabilitation programme (4Cs-TBuRP) for adult burn survivors: protocol for a randomised controlled trial. *Trials* 2021;**22**. doi:10.1186/S13063-021-05679-7

22 Zhou Y-P, Jiang Z-M, Sun Y-H, *et al.* The Effect of Supplemental Enteral Glutamine on Plasma Levels, Gut Function, and Outcome in Severe Burns: A Randomized, Double-Blind, Controlled Clinical Trial. *J Parenter Enter Nutr* 2003;**27**. doi:10.1177/0148607103027004241

23 Douglas H, Lynch J, Harms KA, *et al.* Carbon dioxide laser treatment in burn-related scarring: A prospective randomised controlled trial. *J Plast Reconstr Aesthetic Surg* 2019;**72**:863–70. doi:10.1016/J.BJPS.2019.01.027

24 Wolf SE, Edelman LS, Kemalyan N, *et al.* Effects of Oxandrolone on Outcome Measures in the Severely Burned: A Multicenter Prospective Randomized Double-Blind Trial. *J Burn Care Res* 2006;**27**:131–9. doi:10.1097/01.BCR.0000202620.55751.4F

25 Kao Y, Loh EW, Hsu CC, *et al.* Fluid Resuscitation in Patients With Severe Burns: A Meta-analysis of Randomized Controlled Trials. *Acad Emerg Med* 2018;**25**:320–9. doi:10.1111/ACEM.13333

26 Sinha IP, Gallagher R, Williamson PR, *et al.* Development of a core outcome set for clinical trials in childhood asthma: a survey of clinicians, parents, and young people. *Trials 2012 131* 2012;**13**:1–9. doi:10.1186/1745-6215-13-103

27 Ambler GK, Brookes-Howell L, Jones JAR, *et al.* Development of Core Outcome Sets for People Undergoing Major Lower Limb Amputation for Complications of Peripheral Vascular Disease. *Eur J Vasc Endovasc Surg* 2020;**60**:730–8. doi:10.1016/J.EJVS.2020.06.021

28 Ambler GK, Bosanquet DC, Brookes-Howell L, *et al.* Development of a core outcome set for studies involving patients undergoing major lower limb amputation for peripheral arterial disease: study protocol for a systematic review and identification of a core outcome set using a Delphi survey. *Trials 2017 181* 2017;**18**:1–7. doi:10.1186/S13063-017-2358-9

29 Dos Santos F, Drymiotou S, Antequera Martin A, *et al.* Development of a core outcome set for trials on induction of labour: an international multistakeholder Delphi study. *BJOG An Int J Obstet Gynaecol* 2018;**125**:1673–80. doi:10.1111/1471-0528.15397

30 Auerbach SM, Martelli MF, Mercuri LG. Anxiety, information, interpersonal impacts, and adjustment to a stressful health care situation. *J Pers Soc Psychol* 1983;**44**:1284–96. doi:10.1037/0022-3514.44.6.1284

31 Andrew JM. Recovery from surgery, with and without preparatory instruction, for three coping styles. *J Pers Soc Psychol* 1970;**15**:223–6. doi:10.1037/H0029442

32 Gammon J, Mulholland CW. Effect of preparatory information prior to elective total hip

For peer review only

replacement on post-operative physical coping outcomes. *Int J Nurs Stud* 1996;**33**:589–604. doi:10.1016/S0020-7489(96)00019-3

33 Tomsis Y, Perez E, sharabi L, *et al.* Postpartum Post-Traumatic Stress Symptoms Following Cesarean Section—the Mediating Effect of Sense of Control. *Psychiatr Q 2021 924* 2021;**92**:1839–53. doi:10.1007/S11126-021-09949-0

34 Lau-Walker MO, Cowie MR, Roughton M. Coronary heart disease patients' perception of their symptoms and sense of control are associated with their quality of life three years following hospital discharge. *J Clin Nurs* 2009;**18**:63–71. doi:10.1111/J.1365-2702.2008.02386.X

35 Southwick FS, Southwick SM. The Loss of a Sense of Control as a Major Contributor to Physician Burnout: A Neuropsychiatric Pathway to Prevention and Recovery. *JAMA Psychiatry* 2018;**75**:665–6. doi:10.1001/JAMAPSYCHIATRY.2018.0566

36 Ryan CM, Lee A, Kazis LE, *et al.* Recovery trajectories after burn injury in young adults: Does burn size matter? *J Burn Care Res* 2015;**36**:118–29. doi:10.1097/BCR.0000000000000214

37 Pham TN, Kramer CB, Wang J, *et al.* Epidemiology and outcomes of older adults with burn Injury: An analysis of the national burn repository. *J Burn Care Res* 2009;**30**:30–6. doi:10.1097/BCR.0b013e3181921efc

38 Mason ST, Esselman P, Fraser R, *et al.* Return to work after burn injury: A systematic review. J. Burn Care Res. 2012;**33**:101–9. doi:10.1097/BCR.0b013e3182374439

39 Rawlins JM, Khan AA, Shenton AF, *et al.* Burn patterns of Asian ethnic minorities living in West Yorkshire, UK. *Burns* 2006;**32**:97–103. doi:10.1016/j.burns.2005.08.013

40 Tan KT, Prowse PM, Falder S. Ethnic differences in burn mechanism and severity in a UK paediatric population. *Burns* 2012;**38**:551–5. doi:10.1016/j.burns.2011.10.005

For peer review only

For peer review only - http://bmjopen.bmj.com/site/about/guidelines.xhtml

Acknowledgements

We would like to thank the research nurses Karen Coy, Catherine Spry and Anthony Sack for their help with recruitment for this study. We also thank all participants for dedicating their time and views to this study.

Contributors

AEY conceived the project. CH wrote the manuscript with the support of AEY, PD and DE. CH and AEY performed data collection. CH and PD performed data analysis with support of AYE and DE. DE is a qualitative researcher and provided oversight and guidance on methods. AEY and DE edited and critically revised draft manuscripts. AYE provided strategic oversight over the project. All authors have read and approved the publication.

Funding

This research is funded by The Scar Free Foundation, UK. The Scar Free Foundation is the only medical research charity focused on scarring with the mission to achieve scar free healing within a generation. The project was also supported by the National Institute for Health Research (NIHR) Biomedical Research Centre (BRC) at the University Hospitals Bristol and Weston NHS Foundation Trust and the University of Bristol (BRC-1215-20011). AY has received an NIHR Doctoral Research Fellowship (DRF-2016-09-031 NIHR). This paper represents independent research funded by The Scar Free Foundation. The views expressed in this publication are those of the authors and do not necessarily reflect those of the Scar Free Foundation, the UK National Health Service or the NIHR.

Competing interests

All authors declare no competing interests.

Patient consent for publication

As part of the consent process, patient participants agreed to their anonymised quotes being published in scientific journals.

For peer review only - http://bmjopen.bmj.com/site/about/guidelines.xhtml

Ethics approval

A favourable opinion for the project was granted by the South West - Frenchay Research Ethics Committee, reference 17/SW/0025. AY and CH obtained the written informed consent of each participant before conducting the interviews. It was also ensured participants had read the study information sheet sent to them prior to the interview and that they were informed about their rights as participants, that the conversation would remain confidential, and that the transcript would be anonymised.

Provenance and peer review

Not commissioned; externally peer reviewed

Data availability statement

All relevant data is included in the main body or the supplementary file. The raw datasets used and analysed during the current study are available from the corresponding author upon reasonable request.

Figure legends

Figure 1: Overview of patients' and professionals' priorities over time

For peer review only

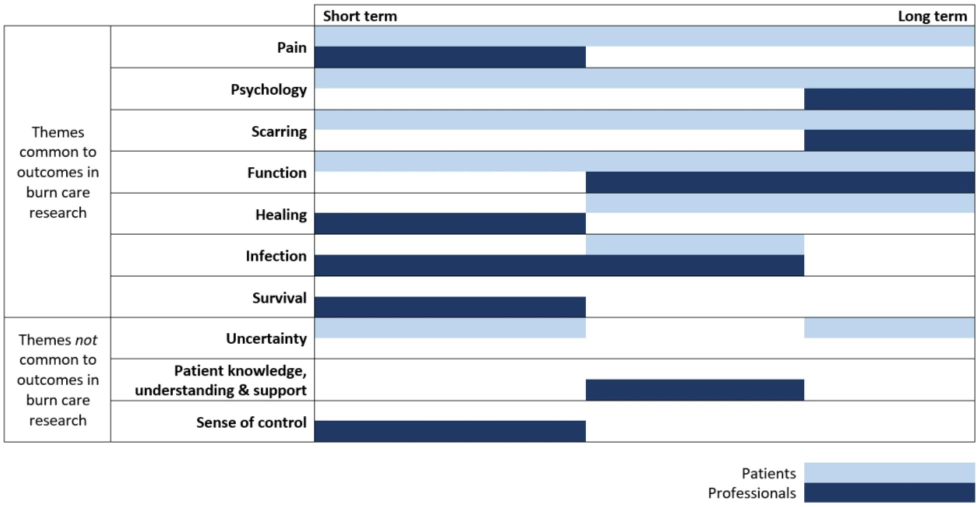

Overview of patients' and professionals' priorities over time

307x156mm (118 x 118 DPI)

Supplemental File - Table S1: Exemplary interview topic guide for patient participants

Topic	Questions
**Intro**	• Introduction to researcher, topic, aims and objectives of the study.<br>• Ask if any questions<br>⇒ *Check that participant is happy to audio record the interview and check consent*
**Background/ warm-up**	• First of all, could you tell me a bit about you?<br>○ *Prompt: do you work, do you have children etc*<br>○ *Prompt for children: how old are you?*<br>• Could you tell me about how you/your child came to have the burn?
**Short-term: Immediately after injury**	• Thinking about the time when you were burned, what were your worries about the immediate effects of the burn at that time?<br>○ *Prompt: e.g. survival, pain*<br>○ *Anything else you can think of? Any other concerns you may have had?*<br>• Still thinking about the time when you were burned, what were your concerns about future effects?<br>○ *Prompt: scarring, movement*<br>*Areas of your life that it was most important to ensure the burn didn't affect eg function (eg related to work, school), the look of the burn (confidence, work, school, friends)? Anything else?*<br>• **What would you say were the most important aspects during that time?**
**Medium-term: After discharge**	• Thinking about the time when you started to recover/got home/were discharged, what were your concerns in relation to your recovery?<br>○ *Was your daily life affected? If so, in what ways? Return to normal.*<br>▪ *Prompts could include work life; social life; family life; relationships (including intimacy)*<br>○ *Were there any issues that affected you that the doctors didn't ask about at your out-patient appointments?*<br>○ *Were there issues that bothered you more than they seemed to bother the medical staff?*<br>○ *Were there issues that the doctors asked about that didn't worry you?*<br>• During the recovery (at home), what were your concerns specifically about the future?<br>○ Prompt: QoL, physical,<br>• Were your family and friends (and if applicable, partner) worried about any effects of your burn at any time after the burn injury? If so, what were they worried about?<br>○ *Prompt: (as above; work life; social life; family life; relationships (including intimate relationships)*<br>○ *Anything else? What other concerns were there?*<br>• **What, in your view, was most important to you during the time after discharge?**
**(Current) Recovery & Future**	• What matters to you (now) while you are getting better?<br>○ *Prompts: similar to above and laddering questions*<br>• And thinking about the future, what are you particularly worried about?<br>• **And again, thinking about your situation now, what is most important to you at the moment in relation to your recovery?**<br>○ Do you think this will matter further down the line?<br>○ If there are any areas of healthcare that could have been improved, what would that/they be?<br>○ *Prompt: treatment of infection, management of pain, improving success of skin-grafting?*
**Wrap-up questions**	• To finish with, could we summarise what the most important areas of your recovery after the injury were to you?<br>⇒ *Wrap up interview*

Standards for Reporting Qualitative Research (SRQR)*

http://www.equator-network.org/reporting-guidelines/srqr/

Title and abstract

	Page/line no(s).
**Title** - Concise description of the nature and topic of the study Identifying the study as qualitative or indicating the approach (e.g., ethnography, grounded theory) or data collection methods (e.g., interview, focus group) is recommended	1
**Abstract** - Summary of key elements of the study using the abstract format of the intended publication; typically includes background, purpose, methods, results, and conclusions	2

Introduction

**Problem formulation** - Description and significance of the problem/phenomenon studied; review of relevant theory and empirical work; problem statement	4
**Purpose or research question** - Purpose of the study and specific objectives or questions	4

Methods

**Qualitative approach and research paradigm** - Qualitative approach (e.g., ethnography, grounded theory, case study, phenomenology, narrative research) and guiding theory if appropriate; identifying the research paradigm (e.g., postpositivist, constructivist/ interpretivist) is also recommended; rationale**	5
**Researcher characteristics and reflexivity** - Researchers' characteristics that may influence the research, including personal attributes, qualifications/experience, relationship with participants, assumptions, and/or presuppositions; potential or actual interaction between researchers' characteristics and the research questions, approach, methods, results, and/or transferability	5-6, 16
**Context** - Setting/site and salient contextual factors; rationale**	2, 5
**Sampling strategy** - How and why research participants, documents, or events were selected; criteria for deciding when no further sampling was necessary (e.g., sampling saturation); rationale**	5
**Ethical issues pertaining to human subjects** - Documentation of approval by an appropriate ethics review board and participant consent, or explanation for lack thereof; other confidentiality and data security issues	5, 20-21
**Data collection methods** - Types of data collected; details of data collection procedures including (as appropriate) start and stop dates of data collection and analysis, iterative process, triangulation of sources/methods, and modification of procedures in response to evolving study findings; rationale**	6

For peer review only - http://bmjopen.bmj.com/site/about/guidelines.xhtml

**Data collection instruments and technologies** - Description of instruments (e.g., interview guides, questionnaires) and devices (e.g., audio recorders) used for data collection; if/how the instrument(s) changed over the course of the study	6
**Units of study** - Number and relevant characteristics of participants, documents, or events included in the study; level of participation (could be reported in results)	7-8
**Data processing** - Methods for processing data prior to and during analysis, including transcription, data entry, data management and security, verification of data integrity, data coding, and anonymization/de-identification of excerpts	6-7
**Data analysis** - Process by which inferences, themes, etc., were identified and developed, including the researchers involved in data analysis; usually references a specific paradigm or approach; rationale**	6-7
**Techniques to enhance trustworthiness** - Techniques to enhance trustworthiness and credibility of data analysis (e.g., member checking, audit trail, triangulation); rationale**	5-7

Results/findings

**Synthesis and interpretation** - Main findings (e.g., interpretations, inferences, and themes); might include development of a theory or model, or integration with prior research or theory	9
**Links to empirical data** - Evidence (e.g., quotes, field notes, text excerpts, photographs) to substantiate analytic findings	9-14

Discussion

**Integration with prior work, implications, transferability, and contribution(s) to the field -** Short summary of main findings; explanation of how findings and conclusions connect to, support, elaborate on, or challenge conclusions of earlier scholarship; discussion of scope of application/generalizability; identification of unique contribution(s) to scholarship in a discipline or field	15-16
**Limitations** - Trustworthiness and limitations of findings	16

Other

**Conflicts of interest** - Potential sources of influence or perceived influence on study conduct and conclusions; how these were managed	20
**Funding** - Sources of funding and other support; role of funders in data collection, interpretation, and reporting	20

*The authors created the SRQR by searching the literature to identify guidelines, reporting standards, and critical appraisal criteria for qualitative research; reviewing the reference lists of retrieved sources; and contacting experts to gain feedback. The SRQR aims to improve the transparency of all aspects of qualitative research by providing clear standards for reporting qualitative research.

For peer review only

**The rationale should briefly discuss the justification for choosing that theory, approach, method, or technique rather than other options available, the assumptions and limitations implicit in those choices, and how those choices influence study conclusions and transferability. As appropriate, the rationale for several items might be discussed together.

Reference:

O'Brien BC, Harris IB, Beckman TJ, Reed DA, Cook DA. **Standards for reporting qualitative research: a synthesis of recommendations.** *Academic Medicine*, Vol. 89, No. 9 / Sept 2014 DOI: 10.1097/ACM.0000000000000388

For peer review only

For peer review only - http://bmjopen.bmj.com/site/about/guidelines.xhtml

BMJ Open

Exploring what is important during burn recovery: A qualitative study investigating priorities of patients and healthcare professionals over time

Journal:	*BMJ Open*
Manuscript ID	bmjopen-2021-059528.R1
Article Type:	Original research
Date Submitted by the Author:	14-Jun-2022
Complete List of Authors:	Hoffmann, Christin; University of Bristol, Population Health Sciences<br>Davies, Philippa ; University of Bristol, Population Health Sciences<br>Elliott, Daisy; University of Bristol, Population Health Sciences<br>Young, Amber; University of Bristol, Population Health Sciences; University Hospitals Bristol and Weston NHS Foundation Trust
**Primary Subject Heading**:	Qualitative research
Secondary Subject Heading:	Patient-centred medicine, Health services research
Keywords:	Quality in health care < HEALTH SERVICES ADMINISTRATION & MANAGEMENT, QUALITATIVE RESEARCH, Clinical trials < THERAPEUTICS

SCHOLARONE™
Manuscripts

I, the Submitting Author has the right to grant and does grant on behalf of all authors of the Work (as defined in the below author licence), an exclusive licence and/or a non-exclusive licence for contributions from authors who are: i) UK Crown employees; ii) where BMJ has agreed a CC-BY licence shall apply, and/or iii) in accordance with the terms applicable for US Federal Government officers or employees acting as part of their official duties; on a worldwide, perpetual, irrevocable, royalty-free basis to BMJ Publishing Group Ltd ("BMJ") its licensees and where the relevant Journal is co-owned by BMJ to the co-owners of the Journal, to publish the Work in this journal and any other BMJ products and to exploit all rights, as set out in our licence.

The Submitting Author accepts and understands that any supply made under these terms is made by BMJ to the Submitting Author unless you are acting as an employee on behalf of your employer or a postgraduate student of an affiliated institution which is paying any applicable article publishing charge ("APC") for Open Access articles. Where the Submitting Author wishes to make the Work available on an Open Access basis (and intends to pay the relevant APC), the terms of reuse of such Open Access shall be governed by a Creative Commons licence – details of these licences and which Creative Commons licence will apply to this Work are set out in our licence referred to above.

Other than as permitted in any relevant BMJ Author's Self Archiving Policies, I confirm this Work has not been accepted for publication elsewhere, is not being considered for publication elsewhere and does not duplicate material already published. I confirm all authors consent to publication of this Work and authorise the granting of this licence.

For peer review only

Exploring what is important during burn recovery: A qualitative study investigating priorities of patients and healthcare professionals over time

Submission category: Original research

Authors:
C Hoffmann[1], c.hoffmann@bristol.ac.uk

P Davies[1], Philippa.Davies@bristol.ac.uk

D Elliott[1], daisy.elliott@bristol.ac.uk

A E Young[1,2], amber.young1@nhs.net

Author affiliations:

[1] National Institute for Health Research Bristol Biomedical Research Centre, Bristol Centre for Surgical Research, Bristol Medical School: Population Health Sciences, University of Bristol, 39 Whatley Road, Clifton, Bristol, BS8 2PS, UK

[2] Bristol Royal Hospital for Children, University Hospitals Bristol and Weston NHS Foundation Trust, Upper Maudlin St, Bristol BS2 8BJ

Correspondence: Christin Hoffmann [c.hoffmann@bristol.ac.uk]

Running title: PRIORITIES OF BURNS PATIENTS AND PROFESSIONALS OVER TIME

Keywords: Burns; Qualitative; Outcomes; Outcome measurement

Word count: 4993 (excluding title page, abstract, tables and references)

For peer review only - http://bmjopen.bmj.com/site/about/guidelines.xhtml

Abstract

Objectives: This qualitative study aimed to investigate: i) priorities of patients and healthcare professionals during recovery from a burn injury, ii) how priorities change over time, and ii) how priorities map to outcomes currently reported in burns research.

Design: Semi-structured interviews were conducted. Interviews were audio recorded, transcribed and analysed thematically.

Setting, participants: A total of 53 patients and healthcare professionals were recruited from four NHS burn services across England and Wales across England and Wales. Patient participants (n=32) included adults, adolescents and parents of paediatric patients, with a variety of burn injuries in terms of severity and cause of burn injury. Healthcare professionals (n=21) were NHS staff members involved in burn care and included professionals with a range of clinical experience and roles (e.g. nurses, surgeons, occupational therapists, physiotherapist, administration).

Results: A total of ten themes relating to priorities during recovery from a burn injury were identified for patients and professionals. Of those, six were identified for patients and professionals ('pain', 'psychological wellbeing', 'healing', 'scarring', 'function', 'infection'), three were unique to professionals ('patient knowledge, understanding & support', 'sense of control', 'survival') and one was unique to patients ('uncertainty'). Results highlighted that importance of these priorities changes over time (e.g. 'survival' was only a concern in the short term). Likewise, priorities differed between patients and professionals (e.g. 'pain' was important to patients throughout their recovery, but not for professionals). Seven out of ten themes overlapped with outcomes commonly assessed in burn research.

Conclusion: Professionals' and patients' priorities (important outcomes) change over time after injury and differ between those groups. Burn care research should take into account the time-sensitivity of routinely measured outcomes to accurately reflect complexity of burn recovery.

(293 words)

For peer review only - http://bmjopen.bmj.com/site/about/guidelines.xhtml

Strengths and limitations of this study

- A diverse sample of professionals and patients were recruited from four NHS burn services across England and Wales to elicit a wide range of perspectives.
- The study employed semi-structured interviews with a large sample of 52 participants
- Multiple layers of analyses were applied, involving thematic analysis, comparison of views between patients and professionals, and mapping themes to outcomes
- Identified themes are broadly relevant to patients with all types of burn injuries, but a more homogeneous sample of burn types may provide more detailed and nuanced insights into priorities of specific groups of patients.

For peer review only

For peer review only - http://bmjopen.bmj.com/site/about/guidelines.xhtml

INTRODUCTION

Each year, approximately 10,000 patients with burn injuries are treated in UK NHS burn services [1]. Burns can affect many aspects of patients' lives, including their function, cosmesis and psychological well-being. Injury management incurs challenges due to their varying complexity (e.g. burn size and location) and factors unique to patients' personal circumstances (e.g. age, profession) [2]. On-going development of treatments to improve outcomes (e.g. quality of life, scarring) means that evidence for optimal strategies for treating burns is still evolving [3]. Accurate measurement of patient relevant outcomes to evaluate strategies is key to achieving high-quality, patient-centred care [3,4].

The importance of relating patients' perceptions to measurable patient-relevant outcomes in burn research has been recognised [5–7]. Burns can impact affected individuals and their families in the short and in the long term in different ways [8]. For larger burns, for example, survival can be the main priority in the acute stages of treatment, whilst scarring may be a long term concern. Similarly, scarring may be more or less important for some population groups and professionals involved their care [9]. It is probable that perceptions about what matters most to patients and professionals may vary between those groups and over time. Consequently, the importance of outcomes may also vary. Current methods in burns research, however, commonly assume equal importance of outcomes at a specific time for all stakeholders [10]. Measuring a certain outcome at a certain time might not, however, provide a true picture of patients' recovery, and decisions about optimal treatment may therefore be ill informed. It is unclear how specific priorities differ across time as well as between patient and professionals as recovery from a burn injury progresses.

There is a need to identify which priorities are important at which time points during burns recovery. This may facilitate optimisation of outcome measurement and ensure evaluation of treatment strategies is patient-centred. Qualitative research is important in burns care [11] and has been widely used to explore perspectives and concepts important to burns survivors [3,6,12]. Previous work has shown that (mostly objective) clinical outcomes thought to be important, may not align with patients' views and perspectives [13]. A large number of qualitative studies have been conducted to explore concepts important to burns survivors only [6]. Likewise, outcome taxonomies have been proposed in this area and include patient-reported and patient-relevant concepts [14,15]. Less attention, however, has been paid to exploring professionals' views and literature often focusses on specific aspects of the recovery, such as scarring characteristics [15]. It is therefore important to understand professionals' *and* patients' priorities during burn recovery as both need to play a role in making decisions about treatment and care [16,17].

For peer review only - http://bmjopen.bmj.com/site/about/guidelines.xhtml

Aim

This qualitative study had three objectives:

i. Investigate priorities of burns patients (including adults, adolescents and parents of paediatric patients) and healthcare professionals during burn recovery and explore how they differ.
ii. Explore how priorities of patients and healthcare professionals change over time.
iii. Map identified themes to outcomes currently reported in burns research.

METHODS

This study uses qualitative methods, employing in-depth, semi-structured interviews to explore participants' perspectives over time.

Conduct and reporting of this study adhered to standards for reporting qualitative research (SRQR) [18]. This study adopted an interpretivist approach to understand participants perspective, because it is acknowledged that experiences of all burn survivors are unique and that individuals' perceptions are construed within their own particular setting which may implicate their families and social networks [19].

Setting and participants

This study recruited two groups of participants (patients and healthcare professionals) from four NHS burn service across England and Wales. Three were adult services and one specialised in paediatric burns. Patient participants were NHS patients with lived experience of burn injuries. These included adults (of 16 years and older), adolescents (10-15 years of age) and parents of paediatric patients (of less than ten years of age) (henceforth simplified to 'patients'). All patients were more than 30 days after burn injury and were not in acute treatment to ensure that participants could be considered within their long term recovery phase and were all able to reflect on their journey up until that point. Healthcare professional participants were multidisciplinary NHS employees of burn services at the selected study sites (henceforth simplified to 'professionals').

Eligible participants were approached by research nurses or clinical / academic researchers (KC, AS, CS, AY) and invited to participate in the study. Information about the study was provided and any questions were answered before written consent was obtained. A clinical (AY) or academic (CH)

For peer review only - http://bmjopen.bmj.com/site/about/guidelines.xhtml

researcher, both trained and experienced in qualitative research, contacted participants to arrange a suitable interview method, location, date and time.

Purposive sampling was employed to select participants with from a wide range of backgrounds [20]. Participant characteristics were reviewed regularly to ensure maximum possible variation between patients (e.g. age, gender, severity and cause of burn injury) and professionals (e.g. clinical experience, role). Recruitment continued until no new ideas and concepts evolved from the interviews (data saturation).

Data collection

Interviews with patients and professionals were completed face-to-face or over the phone, depending on participant preference. Face-to-face interviews were carried out in a quiet and private space in one of the four hospitals to ensure no interruptions or time limitations were impacting the interviews. In-depth interviews were conducted independently by two study members (AY, CH). AY is a consultant anaesthetist in paediatric burn care with 23 years of clinical experience and substantial experience of burns research. CH is a social scientist and qualitative researcher with no involvement in burns care or research prior to this study.

Interviews followed a semi-structured format using a topic guide with open-ended questions to guide discussions (see supplementary files, Table S1 and Table S2 for examples). An initial topic guide was developed based on the study objectives of exploring priorities over time. Participants were asked to retrospectively reflect on their experience at different times after burn injury. To ensure consistency of explored perspectives, time periods were defined *a priori*: a) short term: immediately after the injury, b) medium term: from around the time of discharge and/or wound healing c) long term: during rehabilitation. The topic guide was piloted prior to study commencement but allowed to evolve during the study whereby iterative refinements were made as data collection progressed.

Analysis

All interviews were audio-recorded using an encrypted device, and de-identified during transcription. Random samples of verbatim transcripts were checked for accuracy by one researcher (CH).

For peer review only

Analysis of qualitative data was undertaken using a qualitative data management software (NVivo Version 12). In a first step, transcripts were analysed using thematic analysis [21] to understand priorities of patients and professionals (study objective i). Interview transcripts were analysed separately for patients and professionals to accommodate concepts unique to each group and facilitate later examination of differences. This study used deductive and inductive approaches to coding [22]. Codes for short, medium and long term priorities were developed *a priori*, to explore perspectives over time (study objective ii). *A priori* codes corresponded to previously defined time points (short term, medium term, long term). Additional codes were identified through line-by-line coding following principles of inductive theme development in line with six steps of thematic analysis [21]. Transcripts were read and re-read to allow familiarisation with the data. Initial codes were assigned from which themes were developed: Coded excerpts were grouped into themes of similar meaning and organised into a hierarchical structure of themes and sub-themes. This thematic structure was refined as new themes emerged. These were then reviewed and refined through discussions amongst the study team until final definitions and labels were agreed: This process involved iterative rounds of interpretation of contexts specific to the interviewees' injury and identification of relationships between themes. Diagrams illustrating themes and relationships were used to visualise the data and aid discussions between team members. In a second step, differences in priorities between patients and professionals were examined by comparing narrative accounts of each group. Connections were drawn between patients and professionals by qualitatively contrasting themes identified for each group to find similarities and differences in their perspectives (study objective i). Coding and analyses were undertaken independently by two researchers (CH, PD) who met regularly to discuss emerging themes from the data. Close contact between the coders during data analyses ensured consistency in coding and agreement of interpretation of the data. Independent double-coding was also performed for 25% of transcripts by the two reviewing authors. A purposive sample of transcripts that underwent double-coding was used to ensure maximum variation by participant group, socio-demographic background and injury severity. Data from double-coding were compared and discussed to ensure consistency in approaches to further coding. Any uncertainties and discrepancies during this process were discussed with the wider study team and in consultation with an experienced qualitative researcher who was independent to the study team (DE). Interim interpretation and structure of themes was discussed with the wider study group and if necessary, iteratively modified. Analyses were carried out in parallel to data collection to determine data saturation. This means, no new codes or meaning were identified in the interviews and sufficient data was collected to address the research objectives [23,24]. This process was overseen by the senior author (AY) who has a clinical background in burn care. A final thematic

For peer review only

structure and meaning of themes were agreed in team discussions. Findings were collected in two descriptive accounts (one for patients, one for professionals) to be able to identify similarities and differences in key findings.

Themes identified through analyses described above were mapped against examples of existing outcome domains to assess whether perceptions explored in this work align with routinely measured outcomes in burns research (objective iii). This step will contextualise identified themes within the current literature. A simple search using keywords related to "burns" and keywords for relevant outcomes was conducted in PubMed in November 2021 to identify articles reporting burn trials and relevant systematic review studies. Supplemental hand searching of reference lists of identified articles was performed where applicable. Identified articles were reviewed to compare conceptual content of relevant outcomes reported in studies with themes identified in this work (e.g. by exploring how reported outcomes were defined or measured to ensure that the meaning of the outcome aligns with identified themes). Existing outcome frameworks which comprehensively summarise outcomes in burn care were also cross-referenced [6,14,25]. Search and review of articles ceased when a relevant example was identified. Any data supporting themes identified in this work were tabulated.

Participant characteristics were summarised using descriptive statistics. Data related to burn size were grouped into categories (minor, moderate, major) according to percentage total body surface area (TBSA), following established classification guidelines [26].

Patient and Public Involvement

This study is part of a wider project that aims to standardise outcome reporting and measurement in studies of burns injuries. Patient representatives and carers were actively involved in the design, conduct, analysis and dissemination of aspects of the wider project.

RESULTS

Participant characteristics

A total of 53 participants (32 patient participants, 21 professional participants) took part in semi-structured interviews, lasting between 15 and 45 minutes (mean = 32 minutes). Four patient interviews were conducted with two participants (three with a young person and their parents, one

For peer review only - http://bmjopen.bmj.com/site/about/guidelines.xhtml

included a patient and their carer). Participants were recruited from four NHS trusts between June 2017 and February 2020.

Patient participants' age ranged from 1 – 82 years (*Mean*=38; SD=27.4) and 19 (59%) participants were female. The sample mainly consisted of adults (n=20 participants, 65%). Scalds were the most common burn type (n=10, 36%). Participants primarily suffered from minor burns (n=14, 50%) with six (21%) patients who experienced major burns.

Healthcare professionals sampled were mostly female (n=17, 82%) with the majority of participants in a nursing role (n=11, 52%). Detailed participant characteristics are presented by participant group in Table 1.

Table 1 Participant characteristics (N=53)

	**Patients (N=32)**	**Professionals (N=21)**
**Age, mean (SD; range)**	38 (27.4; 1-82)	
**Female, n (%)**	19 (59)	17 (82)
**Occupation, n (%)**		
Consultant clinical psychologist		3 (14)
Physiotherapist		1 (5)
Consultant, registrar		2 (10)
Occupational therapist		3 (14)
Research, Specialist, Clinic or Senior nurse		11 (52)
Administration		1 (5)
**Patient type, n (%)**		
Adult (16 years and older)	20 (63)	
Young person (10-15 years)	3 (9)	
Parent (of child aged <10 years)	6 (19)	
Parent (of young person aged 10-15)	3 (9)	
**Burn size, n (%)***		
Minor (<10% TBSA)	14 (50)	
Moderate (10-20% TBSA)	2 (7)	
Major (>20% TBSA)	6 (21)	
Not reported	6 (21)	
**Burn reason, n (%)***		
Scald	10 (36)	
Flame	8 (29)	
Chemical	3 (11)	
Flash	3 (11)	
Contact	2(7)	
Electric	1 (4)	
Not reported	1 (4)	

** % data based on number of patient interviews (n=28) because this reflects the number of injuries*

For peer review only - http://bmjopen.bmj.com/site/about/guidelines.xhtml

Priorities of patients and professionals over time (objectives i and ii)

A total of ten themes were identified as relating to participants' priorities during burn recovery. Six themes were most important for patients and professionals ('pain', 'psychological wellbeing', 'healing', 'scarring', 'function', 'infection') and four themes were identified as most important to only patients or professionals (i.e., 'uncertainty', 'patient knowledge, understanding & support', 'sense of control', 'survival'). There were differences in the relative importance of these priorities over time, and differences between patients and professionals. An overview of themes and their importance for patients and professionals at different time points during recovery can be found in Figure 1. Themes are summarised below and presented in order of their prevalence in the data, ascertained by the number of coded references.

Pain (patients and professionals)

Pain was a concern common to patients and professionals in the short term. Pain was a major cause of distress for the majority of patients and their family members as a result of the injury and initial treatment.

Professionals were particularly concerned about pain and adequate pain management to prevent distress and any effects in the long term.

Some patients were worried about pain throughout their recovery. Especially, patients with larger burns, who complained about different aspects of long term pain. For example, itching, scar tightness and contractures causing discomfort, neuropathic pain during nerve regeneration were all mentioned. Professionals, however, did not mention pain as a general concern in the long term.

> *"Managing the pain is the immediate thing." [professional, BCH30]*
>
> *"When it first happens it's just about the pain. That's it. […] that is just all you're focussing on at the time" [patient, NBT012]*
>
> *"So the biggest problem is sometimes, you know, a bit of pain in the leg for example, but it doesn't stop me doing things, you know, like walking but sometimes […] it is perhaps because it's tighter than it should be, I don't know, but it was more painful but its easing.' [patient NBT007]*

Psychological wellbeing (patients and professionals)

Psychological wellbeing and related impacts were concerns common for patients and professionals in the long term. Both groups' concerns were predominantly linked to scar appearance, mentioning

For peer review only - http://bmjopen.bmj.com/site/about/guidelines.xhtml

internal (e.g. self-esteem, acceptance and normalisation of the injury) and external (e.g. bullying, worry related to intimate relationships and other people's views) influences on psychological wellbeing.

Professionals were mainly worried about long lasting impacts on patients' psychological wellbeing, manifesting in post-traumatic stress disorder (PTSD) from distress caused by injury and scarring. In particular, patients with larger burns described psychological impacts during the later phases of recovery (e.g. coping, PTSD).

For patients, the psychological effects of having a burn were apparent during all stages of recovery. In the short term, injuries affected individuals (e.g. worry and anxiety) and family members (e.g. anxiety and guilt, in particular parents). Psychological impacts were also highlighted in the medium term by patients (e.g. problems sleeping, flashbacks).

> *"I mean long-term the important outcomes really are how they are psychologically" [professional, BCH031].*
>
> *"When she saw her face she just broke down. She said 'I'm really ugly mummy'." [patient, BCH001]*
>
> *"I was just getting too upset and depressed over the fact that I had burns and I looked so different and had too many worries and thoughts in my head that I just really didn't want to think about" [patient, CW002]*

<u>*Healing (patients and professionals)*</u>

Healing of the wound was a common concern, expressed frequently by professionals and patients. Importance of healing across the stages of injury recovery, however, differed across participant groups.

Professionals worried about healing in the short term. This was evident through narratives about concerns relating to adequate management of the wound during the acute injury phase, including a quick and smooth process of assessment, cleaning and dressing.

Patients' worry about healing was apparent in the medium to long term of recovery and often linked to feelings of surprise, frustration or worries about the amount of time it was taking for the wound to heal. The appearance of the wound was strongly linked to perceived length of healing times.

> *"Quite often we're very focused on dealing with the physical aspects of the burn injury, so making sure the burn wound heals, preventing infection. [...] we're very focused on managing that wound and [...] making sure the wound heals [...]. [professional, BCH014]*

For peer review only - http://bmjopen.bmj.com/site/about/guidelines.xhtml

For peer review only

"When you see it it's quite gnarly, […] like when its fresh you're like oh god, this is never going to heal" [patient, NBT002]

Scarring (patients and professionals)

Scarring was repeatedly mentioned by both participant groups to be of importance in the long term.

Professionals were mostly concerned about appropriate scar management to optimise their appearance and avoid restrictions on movement.

Similarly, patients' worries in the later phases of recovery spanned any need for further treatment and potential impacts on movement as well as the appearance of scars influencing self-esteem and body image that could affect psychological wellbeing. For patients, the worries about risk of visible scarring were also evident in the short and medium term of the recovery. Those concerns affected individuals and their family members in terms of worry about their appearance and associated future impacts on the patient (e.g. affecting psychological wellbeing).

"Long-term would be the scarring and whether contracture is affecting movement" [professional, BCH013]

"Well the worries were the scarring and whether we'd need to have- at that point in time we didn't know- no-one could tell us how bad it was." [patient, BCH001]

Just wondering what the scarring would do, whether the skin would be very tight. Because you do see people who've had burns, like facial burns and sometimes their skin is very pink and red and then you do notice it. But yes, I think that was about it really. It's just how functional your hands would be when they're recovered. [patient, NBT027]

Function (patients and professionals)

Return to normal functioning was a concern common to patients and professionals in the medium and long term. Participants discussed worries about impacts of the injury (or scar) on daily activities and how it affected patients returning to their normal lives was an important concern following discharge.

Professionals' priorities in relation to function were also focussed on aspects of mobility, primarily ensuring physical movement.

For patients, return to normal function was also a priority in the short term by highlighting concerns about the ability to return to work.

For peer review only - http://bmjopen.bmj.com/site/about/guidelines.xhtml

> *"The earlier you can intervene with physio then that's going to help with movement, […] it's important ongoing as well if the wounds are that bad that there is movement issues." [professional, BCH030]*

> *"So initially I think it's kind of their return to their sort of family roles and their normal activities, when can I go back to work, when can I do my leisure activities." [professional, NBT004]*

> *"I was concerned with my hands to start off with because that is my livelihood." [patient, NBT012]*

> *"Particularly bathroom […], I absolutely hated the bedpans but I was too weak to get out of bed" [patient, CW006]*

<u>*Infection (patients and professionals*</u>

Infection was mentioned by patients and professionals. Importance of infection over time, however, differed across participant groups.

Preventing infection was one of the main priorities for professionals in the short and medium term. Following patients' hospital admission, professionals highlighted the importance of adequate cleaning and preparation of the wound to reduce healing time and scarring. Worry about cleanliness extended to the medium term of burn recovery when professionals wanted to ensure *"that the patient understands how to look after their wounds, and to look out for signs of infection" [professional, NBT026].*

Patients discussed worries about infection related to the medium term of their recovery. Whilst most interviewed patients were aware of the need to keep the wound clean, infection was discussed as major concern primarily by those patients who had experienced symptoms of infection.

> *"Just to make sure there's no infection. If there's a lot of dead skin hanging around or blisters aren't de-roofed you could potentially get an infection just in the fluid that's still around or the dead skin." [professional, BCH001]*

> *"I thought that I had been released for good and was at home and actually I was extremely unwell. Night sweats, worrying about all sorts, just want, you know, really just completely in the dark about what was going to happen and I think because there's so much forewarning that it can happen, you know, then the worst does. It's a very upsetting thing to go through." [patient, CW004]*

<u>*Patient knowledge, understanding & support (professionals only)*</u>

This theme was identified as a unique priority for professionals. In the medium term, at the point of discharge, professionals felt it was crucial to ensure patients are *"well equipped with the information*

For peer review only - http://bmjopen.bmj.com/site/about/guidelines.xhtml

that they need" [professional, BCH006]. This encompassed information related to support the self-management of the wound (e.g. creaming, massaging, sun protection). Professionals' main objectives were also to help patients understand their injury (e.g. recognise signs of infection) and gain self-confidence in further wound treatment, so they remain as independent from the service as possible. Interviewees also repeatedly mentioned the importance of communicating the constant support available to patients which included readiness to answer any questions and psychological support.

> *"It's explaining to them what they need to be doing to achieve this wound to heal" [professional, NBT026]]*

> *"My biggest goal and concern for them is can they access everything they want to access." [professional, BCH007]*

Uncertainty (patients only)

Uncertainty was identified as a unique theme for patients and was an important concern during the earlier stages of recovery. Feelings of uncertainty related to how the injury happened, the treatments needed and their possible outcomes as well as the likely course of recovery. Participants associated feelings of anxiety with uncertainty but also described how information provision (e.g. about recovery and further treatment) can lessen those feelings. For patients with more severe burns, uncertainties remained in the long-term and were mostly related to scar management and future treatment plans.

> *"During the time that we were kind of up in the air as to whether he would have the operation or not, we saw loads of different people on different rotas and stuff, and I kind of felt like there were lots of different opinions flying around about how well it was healing, how well it wasn't healing, what the next course of treatment would be. And although I understand why that was the case, because in the main they were kind of waiting to see what the wound was going to do, we felt very up in the air about […] what was going to happen, and whether what happened eventually would have actually been the right thing." [patient, BCH017]*

> *"Yeah, you've got no information, no knowledge and the pain materialises, you don't know whether that's supposed to happen, was that not supposed to happen, is something going wrong." [patient, NBT012]*

Sense of control (professionals only)

For a smaller number of professionals, the sense of feeling in control of the treatment process was identified as a priority in the short term. This included major concerns about being in control of the

For peer review only - http://bmjopen.bmj.com/site/about/guidelines.xhtml

treatment situation (e.g. managing distress well) and technical steps involved. Professionals described the importance of feeling well prepared for the patients' treatment and the presence of a well-functioning team to support a smooth treatment. Consequence of lack of control were mentioned and included impacts on self-confidence and patients' confidence in the care.

> *"It's being in control of that procedure. I feel as a clinical professional, […] once you've lost the control of the procedure in some way then you've lost the parents' confidence in you." [professional, BCH006]*

Survival (professionals only)

Some professionals mentioned survival to be the key priority in the short term and was only discussed in the context of larger burns. For those professionals involved in their care, mortality-related outcomes and physiological signs (e.g. organ failure, fever) were a major concern. Only a minority of patients experienced life-threatening injuries, but those with major burns generally did not discuss survival. This may be caused by *"a kind of hole in their memory and they don't remember what happened to them" [professional, MH006]*, aggravated by severe pain relief and reduced levels of consciousness.

> *"Well you'd be looking at their physical signs and outcomes, things like their vital signs, their bloods, whether they're being ventilated and they're on inotropes, and also whether they're having runs of sepsis and whether they've gone into acute renal failure" [professional, NBT003]*

> *"The most important outcome is survival because there are those patients that are very poorly and you just want them to survive". [professional, BCH007]*

Mapping themes to outcomes (objective iii)

A total of seven out of 10 themes (70%) were identified as recognised outcomes in burn research. These were evident in a recently published systematic review that investigated all outcomes reported in burn care research [14] and identified research studies (see Table 2). An overview of identified exemplary studies assessing an outcome for which conceptual content aligns with themes identified in this study is shown below.

Table 2 Example studies that report outcome and that aligns with present themes

For peer review only - http://bmjopen.bmj.com/site/about/guidelines.xhtml

Theme identified	Outcome reported and verbatim quotes from exemplary burn care research to demonstrate how conceptual content aligns with theme identified
Pain	Outcome reported: Pain and distress<br>Conceptual content reported: "Pain and distress were assessed by obtaining: (1) the participant's self-report of pain intensity using the Faces Pain Scale-Revised (FPS-R) (if participant was aged 3 years or over); (2) the nurse's observational rating of the participant's pain and distress using the face, legs, activity, cry, consolability (FLACC) scale; (3) the participant's self-report (if aged over 8 years) or the parent's report of the participant's pain intensity using a Visual Analog Scale-Pain (VAS-P); (4) pulse rate; and (5) respiratory rate of the participant, taken immediately prior to and after dressing changes.)" [27]
Psychological wellbeing	Outcome reported: Psychological functioning<br>Conceptual content: "The Chinese version of the Hospital Anxiety and Depression Scale (HADS) will be used to evaluate psychological functioning (anxiety and depression). The 14-item *HADS* was designed as a tool to screen for anxiety and depressive symptoms in medical settings and has well-demonstrated solid psychometric properties for use in medical settings and commonly used in burn care settings to screen anxiety and depression" [28]
Healing	Outcome reported: Wound healing<br>Conceptual content: "Wound healing at day 30 was assessed" [29]
Scarring	Outcome reported: Burns scar appearance<br>Conceptual content: "Outcome measures were recorded at baseline, 6-weeks post final treatment and up to 3 years post-treatment. Measures included blinded assessor VSS, Patient Scar Assessment Scale and histological tissue analysis." [30]
Function	Outcome reported: Physical role functioning/performance<br>Conceptual content: "The Chinese version of the Disability of the Arm, Shoulder and Hand Symptom Scale (DASH) will be used to evaluate this outcome. The DASH tool is a 30-item questionnaire that asks the patient to grade symptoms and physical function during the preceding week on a five-point Likert scale." [28]
Infection	Outcome reported: Wound infection<br>Conceptual content: "Investigators also were required to submit documentation of complications their patients encountered during treatment." [31]
Survival	Outcome reported: Mortality<br>Conceptual content: "Mortality referred to death from any reason" [32]

There were three themes (30%) to which no outcome reporting in the literature could be found. 'Uncertainty', 'patient knowledge, understanding & support' and 'sense of control' were all themes uniquely identified in this study [14].

DISCUSSION

This study used semi-structured interviews to investigate perspectives of 53 burn patients, and professionals involved in their care, to explore what is most important as the patients recover. The

For peer review only - http://bmjopen.bmj.com/site/about/guidelines.xhtml

study identified a total of ten themes across both participant groups that related to priorities during recovery from a burn injury. Of those, six were identified across both participant groups ('pain', 'psychological wellbeing', 'healing', 'scarring', 'function' and 'infection'), three were unique to professionals ('patient knowledge, understanding & support', 'sense of control', 'survival') and one was unique to patients ('uncertainty'). The study results also showed that the importance of recovery priorities changes over time (e.g. 'survival' was a concern in the short term but not in the medium to long term), and that priorities differed between patients and professionals (e.g. 'pain' was a concern in the short term for professionals but important to many patients throughout their recovery). Finally, this work established that seven identified themes overlapped with outcomes commonly reported in burn research, whereas three themes could not be mapped to reported outcomes ('uncertainty', 'patient knowledge, understanding & support', 'sense of control').

This work identified unique differences between what patients and professionals view as most important during burn recovery and supports the temporal sensitivity of priorities of patients and professionals. We were unable to find existing empirical evidence from burn care research that shows similar findings. This work, however, does complement other recent efforts to improve patient-centred outcome measurement in burn care research [3,6,7,12] by highlighting the need to align outcome assessment with the changing importance of priorities for different participant groups, and with the change in relevance of priorities at different times after injury or intervention. Research in other healthcare areas has started to acknowledge these complexities of outcome assessment during recovery. Development of a core outcome set for childhood asthma, for example, reported the differences in perspectives of patients and professionals as to which outcomes are most important [33]. Other core outcome sets for lower limb amputation or induction of labour, for example, included separate outcomes relevant to short and medium term recovery [34–36]. This study supports a recommendation for core outcome sets in burns care to differentiate between different time points [3], but current core outcome sets do not yet incorporate these complexities [16].

An aim of on-going research is to ensure that frequently measured outcomes adequately capture all important priorities related to burns recovery [6]. The results of this study highlighted the importance of three themes not aligned with commonly measured outcomes in burn care research. Subjective perceptions related to 'uncertainty' (patients), 'sense of control' and 'patient knowledge, understanding & support' (professionals) were all identified as important priorities at varying time points during recovery. The existence and impacts of uncertainty on patients are well known [37] and, as supported by our findings, and have also been linked to the level of information provision [38,39]. Conversely, professionals in our study highlighted that 'patient knowledge, understanding &

For peer review only - http://bmjopen.bmj.com/site/about/guidelines.xhtml

support' is one of their key priorities during the medium term of patients' recovery from a burn injury. Routine assessment of perceptions relevant to level of uncertainty and information provision as well as assessing patients' level of knowledge and understanding and support received can be important beyond commonly used measures. In addition, these measures of subjective perception might provide useful care quality indicators to highlight where improvements, e.g. in information provision, might be required. Measurement instruments assessing patients' sense of control in healthcare interventions exist [40,41]. Very few studies, however, report the need to feel in control from the perspectives of healthcare professionals [42], and we were unable to find a measure assessing professional specific sense of control. In our study, only a small number of professionals mentioned this as a priority which warrants further research investigating the importance and impacts of perceived sense of control.

Strengths and limitations

Our findings integrated views from a large sample of patients and healthcare professionals (n=53) from various backgrounds, allowing investigation of a variety of perspectives. This means that the findings are broadly representative of commonly occurring burn injuries but simultaneously may not account for unique concerns of specific patient groups. For instance, where burn injuries were related to mental health issues (e.g. self-inflicted injuries), patients may display different patterns of priorities. Similarly, analysis focused on the most important and most commonly raised concerns which implies that individual experiences and priorities may differ to themes identified in this study. Other limitations should be acknowledged. First, there is no consensus on the definition of time frames in burn recovery. This study defined time frames (short, medium and long term) *a priori* which were agreed after expert consultation and discussions within the multi-disciplinary study team. There are, however, challenges in standardising time frames for recovery from burn injury considering the variabilities and complexities of injuries [43–45]. For instance, patients with larger burns experience longer healing [44]. This means that individual definitions of short, medium and long term recovery are highly personal and might depend on contextual complexities (e.g. size and location of burn, time since injury, socio-demographic details of patients, any wound complications), and might therefore differ to the definitions used in this study. Second, our recruitment strategy meant that some patient groups may be underrepresented (e.g. patients with larger burns, adolescent patients). A small number of qualitative studies showed that different population groups experience recovery differently. For example, women were better able to adapt to living with a burn injury [46] and older adults require more support and information [47] following severe burn injury.

For peer review only - http://bmjopen.bmj.com/site/about/guidelines.xhtml

Future research could explore priorities in more homogeneous patient groups with larger sample size, to investigate whether these change between different populations and type of injury. Likewise, professional participants were mainly female and had nursing roles and there were challenges in recruiting surgeons, mainly due to individuals' time constraints. It is unclear if, for example, perspectives of male and female professionals differ and whether a more gender balanced sample would have impacted the results. There is value in exploring this difference in future work. It is also acknowledged that our sample lacks ethnic diversity. Again, it would be of value to explore perspectives over time specific to certain ethnic minority groups to reveal potentially unique and important culturally dependent priorities [48,49].

Continued consensus work to refine existing core outcome and core measurement sets is crucial. Modular components of core sets using findings from this study could be developed, whereby different sets of outcomes are considered "core" to address priorities specific to time points and specific to different stakeholder groups. The use of applicable modules can accommodate differences in stakeholder priorities and optimise outcome measurement relevant to short, medium and long term priorities. This would reduce research waste and outcome measurement burden, but requires effective strategies for implementation in practice [50]. Further research is also needed to better understand impacts of temporal sensitivity of outcomes during the recovery from burn injuries. This work provided a starting point to understanding the variability of the most important and broad priorities of patients and professionals during burn recovery but does not provide an outcome categorisation. Additional qualitative work might assess more granular aspects of themes identified in this work amongst more homogenous patient groups by drawing on existing outcome taxonomies [6,7,14,25]. This can include, for instance, exploring more nuanced psychological concepts that are important to specific patient groups (e.g. adolescents or ethnic minority patients) or specific injury patterns (e.g. large burns) over time. Likewise, priorities important across all stages of recovery require further detailed examination to be able to inform specific outcome measurement. For example, different concepts related to pain (scar pain, burn wound pain, pain during the procedure) might be relevant at different time points. Further empirical work is necessary to explore commonly understood time frames for recovery periods (e.g. short, medium, long term) which may facilitate the standardisation of time frames for outcome measurement in trials. This would allow efficient integration of essential and meaningful outcome measures into trial design, avoiding research waste and enabling tailored treatment and intervention development in burns care.

For peer review only

For peer review only - http://bmjopen.bmj.com/site/about/guidelines.xhtml

CONCLUSION

The study recommends that important outcomes in burns care are measured at different time points during the recovery from a burn injury. In addition, it is suggested that outcomes are considered for both patients and professionals. Enhanced recovery assessment through the inclusion of priorities not currently considered has the potential to uncover important areas for improvement of outcomes assessment. This may ultimately benefit burn victims by effectiveness and improved patient-centred care. Further work is needed to elucidate nuances of outcomes for specific patient groups and/or specific burn presentations and harmonise time periods across contexts for efficient integration of temporal sensitivity of outcome measurement.

For peer review only

For peer review only - http://bmjopen.bmj.com/site/about/guidelines.xhtml

REFERENCES

1 NHS Digital. HES Data Dictionary: Admitted Patient Care. 2019.

2 NHS England. NHS STANDARD CONTRACT FOR SPECIALISED BURNS CARE (ALL AGES). 2013. https://www.england.nhs.uk/wp-content/uploads/2014/04/d06-spec-burn-care-0414.pdf (accessed 5 Jun 2021).

3 Falder S, Browne A, Edgar D, *et al.* Core outcomes for adult burn survivors: A clinical overview. Burns. 2009;**35**:618–41. doi:10.1016/j.burns.2008.09.002

4 Brown T, Mills S, Muller M. If it can't be measured it can't be managed: The paucity of outcome measures in burn care. Burns. 2003;**29**:757. doi:10.1016/j.burns.2003.09.006

5 Lemon J, Cooper J, Defres S, *et al.* Understanding parental perspectives on outcomes following paediatric encephalitis: A qualitative study. *PLoS One* 2019;**14**:1–15. doi:10.1371/journal.pone.0220042

6 Mathers J, Moiemen N, Bamford A, *et al.* Ensuring that the outcome domains proposed for use in burns research are relevant to adult burn patients: A systematic review of qualitative research evidence. *Burn Trauma* 2021;**8**:30. doi:10.1093/BURNST/TKAA030

7 Jones LL, Calvert M, Moiemen N, *et al.* Outcomes important to burns patients during scar management and how they compare to the concepts captured in burn-specific patient reported outcome measures. *Burns* 2017;**43**:1682–92. doi:10.1016/j.burns.2017.09.004

8 Mc Kittrick A, Gustafsson L, Hodson T, *et al.* Exploration of individuals perspectives of recovery following severe hand burn injuries. *Burns* Published Online First: 29 April 2022. doi:10.1016/J.BURNS.2022.04.026

9 Robert R, Meyer W, Bishop S, *et al.* Disfiguring burn scars and adolescent self-esteem. *Burns* 1999;**25**:581–5. doi:10.1016/S0305-4179(99)00065-0

10 Mathers J. Towards the Holistic Assessment of Scar Management Interventions. *Eur Burn J 2022, Vol 3, Pages 207-210* 2022;**3**:207–10. doi:10.3390/EBJ3010018

11 Kornhaber RA, De Jong AEE, McLean L. Rigorous, robust and systematic: Qualitative research and its contribution to burn care. An integrative review. *Burns* 2015;**41**:1619–26. doi:10.1016/J.BURNS.2015.04.007

12 Simons M, Price N, Kimble R, *et al.* Patient experiences of burn scars in adults and children and development of a health-related quality of life conceptual model: A qualitative study. In: *Burns*. Elsevier Ltd 2016. 620–32. doi:10.1016/j.burns.2015.11.012

13 Kool MB, Geenen R, Egberts MR, *et al.* Patients' perspectives on quality of life after burn. *Burns* 2017;**43**:747–56. doi:10.1016/j.burns.2016.11.016

14 Young AE, Davies A, Bland S, *et al.* Systematic review of clinical outcome reporting in randomised controlled trials of burn care. *BMJ Open* 2019;**9**:e025135. doi:10.1136/BMJOPEN-2018-025135

15 Simons M, Lim PCC, Kimble RM, *et al.* Towards a clinical and empirical definition of burn scarring: A template analysis using qualitative data. *Burns* 2018;**44**:1811–9. doi:10.1016/J.BURNS.2018.04.006

16 Young A, Brookes S, Rumsey N, *et al.* Agreement on what to measure in randomised controlled trials in burn care: Study protocol for the development of a core outcome set. *BMJ*

For peer review only - http://bmjopen.bmj.com/site/about/guidelines.xhtml

Open 2017;**7**:e017267. doi:10.1136/bmjopen-2017-017267

17 Williamson PR, Altman DG, Blazeby JM, *et al.* Developing core outcome sets for clinical trials: Issues to consider. *Trials* 2012;**13**:1–8. doi:10.1186/1745-6215-13-132

18 O'Brien BC, Harris IB, Beckman TJ, *et al.* Standards for reporting qualitative research: A synthesis of recommendations. *Acad Med* 2014;**89**:1245–51. doi:10.1097/ACM.0000000000000388

19 Bunniss S, Kelly DR. Research paradigms in medical education research. *Med Educ* 2010;**44**:358–66. doi:10.1111/J.1365-2923.2009.03611.X

20 Patton MQ. *Qualitative research & evaluation methods : integrating theory and practice*. SAGE Publications 2002.

21 Braun V, Clarke V. Using thematic analysis in psychology. *Qual Res Psychol* 2006;**3**:77–101. doi:10.1191/1478088706qp063oa

22 Joffe H, Yardley L. Content and thematic analysis. In: Marks D, Yardley L, eds. *Research methods for clinical and health psychology*. London: : Sage 2004. 56–69.

23 Fusch PI, Ness LR. Are We There Yet? Data Saturation in Qualitative Research. *Qual Rep* 2015;**20**:1408–16.http://www.nova.edu/ssss/QR/QR20/9/fusch1.pdf (accessed 12 May 2022).

24 Hennink MM, Kaiser BN, Marconi VC. Code Saturation Versus Meaning Saturation: How Many Interviews Are Enough? *Qual Health Res* 2017;**27**:591–608. doi:10.1177/1049732316665344

25 Dodd S, Clarke M, Becker L, *et al.* A taxonomy has been developed for outcomes in medical research to help improve knowledge discovery. *J Clin Epidemiol* 2018;**96**:84–92. doi:10.1016/j.jclinepi.2017.12.020

26 Edlich RF, Larkham N, O'Hanlan JT, *et al.* Modification of the american burn association injury severity grading system. *J Am Coll Emerg Physicians* 1978;**7**:226–8. doi:10.1016/S0361-1124(78)80381-5

27 Gee Kee EL, Kimble RM, Cuttle L, *et al.* Randomized controlled trial of three burns dressings for partial thickness burns in children. *Burns* 2015;**41**:946–55. doi:10.1016/J.BURNS.2014.11.005

28 Bayuo J, Wong FKY, Chung LYF. Effects of a nurse-led transitional burns rehabilitation programme (4Cs-TBuRP) for adult burn survivors: protocol for a randomised controlled trial. *Trials* 2021;**22**. doi:10.1186/S13063-021-05679-7

29 Zhou Y-P, Jiang Z-M, Sun Y-H, *et al.* The Effect of Supplemental Enteral Glutamine on Plasma Levels, Gut Function, and Outcome in Severe Burns: A Randomized, Double-Blind, Controlled Clinical Trial. *J Parenter Enter Nutr* 2003;**27**. doi:10.1177/0148607103027004241

30 Douglas H, Lynch J, Harms KA, *et al.* Carbon dioxide laser treatment in burn-related scarring: A prospective randomised controlled trial. *J Plast Reconstr Aesthetic Surg* 2019;**72**:863–70. doi:10.1016/J.BJPS.2019.01.027

31 Wolf SE, Edelman LS, Kemalyan N, *et al.* Effects of Oxandrolone on Outcome Measures in the Severely Burned: A Multicenter Prospective Randomized Double-Blind Trial. *J Burn Care Res* 2006;**27**:131–9. doi:10.1097/01.BCR.0000202620.55751.4F

32 Kao Y, Loh EW, Hsu CC, *et al.* Fluid Resuscitation in Patients With Severe Burns: A Meta-analysis of Randomized Controlled Trials. *Acad Emerg Med* 2018;**25**:320–9.

For peer review only - http://bmjopen.bmj.com/site/about/guidelines.xhtml

doi:10.1111/ACEM.13333

33 Sinha IP, Gallagher R, Williamson PR, *et al.* Development of a core outcome set for clinical trials in childhood asthma: a survey of clinicians, parents, and young people. *Trials 2012 131* 2012;**13**:1–9. doi:10.1186/1745-6215-13-103

34 Ambler GK, Brookes-Howell L, Jones JAR, *et al.* Development of Core Outcome Sets for People Undergoing Major Lower Limb Amputation for Complications of Peripheral Vascular Disease. *Eur J Vasc Endovasc Surg* 2020;**60**:730–8. doi:10.1016/J.EJVS.2020.06.021

35 Ambler GK, Bosanquet DC, Brookes-Howell L, *et al.* Development of a core outcome set for studies involving patients undergoing major lower limb amputation for peripheral arterial disease: study protocol for a systematic review and identification of a core outcome set using a Delphi survey. *Trials 2017 181* 2017;**18**:1–7. doi:10.1186/S13063-017-2358-9

36 Dos Santos F, Drymiotou S, Antequera Martin A, *et al.* Development of a core outcome set for trials on induction of labour: an international multistakeholder Delphi study. *BJOG An Int J Obstet Gynaecol* 2018;**125**:1673–80. doi:10.1111/1471-0528.15397

37 Auerbach SM, Martelli MF, Mercuri LG. Anxiety, information, interpersonal impacts, and adjustment to a stressful health care situation. *J Pers Soc Psychol* 1983;**44**:1284–96. doi:10.1037/0022-3514.44.6.1284

38 Andrew JM. Recovery from surgery, with and without preparatory instruction, for three coping styles. *J Pers Soc Psychol* 1970;**15**:223–6. doi:10.1037/H0029442

39 Gammon J, Mulholland CW. Effect of preparatory information prior to elective total hip replacement on post-operative physical coping outcomes. *Int J Nurs Stud* 1996;**33**:589–604. doi:10.1016/S0020-7489(96)00019-3

40 Tomsis Y, Perez E, sharabi L, *et al.* Postpartum Post-Traumatic Stress Symptoms Following Cesarean Section—the Mediating Effect of Sense of Control. *Psychiatr Q 2021 924* 2021;**92**:1839–53. doi:10.1007/S11126-021-09949-0

41 Lau-Walker MO, Cowie MR, Roughton M. Coronary heart disease patients' perception of their symptoms and sense of control are associated with their quality of life three years following hospital discharge. *J Clin Nurs* 2009;**18**:63–71. doi:10.1111/J.1365-2702.2008.02386.X

42 Southwick FS, Southwick SM. The Loss of a Sense of Control as a Major Contributor to Physician Burnout: A Neuropsychiatric Pathway to Prevention and Recovery. *JAMA Psychiatry* 2018;**75**:665–6. doi:10.1001/JAMAPSYCHIATRY.2018.0566

43 Ryan CM, Lee A, Kazis LE, *et al.* Recovery trajectories after burn injury in young adults: Does burn size matter? *J Burn Care Res* 2015;**36**:118–29. doi:10.1097/BCR.0000000000000214

44 Pham TN, Kramer CB, Wang J, *et al.* Epidemiology and outcomes of older adults with burn Injury: An analysis of the national burn repository. *J Burn Care Res* 2009;**30**:30–6. doi:10.1097/BCR.0b013e3181921efc

45 Mason ST, Esselman P, Fraser R, *et al.* Return to work after burn injury: A systematic review. J. Burn Care Res. 2012;**33**:101–9. doi:10.1097/BCR.0b013e3182374439

46 Williams NR, Davey M, Klock-Powell K. Rising from the Ashes. *http://dx.doi.org/101300/J010v36n04_04* 2008;**36**:53–77. doi:10.1300/J010V36N04_04

47 Jones BA, Buchanan H, Harcourt D. The experiences of older adults living with an appearance altering burn injury: An exploratory qualitative study. *J Health Psychol* 2017;**22**:364–74. doi:10.1177/1359105315603473

For peer review only - http://bmjopen.bmj.com/site/about/guidelines.xhtml

For peer review only

48 Rawlins JM, Khan AA, Shenton AF, *et al.* Burn patterns of Asian ethnic minorities living in West Yorkshire, UK. *Burns* 2006;**32**:97–103. doi:10.1016/j.burns.2005.08.013

49 Tan KT, Prowse PM, Falder S. Ethnic differences in burn mechanism and severity in a UK paediatric population. *Burns* 2012;**38**:551–5. doi:10.1016/j.burns.2011.10.005

50 Hughes KL, Clarke M, Williamson PR. A systematic review finds Core Outcome Set uptake varies widely across different areas of health. *J Clin Epidemiol* 2021;**129**:114–23. doi:10.1016/J.JCLINEPI.2020.09.029

For peer review only

For peer review only - http://bmjopen.bmj.com/site/about/guidelines.xhtml

Acknowledgements

We would like to thank the research nurses Karen Coy, Catherine Spry and Anthony Sack for their help with recruitment for this study. We also thank all participants for dedicating their time and views to this study.

Contributors

AEY conceived the project. CH wrote the manuscript with the support of AEY, PD and DE. CH and AEY performed data collection. CH and PD performed data analysis with support of AYE and DE. DE is a qualitative researcher and provided oversight and guidance on methods. AEY and DE edited and critically revised draft manuscripts. AYE provided strategic oversight over the project. All authors have read and approved the publication.

Funding

This research is funded by The Scar Free Foundation, UK. The Scar Free Foundation is the only medical research charity focused on scarring with the mission to achieve scar free healing within a generation. The project was also supported by the National Institute for Health Research (NIHR) Biomedical Research Centre (BRC) at the University Hospitals Bristol and Weston NHS Foundation Trust and the University of Bristol (BRC-1215-20011). AY has received an NIHR Doctoral Research Fellowship (DRF-2016-09-031 NIHR). This paper represents independent research funded by The Scar Free Foundation. The views expressed in this publication are those of the authors and do not necessarily reflect those of the Scar Free Foundation, the UK National Health Service or the NIHR.

Competing interests

All authors declare no competing interests.

Patient consent for publication

As part of the consent process, patient participants agreed to their anonymised quotes being published in scientific journals.

For peer review only - http://bmjopen.bmj.com/site/about/guidelines.xhtml

Ethics approval

A favourable opinion for the project was granted by the South West - Frenchay Research Ethics Committee, reference 17/SW/0025. AY and CH obtained the written informed consent of each participant before conducting the interviews. It was also ensured participants had read the study information sheet sent to them prior to the interview and that they were informed about their rights as participants, that the conversation would remain confidential, and that the transcript would be anonymised.

Provenance and peer review

Not commissioned; externally peer reviewed

Data availability statement

All relevant data is included in the main body or the supplementary file. The raw datasets used and analysed during the current study are available from the corresponding author upon reasonable request.

Figure legends

Figure 1: Overview of patients' and professionals' priorities over time

For peer review only

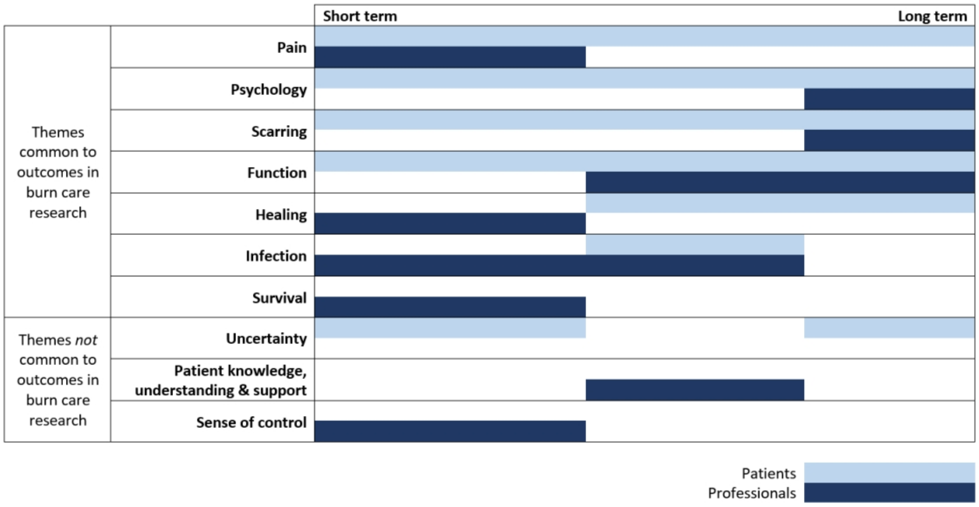

Overview of patients' and professionals' priorities over time

307x156mm (118 x 118 DPI)

Supplemental File - Table S1: Exemplary interview topic guide for patient participants

Topic	Questions
**Intro**	• Introduction to researcher, topic, aims and objectives of the study.<br>• Ask if any questions<br>⇒ *Check that participant is happy to audio record the interview and check consent*
**Background/ warm-up**	• First of all, could you tell me a bit about you?<br>o *Prompt: do you work, do you have children etc*<br>o *Prompt for children: how old are you?*<br>• Could you tell me about how you/your child came to have the burn?
**Short-term: Immediately after injury**	• Thinking about the time when you were burned, what were your worries about the immediate effects of the burn at that time?<br>o *Prompt: e.g. survival, pain*<br>o *Anything else you can think of? Any other concerns you may have had?*<br>• Still thinking about the time when you were burned, what were your concerns about future effects?<br>o *Prompt: scarring, movement*<br>*Areas of your life that it was most important to ensure the burn didn't affect eg function (eg related to work, school), the look of the burn (confidence, work, school, friends)? Anything else?*<br>• **What would you say were the most important aspects during that time?**
**Medium-term: After discharge**	• Thinking about the time when you started to recover/got home/were discharged, what were your concerns in relation to your recovery?<br>o *Was your daily life affected? If so, in what ways? Return to normal.*<br>▪ *Prompts could include work life; social life; family life; relationships (including intimacy)*<br>o *Were there any issues that affected you that the doctors didn't ask about at your out-patient appointments?*<br>o *Were there issues that bothered you more than they seemed to bother the medical staff?*<br>o *Were there issues that the doctors asked about that didn't worry you?*<br>• During the recovery (at home), what were your concerns specifically about the future?<br>o Prompt: QoL, physical,<br>• Were your family and friends (and if applicable, partner) worried about any effects of your burn at any time after the burn injury? If so, what were they worried about?<br>o *Prompt: (as above; work life; social life; family life; relationships (including intimate relationships)*<br>o *Anything else? What other concerns were there?*<br>• **What, in your view, was most important to you during the time after discharge?**
**(Current) Recovery & Future**	• What matters to you (now) while you are getting better?<br>o *Prompts: similar to above and laddering questions*<br>• And thinking about the future, what are you particularly worried about?<br>• **And again, thinking about your situation now, what is most important to you at the moment in relation to your recovery?**<br>o Do you think this will matter further down the line?<br>o If there are any areas of healthcare that could have been improved, what would that/they be?<br>o *Prompt: treatment of infection, management of pain, improving success of skin-grafting?*
**Wrap-up questions**	• To finish with, could we summarise what the most important areas of your recovery after the injury were to you?<br>⇒ *Wrap up interview*

For peer review only

Supplemental File - Table S2: Exemplary interview topic guide for professional participants

Topic	Questions
**Intro**	• Introduction to researcher, topic, aims and objectives of the study.<br>• Ask if any questions<br>⇒ *Check that participant is happy to audio record the interview and check consent*
**Background/ warm-up**	• First of all, could you tell me a bit about you and your role as a healthcare professional?<br>○ *Prompt: how long have you been at the hospital for, what are your responsibilities?*<br>○ *What is your involvement with burns injuries? How long working in burns?*<br>• What are your experiences been with burns patients?<br>• *Which part of the recovery journey are you involved in? When/how regular do you see them?*
**Short-term: Immediately after injury**	• Going back to the very beginning, when patients are admitted to the hospital, what are the relevant outcomes from a clinical perspective?<br>○ E.g. survival, scaring, pain?<br>• **What is the most important outcome?**<br>• What do you think patients are worried about the most during that time?<br>• **What do you think is the most important outcome for patients at that time?**
**Medium-term: After discharge**	• Moving on to when the patient gets discharged for recovery at home, what are relevant outcomes for you from a clinical viewpoint?<br>• **And what was the most important one taking into consideration the recent discharge?**<br>• And again, what do you think the patients are concerned about when they get discharged from hospital?<br>○ *Prompt: daily life, QoL, work, social,*<br>• **What do you think is most important to the patients at the time they are being discharged?**<br>• How do you think do the patients views differ from their families and friends? What are they worries about?<br>• *Prompt: (as above; work life; social life; family life; relationships (including intimate relationships)*
**(Current) Recovery & Future**	• Thinking about burns injury outcomes further down the line, what is important for you in the long run? What is most important?<br>○ What do you think is the most important to patients when thinking about the really long-term effects in the future?
**Wrap-up questions**	• To finish with, could we summarise what the most important areas of patient recovery after the injury?<br>⇒ *Thanks to interviewee*

For peer review only

Standards for Reporting Qualitative Research (SRQR)*

http://www.equator-network.org/reporting-guidelines/srqr/

Title and abstract

	Page/line no(s).
**Title** - Concise description of the nature and topic of the study Identifying the study as qualitative or indicating the approach (e.g., ethnography, grounded theory) or data collection methods (e.g., interview, focus group) is recommended	1
**Abstract** - Summary of key elements of the study using the abstract format of the intended publication; typically includes background, purpose, methods, results, and conclusions	2

Introduction

**Problem formulation** - Description and significance of the problem/phenomenon studied; review of relevant theory and empirical work; problem statement	4
**Purpose or research question** - Purpose of the study and specific objectives or questions	4

Methods

**Qualitative approach and research paradigm** - Qualitative approach (e.g., ethnography, grounded theory, case study, phenomenology, narrative research) and guiding theory if appropriate; identifying the research paradigm (e.g., postpositivist, constructivist/ interpretivist) is also recommended; rationale**	5
**Researcher characteristics and reflexivity** - Researchers' characteristics that may influence the research, including personal attributes, qualifications/experience, relationship with participants, assumptions, and/or presuppositions; potential or actual interaction between researchers' characteristics and the research questions, approach, methods, results, and/or transferability	5-6, 16
**Context** - Setting/site and salient contextual factors; rationale**	2, 5
**Sampling strategy** - How and why research participants, documents, or events were selected; criteria for deciding when no further sampling was necessary (e.g., sampling saturation); rationale**	5
**Ethical issues pertaining to human subjects** - Documentation of approval by an appropriate ethics review board and participant consent, or explanation for lack thereof; other confidentiality and data security issues	5, 20-21
**Data collection methods** - Types of data collected; details of data collection procedures including (as appropriate) start and stop dates of data collection and analysis, iterative process, triangulation of sources/methods, and modification of procedures in response to evolving study findings; rationale**	6

For peer review only

**Data collection instruments and technologies** - Description of instruments (e.g., interview guides, questionnaires) and devices (e.g., audio recorders) used for data collection; if/how the instrument(s) changed over the course of the study	6
**Units of study** - Number and relevant characteristics of participants, documents, or events included in the study; level of participation (could be reported in results)	7-8
**Data processing** - Methods for processing data prior to and during analysis, including transcription, data entry, data management and security, verification of data integrity, data coding, and anonymization/de-identification of excerpts	6-7
**Data analysis** - Process by which inferences, themes, etc., were identified and developed, including the researchers involved in data analysis; usually references a specific paradigm or approach; rationale**	6-7
**Techniques to enhance trustworthiness** - Techniques to enhance trustworthiness and credibility of data analysis (e.g., member checking, audit trail, triangulation); rationale**	5-7

Results/findings

**Synthesis and interpretation** - Main findings (e.g., interpretations, inferences, and themes); might include development of a theory or model, or integration with prior research or theory	9
**Links to empirical data** - Evidence (e.g., quotes, field notes, text excerpts, photographs) to substantiate analytic findings	9-14

Discussion

**Integration with prior work, implications, transferability, and contribution(s) to the field** - Short summary of main findings; explanation of how findings and conclusions connect to, support, elaborate on, or challenge conclusions of earlier scholarship; discussion of scope of application/generalizability; identification of unique contribution(s) to scholarship in a discipline or field	15-16
**Limitations** - Trustworthiness and limitations of findings	16

Other

**Conflicts of interest** - Potential sources of influence or perceived influence on study conduct and conclusions; how these were managed	20
**Funding** - Sources of funding and other support; role of funders in data collection, interpretation, and reporting	20

*The authors created the SRQR by searching the literature to identify guidelines, reporting standards, and critical appraisal criteria for qualitative research; reviewing the reference lists of retrieved sources; and contacting experts to gain feedback. The SRQR aims to improve the transparency of all aspects of qualitative research by providing clear standards for reporting qualitative research.

For peer review only - http://bmjopen.bmj.com/site/about/guidelines.xhtml

**The rationale should briefly discuss the justification for choosing that theory, approach, method, or technique rather than other options available, the assumptions and limitations implicit in those choices, and how those choices influence study conclusions and transferability. As appropriate, the rationale for several items might be discussed together.

Reference:

O'Brien BC, Harris IB, Beckman TJ, Reed DA, Cook DA. **Standards for reporting qualitative research: a synthesis of recommendations.** *Academic Medicine*, Vol. 89, No. 9 / Sept 2014 DOI: 10.1097/ACM.0000000000000388

For peer review only

For peer review only - http://bmjopen.bmj.com/site/about/guidelines.xhtml

BMJ Open

Exploring what is important during burn recovery: A qualitative study investigating priorities of patients and healthcare professionals over time

Journal:	*BMJ Open*
Manuscript ID	bmjopen-2021-059528.R2
Article Type:	Original research
Date Submitted by the Author:	20-Oct-2022
Complete List of Authors:	Hoffmann, Christin; University of Bristol, Population Health Sciences<br>Davies, Philippa ; University of Bristol, Population Health Sciences<br>Elliott, Daisy; University of Bristol, Population Health Sciences<br>Young, Amber; University of Bristol, Population Health Sciences; University Hospitals Bristol and Weston NHS Foundation Trust
**Primary Subject Heading**:	Qualitative research
Secondary Subject Heading:	Patient-centred medicine, Health services research
Keywords:	Quality in health care < HEALTH SERVICES ADMINISTRATION & MANAGEMENT, QUALITATIVE RESEARCH, Clinical trials < THERAPEUTICS

SCHOLARONE™
Manuscripts

I, the Submitting Author has the right to grant and does grant on behalf of all authors of the Work (as defined in the below author licence), an exclusive licence and/or a non-exclusive licence for contributions from authors who are: i) UK Crown employees; ii) where BMJ has agreed a CC-BY licence shall apply, and/or iii) in accordance with the terms applicable for US Federal Government officers or employees acting as part of their official duties; on a worldwide, perpetual, irrevocable, royalty-free basis to BMJ Publishing Group Ltd ("BMJ") its licensees and where the relevant Journal is co-owned by BMJ to the co-owners of the Journal, to publish the Work in this journal and any other BMJ products and to exploit all rights, as set out in our licence.

The Submitting Author accepts and understands that any supply made under these terms is made by BMJ to the Submitting Author unless you are acting as an employee on behalf of your employer or a postgraduate student of an affiliated institution which is paying any applicable article publishing charge ("APC") for Open Access articles. Where the Submitting Author wishes to make the Work available on an Open Access basis (and intends to pay the relevant APC), the terms of reuse of such Open Access shall be governed by a Creative Commons licence – details of these licences and which Creative Commons licence will apply to this Work are set out in our licence referred to above.

Other than as permitted in any relevant BMJ Author's Self Archiving Policies, I confirm this Work has not been accepted for publication elsewhere, is not being considered for publication elsewhere and does not duplicate material already published. I confirm all authors consent to publication of this Work and authorise the granting of this licence.

For peer review only

Exploring what is important during burn recovery: A qualitative study investigating priorities of patients and healthcare professionals over time

Submission category: Original research

Authors:
C Hoffmann[1], c.hoffmann@bristol.ac.uk

P Davies[1], Philippa.Davies@bristol.ac.uk

D Elliott[1], daisy.elliott@bristol.ac.uk

A E Young[1,2,*]

Author affiliations:

[1] National Institute for Health Research Bristol Biomedical Research Centre, Bristol Centre for Surgical Research, Bristol Medical School: Population Health Sciences, University of Bristol, 39 Whatley Road, Clifton, Bristol, BS8 2PS, UK

[2] Bristol Royal Hospital for Children, University Hospitals Bristol and Weston NHS Foundation Trust, Upper Maudlin St, Bristol BS2 8BJ

*The author sadly passed away on 17 September 2022. The paper was revised and resubmitted for publication by the corresponding author.

Correspondence: Christin Hoffmann [c.hoffmann@bristol.ac.uk]

Running title: PRIORITIES OF BURNS PATIENTS AND PROFESSIONALS OVER TIME

Keywords: Burns; Qualitative; Outcomes; Outcome measurement

Word count: 4993 (excluding title page, abstract, tables and references)

For peer review only - http://bmjopen.bmj.com/site/about/guidelines.xhtml

Abstract

Objectives: This qualitative study aimed to investigate: i) priorities of patients and healthcare professionals during recovery from a burn injury, ii) how priorities change over time, and ii) how priorities map to outcomes currently reported in burns research.

Design: Semi-structured interviews were conducted. Interviews were audio recorded, transcribed and analysed thematically.

Setting, participants: A total of 53 patients and healthcare professionals were recruited from four National Health Service (NHS) burn services across England and Wales across England and Wales. Patient participants (n=32) included adults, adolescents, and parents of paediatric patients, with a variety of burn injuries in terms of severity and cause of burn injury. Healthcare professionals (n=21) were NHS staff members involved in burn care and included professionals with a range of clinical experience and roles (e.g. nurses, surgeons, occupational therapists, physiotherapist, administration).

Results: Ten themes relating to priorities during recovery from a burn injury were identified for patients and professionals. Of those, six were identified for patients and professionals ('pain and discomfort', 'psychological wellbeing', 'healing', 'scarring', 'function', 'infection'), three were unique to professionals ('patient knowledge, understanding & support', 'sense of control', 'survival') and one was unique to patients ('uncertainty'). Results highlighted that importance of these priorities changes over time (e.g. 'survival' was only a concern in the short term). Likewise, priorities differed between patients and professionals (e.g. 'pain' was important to patients throughout their recovery, but not for professionals). Seven out of ten themes overlapped with outcomes commonly assessed in burn research.

Conclusion: Professionals' and patients' priorities (important outcomes) change over time after injury and differ between those groups. Burn care research should take into account the time-sensitivity of routinely measured outcomes to accurately reflect complexity of burn recovery.

(293 words)

For peer review only - http://bmjopen.bmj.com/site/about/guidelines.xhtml

Strengths and limitations of this study

- A diverse sample of professionals and patients were recruited from four NHS burn services across England and Wales to elicit a wide range of perspectives.
- The study employed semi-structured interviews with a large sample of 52 participants
- Multiple layers of analyses were applied, involving thematic analysis, comparison of views between patients and professionals, and mapping themes to outcomes
- Identified themes are broadly relevant to patients with all types of burn injuries, but a more homogeneous sample of burn types may provide more detailed and nuanced insights into priorities of specific groups of patients.

For peer review only

INTRODUCTION

Each year, approximately 10,000 patients with burn injuries are treated in the United Kingdom National Health Service (NHS) burn services [1]. Burns can affect many aspects of patients' lives, including their function, cosmesis and psychological well-being. Injury management incurs challenges due to their varying complexity (e.g. burn size and location) and factors unique to patients' personal circumstances (e.g. age, profession) [2]. On-going development of treatments to improve outcomes (e.g. quality of life, scarring) means that evidence for optimal strategies for treating burns is still evolving [3]. Accurate measurement of patient relevant outcomes to evaluate strategies is key to achieving high-quality, patient-centred care [3,4].

The importance of relating patients' perceptions to measurable patient-relevant outcomes in burn research has been recognised [5–7]. Burns can impact affected individuals and their families in the short and in the long term in different ways [8]. For larger burns, for example, survival can be the main priority in the acute stages of treatment, whilst scarring may be a long term concern. Similarly, scarring may be more or less important for some population groups and professionals involved their care [9]. It is probable that perceptions about what matters most to patients and professionals may vary between those groups and over time. Consequently, the importance of outcomes may also vary. Current methods in burns research, however, commonly assume equal importance of outcomes at a specific time for all stakeholders [10]. Measuring a certain outcome at a certain time might not, however, provide a true picture of patients' recovery, and decisions about optimal treatment may therefore be ill informed. It is unclear how specific priorities differ across time as well as between patient and professionals as recovery from a burn injury progresses.

There is a need to identify which priorities are important at which time points during burns recovery. This may facilitate optimisation of outcome measurement and ensure evaluation of treatment strategies is patient-centred. Qualitative research is important in burns care [11] and has been widely used to explore perspectives and concepts important to burns survivors [3,6,12]. Previous work has shown that (mostly objective) clinical outcomes thought to be important, may not align with patients' views and perspectives [13]. A large number of qualitative studies have been conducted to explore concepts important to burns survivors only [6]. Likewise, outcome taxonomies have been proposed in this area and include patient-reported and patient-relevant concepts [14,15]. Less attention, however, has been paid to exploring professionals' views and literature often focusses on specific aspects of the recovery, such as scarring characteristics [15]. It is therefore important to understand professionals' *and* patients' priorities during burn recovery as both need to

For peer review only - http://bmjopen.bmj.com/site/about/guidelines.xhtml

play a role in making decisions about treatment and care [16,17]. Likewise, understanding how they are linked to commonly measured outcomes in burn care research may guide future study design.

Aim

This qualitative study had three objectives:

i. Investigate priorities of burns patients (including adults, adolescents and parents of paediatric patients) and healthcare professionals during burn recovery and explore how they differ.
ii. Explore how priorities of patients and healthcare professionals change over time.
iii. Map identified themes to outcomes currently reported in burns research.

METHODS

This study uses qualitative methods, employing in-depth, semi-structured interviews to explore participants' perspectives over time.

Conduct and reporting of this study adhered to standards for reporting qualitative research (SRQR) [18]. This study adopted an interpretivist approach to understand participants perspective, because it is acknowledged that experiences of all burn survivors are unique and that individuals' perceptions are construed within their own particular setting which may implicate their families and social networks [19].

Setting and participants

This study recruited two groups of participants (patients and healthcare professionals) from four NHS burn service across England and Wales. Three were adult services and one specialised in paediatric burns. Patient participants were NHS patients with lived experience of burn injuries. These included adults (of 16 years and older), adolescents (10-15 years of age) and parents of paediatric patients (of less than ten years of age) (henceforth simplified to 'patients'). Patients with varying injury severity and burn sizes were eligible. Interviews were conducted at least 30 days after injury and/or with patients who were not in acute treatment (e.g. larger burns). This ensured that all participants were able to reflect on their experiences around the time when the injury happened and during acute treatment, and that they were also able to articulate what is most important to them when in the following stages of recovery. Healthcare professional participants were

For peer review only - http://bmjopen.bmj.com/site/about/guidelines.xhtml

multidisciplinary NHS employees of burn services at the selected study sites (henceforth simplified to 'professionals').

Eligible participants were approached by research nurses or clinical / academic researchers (KC, AS, CS, AY) and invited to participate in the study. Information about the study was provided and any questions were answered before written consent was obtained. A clinical (AY) or academic (CH) researcher, both trained and experienced in qualitative research, contacted participants to arrange a suitable interview method, location, date and time.

Purposive sampling was employed to select participants with from a wide range of backgrounds [20]. Participant characteristics were reviewed regularly to ensure maximum possible variation between patients (e.g. age, gender, severity and cause of burn injury) and professionals (e.g. clinical experience, role). Recruitment continued until no new ideas and concepts evolved from the interviews (see "analysis" section for further detail on our approach for data saturation).

Data collection

Interviews with patients and professionals were completed face-to-face or over the phone, depending on participant preference. Face-to-face interviews were carried out in a quiet and private space in one of the four hospitals to ensure no interruptions or time limitations were impacting the interviews. In-depth interviews were conducted independently by two study members (AY, CH). AY is a consultant anaesthetist in paediatric burn care with 23 years of clinical experience and substantial experience of burns research. CH is a social scientist and qualitative researcher with no involvement in burns care or research prior to this study.

Interviews followed a semi-structured format using a topic guide with open-ended questions to guide discussions (see supplementary files, Table S1 and Table S2 for examples). Participants were asked to retrospectively reflect on their experience or priorities relating to different times after burn injury. There are no consensus definitions of time frames in burn care. To ensure consistency of explored perspectives, significant points during recovery from a burn injury were used as temporal anchors (e.g. point of discharge is considered a crucial point for outcome assessment [21]). For the purpose of this study, time periods were therefore defined *a priori* as: short term (immediately after the injury), medium term (from around the time of discharge and/or wound healing) and long term (during rehabilitation). An initial topic guide was developed based on the study objectives of exploring priorities retrospectively over time. This was piloted prior to study commencement but

For peer review only - http://bmjopen.bmj.com/site/about/guidelines.xhtml

allowed to evolve during the study whereby iterative refinements were made as data collection progressed.

Analysis

All interviews were audio-recorded using an encrypted device, and de-identified during transcription. Random samples of verbatim transcripts were checked for accuracy by one researcher (CH).

Analysis of qualitative data was undertaken using a qualitative data management software (NVivo Version 12). In a first step, transcripts were analysed using thematic analysis [22] to understand priorities of patients and professionals (study objective i). Interview transcripts were analysed separately for patients and professionals to accommodate concepts unique to each group and facilitate later examination of differences. This study used deductive and inductive approaches to coding [23]. Codes for short, medium and long term priorities were developed *a priori*, to explore perspectives over time (study objective ii). *A priori* codes corresponded to time points (short term, medium term, long term) defined for the purpose of this study (described above). Additional codes were identified through line-by-line coding following principles of inductive theme development in line with six steps of thematic analysis [22]. Transcripts were read and re-read to allow familiarisation with the data. Initial codes were assigned from which themes were developed: Coded excerpts were grouped into themes of similar meaning and organised into a hierarchical structure of themes and sub-themes. This thematic structure was refined as new themes emerged. These were then reviewed and refined through discussions amongst the study team until final definitions and labels were agreed: This process involved iterative rounds of interpretation of contexts specific to the interviewees' injury and identification of relationships between themes. Diagrams illustrating themes and relationships were used to visualise the data and aid discussions between team members. In a second step, differences in priorities between patients and professionals were examined by comparing narrative accounts of each group. Connections were drawn between patients and professionals by qualitatively contrasting themes identified for each group to find similarities and differences in their perspectives (study objective i). This step also involved combining themes by condensing and relabelling which was shaped by the data and the team's experience and knowledge with defining, identifying and classifying outcomes in burns research.

Coding and analyses were undertaken independently by two researchers (CH, PD) who met regularly to discuss emerging themes from the data. Close contact between the coders during data analyses ensured consistency in coding and agreement of interpretation of the data. Independent double-

For peer review only - http://bmjopen.bmj.com/site/about/guidelines.xhtml

coding was also performed for 25% of transcripts by the two reviewing authors. A purposive sample of transcripts that underwent double-coding was used to ensure maximum variation by participant group, socio-demographic background and injury severity. Data from double-coding were compared and discussed to ensure consistency in approaches to further coding. Any uncertainties and discrepancies during this process were discussed with the wider study team and in consultation with an experienced qualitative researcher who was independent to the study team (DE). Interim interpretation and structure of themes was discussed with the wider study group and if necessary, iteratively modified.

Analyses were carried out in parallel to data collection to determine data saturation separately for both participant groups. This means, no new codes or meaning were identified through additional interviews and sufficient data was collected to address the research objectives [24–27]. Specifically, interim results during analyses were reviewed regularly by the study team and decisions of whether saturation was achieved were based on impressions of whether collected data provides sufficient conceptual depth [28,29]. If saturation was expected, a small number of additional interviews were conducted to confirm that no new codes or meaning were identified [30]. This process was overseen by the senior author (AY) who has a clinical background in burn care. A final thematic structure and meaning of themes were agreed in team discussions. Findings were collected in two descriptive accounts (one for patients, one for professionals) to be able to identify similarities and differences in key findings.

Themes identified through analyses described above were mapped against common outcomes in trials to assess whether perceptions explored in this work align with routinely measured outcomes in burns research (objective iii). This step involved comparing identified themes and their conceptual content to an existing outcome classification (see supplementary file, Table S3) which was developed through synthesis of 955 unique outcomes reported across 147 randomised controlled trials in burn care [14].

This multi-layer analysis allowed to elicit priorities qualitatively from experiences of patients and professionals while linking experiential data to previously identified and classified outcomes.

Participant characteristics were summarised using descriptive statistics. Data related to burn size were grouped into categories (minor, moderate, major) according to percentage total body surface area (TBSA), following established classification guidelines [31].

For peer review only - http://bmjopen.bmj.com/site/about/guidelines.xhtml

Patient and Public Involvement

This study is part of a wider project that aims to standardise outcome reporting and measurement in studies of burns injuries. Patient representatives and carers were actively involved in the design, conduct, analysis and dissemination of aspects of the wider project.

RESULTS

Participant characteristics

A total of 53 participants (32 patient participants, 21 professional participants) took part in semi-structured interviews, lasting between 15 and 45 minutes (mean = 32 minutes). Four patient interviews were conducted with two participants (three with a young person and their parents, one included a patient and their carer). Participants were recruited from four NHS trusts between June 2017 and February 2020.

Patient participants' age ranged from 1 – 82 years (*Mean*=38; SD=27.4) and 19 (59%) participants were female. The sample mainly consisted of adults (n=20 participants, 65%). Scalds were the most common burn type (n=10, 36%). Participants primarily suffered from minor burns (n=14, 50%) with six (21%) patients who experienced major burns.

Healthcare professionals sampled were mostly female (n=17, 82%) with the majority of participants in a nursing role (n=11, 52%). Detailed participant characteristics are presented by participant group in Table 1.

Table 1 Participant characteristics (N=53)

	**Patients (N=32)**	**Professionals (N=21)**
**Age, mean (SD; range)**	38 (27.4; 1-82)	
**Female, n (%)**	19 (59)	17 (82)
**Occupation, n (%)**		
Consultant clinical psychologist		3 (14)
Physiotherapist		1 (5)
Consultant, registrar		2 (10)
Occupational therapist		3 (14)
Research, Specialist, Clinic or Senior nurse		11 (52)
Administration		1 (5)
**Patient type, n (%)**		
Adult (16 years and older)	20 (63)	
Young person (10-15 years)	3 (9)	

For peer review only - http://bmjopen.bmj.com/site/about/guidelines.xhtml

Parent (of child aged <10 years)	6 (19)
Parent (of young person aged 10-15)	3 (9)
**Burn size, n (%)***	
Minor (<10% TBSA)	14 (50)
Moderate (10-20% TBSA)	2 (7)
Major (>20% TBSA)	6 (21)
Not reported	6 (21)
**Burn reason, n (%)***	
Scald	10 (36)
Flame	8 (29)
Chemical	3 (11)
Flash	3 (11)
Contact	2(7)
Electric	1 (4)
Not reported	1 (4)

** % data based on number of patient interviews (n=28) because this reflects the number of injuries*

Priorities of patients and professionals over time (objectives i and ii)

A total of ten combined themes were identified as relating to participants' priorities during burn recovery. Six themes were most important for patients and professionals ('pain and discomfort', 'psychological wellbeing', 'healing', 'scarring', 'function', 'infection') and four themes were identified as most important to only patients or professionals ('uncertainty', 'patient knowledge, understanding & support', 'sense of control', 'survival'). There were differences in the relative importance of these priorities over time, and differences between patients and professionals. An overview of combined themes and their importance for patients and professionals at different time points during recovery can be found in Figure 1. A detailed account of all themes identified for each stakeholder group and how each is linked to a combined theme and outcome domains can be found in supplementary file – descriptive accounts, and Tables S4 and S5. The combined themes are summarised below and presented in order of their prevalence in the data, ascertained by the number of coded references.

Pain and discomfort (patients and professionals)

Pain was a concern common to patients and professionals in the short and medium term. Pain was a major cause of distress for the majority of patients and their family members as a result of the injury and initial treatment.

Professionals were particularly concerned about pain and adequate pain management immediately after the injury to prevent distress affecting patients in the long term. Reducing discomfort during

For peer review only - http://bmjopen.bmj.com/site/about/guidelines.xhtml

wound healing, such as easing itching and skin tightness were also raised as important in the medium term.

Some patients were worried about pain throughout their recovery. Especially, patients with larger burns, who complained about different aspects of long term pain. For example, itching, scar tightness and contractures causing discomfort, neuropathic pain during nerve regeneration were all mentioned. Professionals, however, did not mention pain as a general concern in the long term.

> *"Managing the pain is the immediate thing." [professional, BCH30]*
>
> *"When it first happens it's just about the pain. That's it. […] that is just all you're focussing on at the time" [patient, NBT012]*
>
> *"So the biggest problem is sometimes, you know, a bit of pain in the leg for example, but it doesn't stop me doing things, you know, like walking but sometimes […] it is perhaps because it's tighter than it should be, I don't know, but it was more painful but its easing.' [patient NBT007]*

<u>*Psychological wellbeing (patients and professionals)*</u>

Psychological wellbeing and related impacts were concerns common for patients and professionals in the long term. Both groups' concerns were predominantly linked to scar appearance, mentioning internal (e.g. self-esteem, acceptance and normalisation of the injury) and external (e.g. bullying, worry related to intimate relationships and other people's views) influences on psychological wellbeing.

Professionals were mainly worried about long lasting impacts on patients' psychological wellbeing, manifesting in post-traumatic stress disorder (PTSD) from distress caused by injury and scarring. In particular, patients with larger burns described psychological impacts during the later phases of recovery (e.g. coping, PTSD).

For patients, the psychological effects of having a burn were apparent during all stages of recovery. In the short term, injuries affected individuals (e.g. worry and anxiety) and family members (e.g. anxiety and guilt, in particular parents). Psychological impacts were also highlighted in the medium term by patients (e.g. problems sleeping, flashbacks).

> *"I mean long-term the important outcomes really are how they are psychologically" [professional, BCH031].*
>
> *"When she saw her face she just broke down. She said 'I'm really ugly mummy'." [patient, BCH001]*

For peer review only

"I was just getting too upset and depressed over the fact that I had burns and I looked so different and had too many worries and thoughts in my head that I just really didn't want to think about" [patient, CW002]

Healing (patients and professionals)

Healing of the wound was a common concern, expressed frequently by professionals and patients. Importance of healing across the stages of injury recovery, however, differed across participant groups.

Professionals worried about healing in the short term. This was evident through narratives about concerns relating to adequate management of the wound during the acute injury phase, including a quick and smooth process of assessment, cleaning and dressing.

Patients' worry about healing was apparent in the medium to long term of recovery and often linked to feelings of surprise, frustration or worries about the amount of time it was taking for the wound to heal. The appearance of the wound was strongly linked to perceived length of healing times.

"Quite often we're very focused on dealing with the physical aspects of the burn injury, so making sure the burn wound heals, preventing infection. […] we're very focused on managing that wound and […] making sure the wound heals […]. [professional, BCH014]

"When you see it it's quite gnarly, […] like when its fresh you're like oh god, this is never going to heal" [patient, NBT002]

Scarring (patients and professionals)

Scarring was repeatedly mentioned by both participant groups to be of importance in the long term.

Professionals were mostly concerned about appropriate scar management to optimise their appearance and avoid restrictions on movement.

Similarly, patients' worries in the later phases of recovery spanned any need for further treatment and potential impacts on movement as well as the appearance of scars influencing self-esteem and body image that could affect psychological wellbeing. For patients, the worries about risk of visible scarring were also evident in the short and medium term of the recovery. Those concerns affected individuals and their family members in terms of worry about their appearance and associated future impacts on the patient (e.g. affecting psychological wellbeing).

"Long-term would be the scarring and whether contracture is affecting movement" [professional, BCH013]

For peer review only - http://bmjopen.bmj.com/site/about/guidelines.xhtml

> *"Well the worries were the scarring and whether we'd need to have- at that point in time we didn't know- no-one could tell us how bad it was." [patient, BCH001]*
>
> *Just wondering what the scarring would do, whether the skin would be very tight. Because you do see people who've had burns, like facial burns and sometimes their skin is very pink and red and then you do notice it. But yes, I think that was about it really. It's just how functional your hands would be when they're recovered. [patient, NBT027]*

Function (patients and professionals)

Return to normal functioning was a concern common to patients and professionals in the medium and long term. Participants discussed worries about impacts of the injury (or scar) on daily activities and how it affected patients returning to their normal lives was an important concern following discharge.

Professionals' priorities in relation to function were also focussed on aspects of mobility, primarily ensuring physical movement.

For patients, return to normal function was also a priority in the short term by highlighting concerns about the ability to return to work.

> *"The earlier you can intervene with physio then that's going to help with movement, […] it's important ongoing as well if the wounds are that bad that there is movement issues." [professional, BCH030]*
>
> *"So initially I think it's kind of their return to their sort of family roles and their normal activities, when can I go back to work, when can I do my leisure activities." [professional, NBT004]*
>
> *"I was concerned with my hands to start off with because that is my livelihood." [patient, NBT012]*
>
> *"Particularly bathroom […], I absolutely hated the bedpans but I was too weak to get out of bed" [patient, CW006]*

Infection (patients and professionals)

Infection was mentioned by patients and professionals. Importance of infection over time, however, differed across participant groups.

Preventing infection was one of the main priorities for professionals in the short and medium term. Following patients' hospital admission, professionals highlighted the importance of adequate cleaning and preparation of the wound to reduce healing time and scarring. Worry about cleanliness extended to the medium term of burn recovery when professionals wanted to ensure *"that the*

For peer review only - http://bmjopen.bmj.com/site/about/guidelines.xhtml

patient understands how to look after their wounds, and to look out for signs of infection" [professional, NBT026].

Patients discussed worries about infection related to the medium term of their recovery. Whilst most interviewed patients were aware of the need to keep the wound clean, infection was discussed as major concern primarily by those patients who had experienced symptoms of infection.

> *"Just to make sure there's no infection. If there's a lot of dead skin hanging around or blisters aren't de-roofed you could potentially get an infection just in the fluid that's still around or the dead skin." [professional, BCH001]*

> *"I thought that I had been released for good and was at home and actually I was extremely unwell. Night sweats, worrying about all sorts, just want, you know, really just completely in the dark about what was going to happen and I think because there's so much forewarning that it can happen, you know, then the worst does. It's a very upsetting thing to go through." [patient, CW004]*

Patient knowledge, understanding & support (professionals only)

This theme was identified as a unique priority for professionals. In the medium term, at the point of discharge, professionals felt it was crucial to ensure patients are *"well equipped with the information that they need" [professional, BCH006].* This encompassed information related to support the self-management of the wound (e.g. creaming, massaging, sun protection). Professionals' main objectives were also to help patients understand their injury (e.g. recognise signs of infection) and gain self-confidence in further wound treatment, so they remain as independent from the service as possible. Interviewees also repeatedly mentioned the importance of communicating the constant support available to patients which included readiness to answer any questions and psychological support.

> *"It's explaining to them what they need to be doing to achieve this wound to heal" [professional, NBT026]]*

> *"My biggest goal and concern for them is can they access everything they want to access." [professional, BCH007]*

Uncertainty (patients only)

Uncertainty was identified as a unique theme for patients and was an important concern during the earlier stages of recovery. Feelings of uncertainty related to how the injury happened, the treatments needed and their possible outcomes as well as the likely course of recovery. Participants associated feelings of anxiety with uncertainty but also described how information provision (e.g.

For peer review only - http://bmjopen.bmj.com/site/about/guidelines.xhtml

about recovery and further treatment) can lessen those feelings. For patients with more severe burns, uncertainties remained in the long-term and were mostly related to scar management and future treatment plans.

> *“During the time that we were kind of up in the air as to whether he would have the operation or not, we saw loads of different people on different rotas and stuff, and I kind of felt like there were lots of different opinions flying around about how well it was healing, how well it wasn’t healing, what the next course of treatment would be. And although I understand why that was the case, because in the main they were kind of waiting to see what the wound was going to do, we felt very up in the air about […] what was going to happen, and whether what happened eventually would have actually been the right thing.” [patient, BCH017]*

> *“Yeah, you’ve got no information, no knowledge and the pain materialises, you don’t know whether that’s supposed to happen, was that not supposed to happen, is something going wrong.” [patient, NBT012]*

Sense of control (professionals only)

For a smaller number of professionals, the sense of feeling in control of the treatment process was identified as a priority in the short term. This included major concerns about being in control of the treatment situation (e.g. managing distress well) and technical steps involved. Professionals described the importance of feeling well prepared for the patients’ treatment and the presence of a well-functioning team to support a smooth treatment. Consequence of lack of control were mentioned and included impacts on self-confidence and patients’ confidence in the care.

> *“It’s being in control of that procedure. I feel as a clinical professional, […] once you’ve lost the control of the procedure in some way then you’ve lost the parents’ confidence in you.” [professional, BCH006]*

Survival (professionals only)

Some professionals mentioned survival to be the key priority in the short term and was only discussed in the context of larger burns. For those professionals involved in their care, mortality-related outcomes and physiological signs (e.g. organ failure, fever) were a major concern. Only a minority of patients experienced life-threatening injuries, but those with major burns generally did not discuss survival. This may be caused by *“a kind of hole in their memory and they don’t remember what happened to them” [professional, MH006]*, aggravated by severe pain relief and reduced levels of consciousness.

For peer review only - http://bmjopen.bmj.com/site/about/guidelines.xhtml

"Well you'd be looking at their physical signs and outcomes, things like their vital signs, their bloods, whether they're being ventilated and they're on inotropes, and also whether they're having runs of sepsis and whether they've gone into acute renal failure" [professional, NBT003]

"The most important outcome is survival because there are those patients that are very poorly and you just want them to survive". [professional, BCH007]

Mapping themes to outcomes (objective iii)

A total of seven out of 10 themes (70%) were identified as recognised outcomes in burn research. These were evident in a recently published systematic review that investigated all outcomes reported in burn care research [14] and identified research studies (see Table 2). An overview of identified exemplary studies assessing an outcome for which conceptual content aligns with themes identified in this study is shown below.

For peer review only

For peer review only - http://bmjopen.bmj.com/site/about/guidelines.xhtml

Table 2 Comparison of identified themes and outcomes measured in trials

Themes identified	Outcome domains mapped for both patients and professionals	Outcome domains mapped for patients only	Outcome domains mapped for professionals only
**Pain and discomfort**	• Effect of scar on movement (contractures)<br>• Burn wound pain	• Pain during procedures<br>• Itch<br>• Scar pain<br>• Comfort of dressings	• Use of medicines to treat symptoms
**Psychological wellbeing**	• Quality and quantity of sleep<br>• Psychological well-being<br>• Generalised anxiety<br>• Mental ability		
**Function**	• Ability to carry out daily tasks<br>• Return to work/school or previous function	• Appearance	• Mobility
**Healing**	• Burn wound healing	• Appearance	
**Infection**	• Burn wound infection	• Sepsis	
**Survival**		• Death from burn injury<br>• Death from any cause	
**Uncertainty**	None identified		
**Patient knowledge, understanding & support**	None identified		
**Sense of control**	None identified		

For peer review only - http://bmjopen.bmj.com/site/about/guidelines.xhtml

There were three themes (30%) for which no outcome domains could be mapped. In particular, 'uncertainty', 'patient knowledge, understanding & support' and 'sense of control' were all themes uniquely identified in this study [14].

DISCUSSION

This study used semi-structured interviews to investigate perspectives of 53 burn patients, and professionals involved in their care, to explore what is most important as the patients recover. The study identified a total of ten themes across both participant groups that related to priorities during recovery from a burn injury. Of those, six were identified across both participant groups ('pain and discomfort, 'psychological wellbeing', 'healing', 'scarring', 'function' and 'infection'), three were unique to professionals ('patient knowledge, understanding & support', 'sense of control', 'survival') and one was unique to patients ('uncertainty'). The study results also showed that the importance of recovery priorities changes over time (e.g. 'survival' was a concern in the short term but not in the medium to long term), and that priorities differed between patients and professionals (e.g. 'pain and discomfort' was a concern in the short to medium term for professionals but important to many patients throughout their recovery). Finally, this work established that seven identified themes overlapped with outcomes commonly reported in burn research, whereas three themes could not be mapped to reported outcomes ('uncertainty', 'patient knowledge, understanding & support', 'sense of control').

This work identified unique differences between what patients and professionals view as most important during burn recovery and supports the temporal sensitivity of priorities of patients and professionals. We were unable to find existing empirical evidence from burn care research that shows similar findings. This work, however, does complement other recent efforts to improve patient-centred outcome measurement in burn care research [3,6,7,12] by highlighting the need to align outcome assessment with the changing importance of priorities for different participant groups, and with the change in relevance of priorities at different times after injury or intervention. Research in other healthcare areas has started to acknowledge these complexities of outcome assessment during recovery. Development of a core outcome set for childhood asthma, for example, reported the differences in perspectives of patients and professionals as to which outcomes are most important [32]. Other core outcome sets for lower limb amputation or induction of labour, for example, included separate outcomes relevant to short and medium term recovery [33–35]. This

For peer review only - http://bmjopen.bmj.com/site/about/guidelines.xhtml

study supports a recommendation for core outcome sets in burns care to differentiate between different time points [3], but current core outcome sets do not yet incorporate these complexities [16].

An aim of on-going research is to ensure that frequently measured outcomes adequately capture all important priorities related to burns recovery [6]. The results from mapping themes to commonly measured outcomes in burn care research highlighted potentially important priorities currently not considered. . Specifically, perceptions related to 'uncertainty' (patients), 'sense of control' and 'patient knowledge, understanding & support' (professionals) were all identified as important priorities at varying time points during recovery. The existence and impacts of uncertainty on patients are well known [36] and, as supported by our findings, and have also been linked to the level of information provision [37,38]. Conversely, professionals in our study highlighted that 'patient knowledge, understanding & support' is one of their key priorities during the medium term of patients' recovery from a burn injury. Routine assessment of perceptions relevant to level of uncertainty and information provision as well as assessing patients' level of knowledge and understanding and support received can be important beyond commonly measured outcomes. In addition, these measures of subjective perception might provide useful care quality indicators to highlight where improvements, e.g. in information provision, might be required. Measurement instruments assessing patients' sense of control in healthcare interventions exist [39,40]. Very few studies, however, report the need to feel in control from the perspectives of healthcare professionals [41], and we were unable to find a measure assessing professional specific sense of control. In our study, only a small number of professionals mentioned this as a priority which warrants further research investigating the importance and impacts of perceived sense of control.

Strengths and limitations

Our findings integrated views from a large sample of patients and healthcare professionals (n=53) from various backgrounds, allowing investigation of a variety of perspectives. This means that the findings are broadly representative of commonly occurring burn injuries but simultaneously may not account for unique concerns of specific patient groups. For instance, where burn injuries were related to mental health issues (e.g. self-inflicted injuries), patients may display different patterns of priorities. Similarly, analysis focused on the most important and most commonly raised concerns which implies that individual experiences and priorities may differ to themes identified in this study. Other limitations should be acknowledged. First, our sample consisted of patients with a variety of burn size which has implications for elicited perceptions across different time frames investigated.

For peer review only - http://bmjopen.bmj.com/site/about/guidelines.xhtml

For instance, patients with larger burns experience longer healing [42].This study defined time frames (short, medium and long term) *a priori* which were agreed after expert consultation and discussions within the multi-disciplinary study team. Individuals' definitions of short, medium and long term recovery, however, are highly personal and might depend on contextual complexities (e.g. size and location of burn, time since injury, socio-demographic details of patients, any wound complications), and might therefore differ to the definitions used in this study. Challenges to standardise time frames for recovery from burn injury are recognised considering the variabilities and complexities of injuries [42–44]. Long term outcome measurement, for example, can range from a few months [45] to years [46] across studies assessing functional outcomes for large burns. Further research is warranted to pursue consensus definitions of time frames in burn recovery. In addition, time periods between participants' time of injury and interview varied across the sample, but no systematic variation in length of experience of recovery was pursued during recruitment. Due to resource limitations and practical considerations, the current work also did not employ a longitudinal study design but merely provides a snapshot of participants' perceptions. Quantitative and qualitative evidence suggests that varying recall periods may influence data collection in health research [47,48]. We were unable to collect reliable data on injury date to be able to assess the impact of potential recall bias caused by varying intervals on the results.

Second, our recruitment strategy meant that some patient groups may be underrepresented (e.g. patients with larger burns, adolescent patients). A small number of qualitative studies showed that different population groups experience recovery differently. For example, women were better able to adapt to living with a burn injury [49] and older adults require more support and information [50] following severe burn injury. Future research could explore priorities in more homogeneous patient groups with larger sample size, to investigate whether these change between different populations and type of injury. Likewise, professional participants were mainly female and had nursing roles and there were challenges in recruiting surgeons, mainly due to individuals' time constraints. It is unclear if, for example, perspectives of male and female professionals differ and whether a more gender balanced sample would have impacted the results. There is value in exploring this difference in future work. It is also acknowledged that our sample lacks ethnic diversity. Again, it would be of value to explore perspectives over time specific to certain ethnic minority groups to reveal potentially unique and important culturally dependent priorities [51,52].

Continued consensus work to refine existing core outcome and core measurement sets is crucial. Modular components of core sets using findings from this study could be developed, whereby different sets of outcomes are considered "core" to address priorities specific to time points and specific to different stakeholder groups. The use of applicable modules can accommodate

For peer review only - http://bmjopen.bmj.com/site/about/guidelines.xhtml

For peer review only

differences in stakeholder priorities and optimise outcome measurement relevant to short, medium and long term priorities. This would reduce research waste and outcome measurement burden, but requires effective strategies for implementation in practice [53]. Further research is also needed to better understand impacts of temporal sensitivity of outcomes during the recovery from burn injuries. This work provided a starting point to understanding the variability of the most important and broad priorities of patients and professionals during burn recovery but does not provide an outcome categorisation. Additional qualitative work might assess more granular aspects of themes identified in this work amongst more homogenous patient groups by drawing on existing outcome taxonomies [6,7,14,54]. This can include, for instance, exploring more nuanced psychological concepts that are important to specific patient groups (e.g. adolescents or ethnic minority patients) or specific injury patterns (e.g. large burns) over time. Likewise, priorities important across all stages of recovery require further detailed examination to be able to inform specific outcome measurement. Indeed, this work does not attempt to recommend how outcomes should be measured and further in-depth work is required for this. For example, different concepts related to pain (scar pain, burn wound pain, pain during the procedure) might be relevant at different time points. Further empirical work is necessary to explore commonly understood time frames for recovery periods (e.g. short, medium, long term) which may facilitate the standardisation of time frames for outcome measurement in trials. This would allow efficient integration of essential and meaningful outcome measures into trial design, avoiding research waste and enabling tailored treatment and intervention development in burns care.

CONCLUSION

The study recommends that important outcomes in burns care are measured at different time points during the recovery from a burn injury. In addition, it is suggested that outcomes are considered for both patients and professionals. Enhanced recovery assessment through the inclusion of priorities not currently considered has the potential to uncover important areas for improvement of outcomes assessment. This may ultimately benefit burn victims by effectiveness and improved patient-centred care. Further work is needed to elucidate nuances of outcomes for specific patient groups and/or specific burn presentations and harmonise time periods across contexts for efficient integration of temporal sensitivity of outcome measurement.

For peer review only

For peer review only - http://bmjopen.bmj.com/site/about/guidelines.xhtml

REFERENCES

1 NHS Digital. HES Data Dictionary: Admitted Patient Care. 2019.

2 NHS England. NHS STANDARD CONTRACT FOR SPECIALISED BURNS CARE (ALL AGES). 2013. https://www.england.nhs.uk/wp-content/uploads/2014/04/d06-spec-burn-care-0414.pdf (accessed 5 Jun 2021).

3 Falder S, Browne A, Edgar D, *et al.* Core outcomes for adult burn survivors: A clinical overview. Burns. 2009;**35**:618–41. doi:10.1016/j.burns.2008.09.002

4 Brown T, Mills S, Muller M. If it can't be measured it can't be managed: The paucity of outcome measures in burn care. Burns. 2003;**29**:757. doi:10.1016/j.burns.2003.09.006

5 Lemon J, Cooper J, Defres S, *et al.* Understanding parental perspectives on outcomes following paediatric encephalitis: A qualitative study. *PLoS One* 2019;**14**:1–15. doi:10.1371/journal.pone.0220042

6 Mathers J, Moiemen N, Bamford A, *et al.* Ensuring that the outcome domains proposed for use in burns research are relevant to adult burn patients: A systematic review of qualitative research evidence. *Burn Trauma* 2021;**8**:30. doi:10.1093/BURNST/TKAA030

7 Jones LL, Calvert M, Moiemen N, *et al.* Outcomes important to burns patients during scar management and how they compare to the concepts captured in burn-specific patient reported outcome measures. *Burns* 2017;**43**:1682–92. doi:10.1016/j.burns.2017.09.004

8 Mc Kittrick A, Gustafsson L, Hodson T, *et al.* Exploration of individuals perspectives of recovery following severe hand burn injuries. *Burns* Published Online First: 29 April 2022. doi:10.1016/J.BURNS.2022.04.026

9 Robert R, Meyer W, Bishop S, *et al.* Disfiguring burn scars and adolescent self-esteem. *Burns* 1999;**25**:581–5. doi:10.1016/S0305-4179(99)00065-0

10 Mathers J. Towards the Holistic Assessment of Scar Management Interventions. *Eur Burn J 2022, Vol 3, Pages 207-210* 2022;**3**:207–10. doi:10.3390/EBJ3010018

11 Kornhaber RA, De Jong AEE, McLean L. Rigorous, robust and systematic: Qualitative research and its contribution to burn care. An integrative review. *Burns* 2015;**41**:1619–26. doi:10.1016/J.BURNS.2015.04.007

12 Simons M, Price N, Kimble R, *et al.* Patient experiences of burn scars in adults and children and development of a health-related quality of life conceptual model: A qualitative study. In: *Burns*. Elsevier Ltd 2016. 620–32. doi:10.1016/j.burns.2015.11.012

13 Kool MB, Geenen R, Egberts MR, *et al.* Patients' perspectives on quality of life after burn. *Burns* 2017;**43**:747–56. doi:10.1016/j.burns.2016.11.016

14 Young AE, Davies A, Bland S, *et al.* Systematic review of clinical outcome reporting in randomised controlled trials of burn care. *BMJ Open* 2019;**9**:e025135. doi:10.1136/BMJOPEN-2018-025135

15 Simons M, Lim PCC, Kimble RM, *et al.* Towards a clinical and empirical definition of burn scarring: A template analysis using qualitative data. *Burns* 2018;**44**:1811–9. doi:10.1016/J.BURNS.2018.04.006

16 Young A, Brookes S, Rumsey N, *et al.* Agreement on what to measure in randomised controlled trials in burn care: Study protocol for the development of a core outcome set. *BMJ*

For peer review only

Open 2017;**7**:e017267. doi:10.1136/bmjopen-2017-017267

17 Williamson PR, Altman DG, Blazeby JM, *et al.* Developing core outcome sets for clinical trials: Issues to consider. *Trials* 2012;**13**:1–8. doi:10.1186/1745-6215-13-132

18 O'Brien BC, Harris IB, Beckman TJ, *et al.* Standards for reporting qualitative research: A synthesis of recommendations. *Acad Med* 2014;**89**:1245–51. doi:10.1097/ACM.0000000000000388

19 Bunniss S, Kelly DR. Research paradigms in medical education research. *Med Educ* 2010;**44**:358–66. doi:10.1111/J.1365-2923.2009.03611.X

20 Patton MQ. *Qualitative research & evaluation methods : integrating theory and practice*. SAGE Publications 2002.

21 Van Baar ME, Essink-Bot ML, Oen IMMH, *et al.* Functional outcome after burns: A review. *Burns* 2006;**32**:1–9. doi:10.1016/J.BURNS.2005.08.007

22 Braun V, Clarke V. Using thematic analysis in psychology. *Qual Res Psychol* 2006;**3**:77–101. doi:10.1191/1478088706qp063oa

23 Joffe H, Yardley L. Content and thematic analysis. In: Marks D, Yardley L, eds. *Research methods for clinical and health psychology*. London: : Sage 2004. 56–69.

24 Fusch PI, Ness LR. Are We There Yet? Data Saturation in Qualitative Research. *Qual Rep* 2015;**20**:1408–16.http://www.nova.edu/ssss/QR/QR20/9/fusch1.pdf (accessed 12 May 2022).

25 Hennink MM, Kaiser BN, Marconi VC. Code Saturation Versus Meaning Saturation: How Many Interviews Are Enough? *Qual Health Res* 2017;**27**:591–608. doi:10.1177/1049732316665344

26 Saunders B, Sim J, Kingstone T, *et al.* Saturation in qualitative research: exploring its conceptualization and operationalization. *Qual Quant* 2018;**52**:1893. doi:10.1007/S11135-017-0574-8

27 Fofana F, Bazeley P, Regnault A. Applying a mixed methods design to test saturation for qualitative data in health outcomes research. *PLoS One* 2020;**15**. doi:10.1371/JOURNAL.PONE.0234898

28 Nelson J. Using conceptual depth criteria: addressing the challenge of reaching saturation in qualitative research: *https://doi.org/101177/1468794116679873* 2016;**17**:554–70. doi:10.1177/1468794116679873

29 Morse JM. Data were saturated... *Qual Health Res* 2015;**25**:587–8. doi:10.1177/1049732315576699

30 Morse WC, Lowery DR, Steury T. Exploring Saturation of Themes and Spatial Locations in Qualitative Public Participation Geographic Information Systems Research. *http://dx.doi.org/101080/089419202014888791* 2014;**27**:557–71. doi:10.1080/08941920.2014.888791

31 Edlich RF, Larkham N, O'Hanlan JT, *et al.* Modification of the american burn association injury severity grading system. *J Am Coll Emerg Physicians* 1978;**7**:226–8. doi:10.1016/S0361-1124(78)80381-5

32 Sinha IP, Gallagher R, Williamson PR, *et al.* Development of a core outcome set for clinical trials in childhood asthma: a survey of clinicians, parents, and young people. *Trials 2012 131* 2012;**13**:1–9. doi:10.1186/1745-6215-13-103

For peer review only

For peer review only - http://bmjopen.bmj.com/site/about/guidelines.xhtml

33 Ambler GK, Brookes-Howell L, Jones JAR, *et al.* Development of Core Outcome Sets for People Undergoing Major Lower Limb Amputation for Complications of Peripheral Vascular Disease. *Eur J Vasc Endovasc Surg* 2020;**60**:730–8. doi:10.1016/J.EJVS.2020.06.021

34 Ambler GK, Bosanquet DC, Brookes-Howell L, *et al.* Development of a core outcome set for studies involving patients undergoing major lower limb amputation for peripheral arterial disease: study protocol for a systematic review and identification of a core outcome set using a Delphi survey. *Trials 2017 181* 2017;**18**:1–7. doi:10.1186/S13063-017-2358-9

35 Dos Santos F, Drymiotou S, Antequera Martin A, *et al.* Development of a core outcome set for trials on induction of labour: an international multistakeholder Delphi study. *BJOG An Int J Obstet Gynaecol* 2018;**125**:1673–80. doi:10.1111/1471-0528.15397

36 Auerbach SM, Martelli MF, Mercuri LG. Anxiety, information, interpersonal impacts, and adjustment to a stressful health care situation. *J Pers Soc Psychol* 1983;**44**:1284–96. doi:10.1037/0022-3514.44.6.1284

37 Andrew JM. Recovery from surgery, with and without preparatory instruction, for three coping styles. *J Pers Soc Psychol* 1970;**15**:223–6. doi:10.1037/H0029442

38 Gammon J, Mulholland CW. Effect of preparatory information prior to elective total hip replacement on post-operative physical coping outcomes. *Int J Nurs Stud* 1996;**33**:589–604. doi:10.1016/S0020-7489(96)00019-3

39 Tomsis Y, Perez E, sharabi L, *et al.* Postpartum Post-Traumatic Stress Symptoms Following Cesarean Section—the Mediating Effect of Sense of Control. *Psychiatr Q 2021 924* 2021;**92**:1839–53. doi:10.1007/S11126-021-09949-0

40 Lau-Walker MO, Cowie MR, Roughton M. Coronary heart disease patients’ perception of their symptoms and sense of control are associated with their quality of life three years following hospital discharge. *J Clin Nurs* 2009;**18**:63–71. doi:10.1111/J.1365-2702.2008.02386.X

41 Southwick FS, Southwick SM. The Loss of a Sense of Control as a Major Contributor to Physician Burnout: A Neuropsychiatric Pathway to Prevention and Recovery. *JAMA Psychiatry* 2018;**75**:665–6. doi:10.1001/JAMAPSYCHIATRY.2018.0566

42 Pham TN, Kramer CB, Wang J, *et al.* Epidemiology and outcomes of older adults with burn Injury: An analysis of the national burn repository. *J Burn Care Res* 2009;**30**:30–6. doi:10.1097/BCR.0b013e3181921efc

43 Ryan CM, Lee A, Kazis LE, *et al.* Recovery trajectories after burn injury in young adults: Does burn size matter? *J Burn Care Res* 2015;**36**:118–29. doi:10.1097/BCR.0000000000000214

44 Mason ST, Esselman P, Fraser R, *et al.* Return to work after burn injury: A systematic review. J. Burn Care Res. 2012;**33**:101–9. doi:10.1097/BCR.0b013e3182374439

45 Cucuzzo NA, Ferrando A, Herndon DN. The Effects of Exercise Programming vs Traditional Outpatient Therapy in the Rehabilitation of Severely Burned Children. *J Burn Care Rehabil* 2001;**22**:214–20. doi:10.1097/00004630-200105000-00006

46 Druery M, Brown TLH, Muller M. Long term functional outcomes and quality of life following severe burn injury. *Burns* 2005;**31**:692–5. doi:10.1016/J.BURNS.2005.03.001

47 Stull DE, Leidy NK, Parasuraman B, *et al.* Optimal recall periods for patient-reported outcomes: challenges and potential solutions. *https://doi.org/101185/03007990902774765* 2009;**25**:929–42. doi:10.1185/03007990902774765

48 Dolan AR, Goldberg EB, Cannuscio CC, *et al.* Patient Perceptions About Opioid Risk

For peer review only - http://bmjopen.bmj.com/site/about/guidelines.xhtml

Communications Within the Context of a Randomized Clinical Trial. *JAMA Netw Open* 2022;**5**:e2227650–e2227650. doi:10.1001/JAMANETWORKOPEN.2022.27650

49 Williams NR, Davey M, Klock-Powell K. Rising from the Ashes. *http://dx.doi.org/101300/J010v36n04_04* 2008;**36**:53–77. doi:10.1300/J010V36N04_04

50 Jones BA, Buchanan H, Harcourt D. The experiences of older adults living with an appearance altering burn injury: An exploratory qualitative study. *J Health Psychol* 2017;**22**:364–74. doi:10.1177/1359105315603473

51 Rawlins JM, Khan AA, Shenton AF, *et al.* Burn patterns of Asian ethnic minorities living in West Yorkshire, UK. *Burns* 2006;**32**:97–103. doi:10.1016/j.burns.2005.08.013

52 Tan KT, Prowse PM, Falder S. Ethnic differences in burn mechanism and severity in a UK paediatric population. *Burns* 2012;**38**:551–5. doi:10.1016/j.burns.2011.10.005

53 Hughes KL, Clarke M, Williamson PR. A systematic review finds Core Outcome Set uptake varies widely across different areas of health. *J Clin Epidemiol* 2021;**129**:114–23. doi:10.1016/J.JCLINEPI.2020.09.029

54 Dodd S, Clarke M, Becker L, *et al.* A taxonomy has been developed for outcomes in medical research to help improve knowledge discovery. *J Clin Epidemiol* 2018;**96**:84–92. doi:10.1016/j.jclinepi.2017.12.020

For peer review only

For peer review only - http://bmjopen.bmj.com/site/about/guidelines.xhtml

Acknowledgements

We would like to thank the research nurses Karen Coy, Catherine Spry and Anthony Sack for their help with recruitment for this study. We also thank all participants for dedicating their time and views to this study.

Professor Amber Young, senior author, colleague, and friend sadly passed away before the publication of this manuscript. Amber dedicated her career to advancing burn care research and worked tirelessly to improve outcomes for burn survivors. It was our privilege to work with Amber and to benefit from her skills, passion, and knowledge. She will be truly missed.

Contributors

AEY conceived the project. CH wrote the manuscript with the support of AEY, PD and DE. CH and AEY performed data collection. CH and PD performed data analysis with support of AYE and DE. DE is a qualitative researcher and provided oversight and guidance on methods. AEY and DE edited and critically revised draft manuscripts. AYE provided strategic oversight over the project. All authors have read and approved the publication.

Funding

This research is funded by The Scar Free Foundation, UK. The Scar Free Foundation is the only medical research charity focused on scarring with the mission to achieve scar free healing within a generation. The project was also supported by the National Institute for Health Research (NIHR) Biomedical Research Centre (BRC) at the University Hospitals Bristol and Weston NHS Foundation Trust and the University of Bristol (BRC-1215-20011). AY has received an NIHR Doctoral Research Fellowship (DRF-2016-09-031 NIHR). This paper represents independent research funded by The Scar Free Foundation. The views expressed in this publication are those of the authors and do not necessarily reflect those of the Scar Free Foundation, the UK NHS or the NIHR.

Competing interests

All authors declare no competing interests.

For peer review only

Patient consent for publication

As part of the consent process, patient participants agreed to their anonymised quotes being published in scientific journals.

Ethics approval

A favourable opinion for the project was granted by the South West - Frenchay Research Ethics Committee, reference 17/SW/0025. AY and CH obtained the written informed consent of each participant before conducting the interviews. It was also ensured participants had read the study information sheet sent to them prior to the interview and that they were informed about their rights as participants, that the conversation would remain confidential, and that the transcript would be anonymised.

Provenance and peer review

Not commissioned; externally peer reviewed

Data availability statement

All relevant data is included in the main body or the supplementary files. The raw datasets used and analysed during the current study are available from the corresponding author upon reasonable request.

For peer review only

For peer review only - http://bmjopen.bmj.com/site/about/guidelines.xhtml

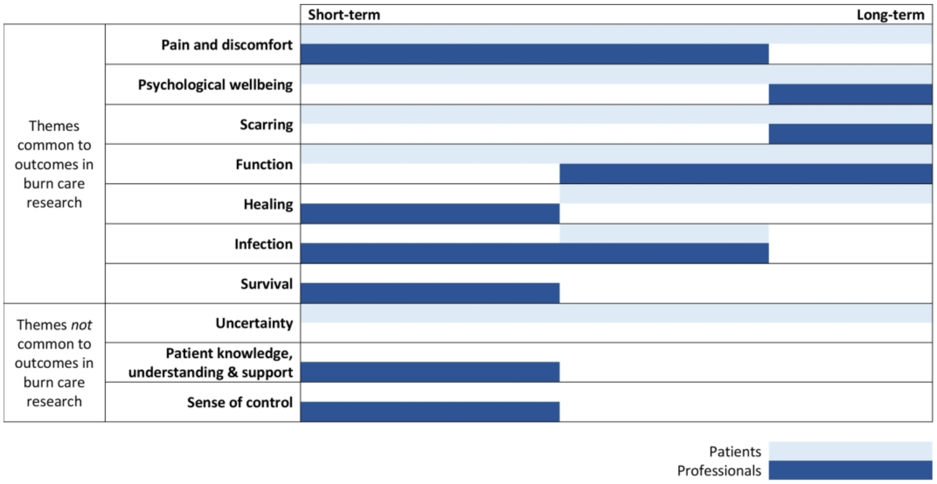

Figure 1: Overview of patients' and professionals' priorities over time

246x129mm (300 x 300 DPI)

Supplemental File - Table S1: Exemplary interview topic guide for patient participants

Topic	Questions
**Intro**	• Introduction to researcher, topic, aims and objectives of the study.<br>• Ask if any questions<br>⇒ *Check that participant is happy to audio record the interview and check consent*
**Background/ warm-up**	• First of all, could you tell me a bit about you?<br>○ *Prompt: do you work, do you have children etc*<br>○ *Prompt for children: how old are you?*<br>• Could you tell me about how you/your child came to have the burn?
**Short-term: Immediately after injury**	• Thinking about the time when you were burned, what were your worries about the immediate effects of the burn at that time?<br>○ *Prompt: e.g. survival, pain*<br>○ *Anything else you can think of? Any other concerns you may have had?*<br>• Still thinking about the time when you were burned, what were your concerns about future effects?<br>○ *Prompt: scarring, movement*<br>*Areas of your life that it was most important to ensure the burn didn't affect eg function (eg related to work, school), the look of the burn (confidence, work, school, friends)? Anything else?*<br>• **What would you say were the most important aspects during that time?**
**Medium-term: After discharge**	• Thinking about the time when you started to recover/got home/were discharged, what were your concerns in relation to your recovery?<br>○ *Was your daily life affected? If so, in what ways? Return to normal.*<br>▪ *Prompts could include work life; social life; family life; relationships (including intimacy)*<br>○ *Were there any issues that affected you that the doctors didn't ask about at your out-patient appointments?*<br>○ *Were there issues that bothered you more than they seemed to bother the medical staff?*<br>○ *Were there issues that the doctors asked about that didn't worry you?*<br>• During the recovery (at home), what were your concerns specifically about the future?<br>○ Prompt: QoL, physical,<br>• Were your family and friends (and if applicable, partner) worried about any effects of your burn at any time after the burn injury? If so, what were they worried about?<br>○ *Prompt: (as above; work life; social life; family life; relationships (including intimate relationships)*<br>○ *Anything else? What other concerns were there?*<br>• **What, in your view, was most important to you during the time after discharge?**
**(Current) Recovery & Future**	• What matters to you (now) while you are getting better?<br>○ *Prompts: similar to above and laddering questions*<br>• And thinking about the future, what are you particularly worried about?<br>• **And again, thinking about your situation now, what is most important to you at the moment in relation to your recovery?**<br>○ Do you think this will matter further down the line?<br>○ If there are any areas of healthcare that could have been improved, what would that/they be?<br>○ *Prompt: treatment of infection, management of pain, improving success of skin-grafting?*
**Wrap-up questions**	• To finish with, could we summarise what the most important areas of your recovery after the injury were to you?<br>⇒ *Wrap up interview*

For peer review only

Supplemental File - Table S2: Exemplary interview topic guide for professional participants

Topic	Questions
**Intro**	• Introduction to researcher, topic, aims and objectives of the study.<br>• Ask if any questions<br>⇒ *Check that participant is happy to audio record the interview and check consent*
**Background/ warm-up**	• First of all, could you tell me a bit about you and your role as a healthcare professional?<br>○ *Prompt: how long have you been at the hospital for, what are your responsibilities?*<br>○ *What is your involvement with burns injuries? How long working in burns?*<br>• What are your experiences been with burns patients?<br>• *Which part of the recovery journey are you involved in? When/how regular do you see them?*
**Short-term: Immediately after injury**	• Going back to the very beginning, when patients are admitted to the hospital, what are the relevant outcomes from a clinical perspective?<br>○ E.g. survival, scaring, pain?<br>• **What is the most important outcome?**<br>• What do you think patients are worried about the most during that time?<br>• **What do you think is the most important outcome for patients at that time?**
**Medium-term: After discharge**	• Moving on to when the patient gets discharged for recovery at home, what are relevant outcomes for you from a clinical viewpoint?<br>• **And what was the most important one taking into consideration the recent discharge?**<br>• And again, what do you think the patients are concerned about when they get discharged from hospital?<br>○ *Prompt: daily life, QoL, work, social,*<br>• **What do you think is most important to the patients at the time they are being discharged?**<br>• How do you think do the patients views differ from their families and friends? What are they worries about?<br>• *Prompt: (as above; work life; social life; family life; relationships (including intimate relationships)*
**(Current) Recovery & Future**	• Thinking about burns injury outcomes further down the line, what is important for you in the long run? What is most important?<br>○ What do you think is the most important to patients when thinking about the really long-term effects in the future?
**Wrap-up questions**	• To finish with, could we summarise what the most important areas of patient recovery after the injury?<br>⇒ *Thanks to interviewee*

Supplemental File – Table S3: Outcome classification for burn care research (adapted from Young et al. 2019)

**Outcome category**	**Outcome domain**
**Patient-reported**	Ability to carry out daily tasks
	Anxiety about medical procedures and appointments
	Generalised anxiety
	Appearance
	Blister fluid
	Burden of care
	Comfort of dressings
	Psychological well-being
	Mental ability
	Quality and quantity of sleep
	Effect of scar on movement (contractures)
	Return to work/school or previous function
	Burn wound pain
	blister fluid
	Pain during procedures
	Scar pain
	Itch
**Pathophysiological**	Ability to fight infection
	Body weight maintenance
	Bone strength
	Breathing and lungs
	Donor site problems after healing
	Effect of burn on genes
	Effect of burn on how the body uses energy
	Effect on heart and blood circulation
	Fitness
	Growth in children
	How well muscles work
	Mobility
	Kidney function
	Liver function
	Medical tests to indicate how unwell a patient is
	More than one organ failing (multiorgan failure)
	Muscle strength
	Stomach and bowel function
	Burn wound healing
	Donor site healing
**Complications**	Complications of drug treatment
	Blood product transfusion
	Burn wound infection
	Death from burn injury
	Death from any cause
	Effects of fluid from a drip
	Infections other than burn wound infection

For peer review only

	Sepsis
**Scar-related**	Scar colour
	Scar texture
	Scar size
	Treatment for scars
**Healthcare-related**	Costs of treatment for NHS/hospital
	Length of hospital stay
	Length of stay in intensive care unit
	Length of time on life support machine
	Use of medicines to treat symptoms

Young AE, Davies A, Bland S, Brookes S, Blazeby JM. Systematic review of clinical outcome reporting in randomised controlled trials of burn care. BMJ open. 2019 Feb 1;9(2):e025135.

For peer review only

Supplemental File – Descriptive reports

1. Interviews with patients

Scarring

Worries about future scarring were apparent for some participants and their families in the early days following the injury.

> *Well the worries were the scarring and whether we'd need to have- at that point in time we didn't know- no-one could tell us how bad it was. Before we got to (city) burns no-one could tell us how bad it was, so our worry was whether she needed to have a skin graft or was she going to have permanent scars because it was so horrible on her face-[BCH 01, parent]*

> *Just wondering what the scarring would do, whether the skin would be very tight. Because you do see people who've had burns, like facial burns and sometimes their skin is very pink and red and then you do notice it. But yes, I think that was about it really. It's just how functional your hands would be when they're recovered.[NBT 27]*

The majority of participants interviewed went on to develop some scarring as their wound healed. Many of these participants described the physical appearance of their scars, talking about the colour (*'really pink' [BCH FW] 'quite red, it's not a natural skin colour [NBT 005]*) and the shape (*'quite gnarly' [NBT 02], 'pretty bumpy' [BCH 19]*). One participant talked about how the appearance of their scar was getting worse during the healing process.

> *you look progressively worse as your function gets progressively better. So I had a- I struggled with the fact that I was physically feeling up to it, going out, doing things and I was looking more scarred, more bumpy because scar tissue takes a while to form and, you know, you just end up, as the peeling process happens, looking worse as time goes on until a point where then you'll stop and it will start to improve but you need to do a lot to improve the appearance so for the last three to four months I've been looking progressively worse.[C&W 004]*

Several participants talked about how their scars negatively affected their feelings about their appearance.

> *I felt I looked like Frankenstein [CW 002]*

> *How it looked was a big- I wouldn't be able to go out on a beach and just feel confident in a bikini or something [NBT 002]*

> *I worry this will be it, it's not going to get any better but I've said that every month since I was discharged and there's always been changes. But I cry… every time I go in I cry going is this it? I can't look like this for the rest of my life. [NBT 013]*

The reactions of others to their scars also had an impact. Several participants described being stared at by others. For some, the reactions of others were a strong determinant of how they themselves felt about their scars.

> *some guy on the bus, this was a grown man in his thirties, I was on the bus on my way to hospital once and I was standing 'cos there were no seats, he was looking at me and doing wretch noises as though he was vomiting, looking at me and doing-*

For peer review only

if other people were like accepting of it I would be more accepting of it, I wouldn't be as embarrassed but you get these people that are just really naïve. [C&W 002]

It's more a question of having an overall appearance that causes people to look at you in the street which is uncomfortable. If you had scars that were maybe subtle, not a big deal, some people might notice when they're close up but I think, you know, as the healing process happens with burns you go very red, very bumpy, it's really obvious and I think anything obvious in terms of the reaction you see from other people affects you more than how you see yourself. [C&W 004]

However, some participants said that they were not concerned about their scars.

Personally doesn't bother me, doesn't bother me at all. I can see why it might bother some people but personally no. [NBT 08]

Yeah, don't get me wrong, its not nice to have a scar like that but its not the end of the world, I'm not too vain that I can't still put shorts on sort of thing, but yeah, it is what it is. [NBT 12]

Older participants tended to be less affected by their scarring. The location of the scar was also important – participants with scarring in less visible places tended to be less bothered by it.

It could have been on my face which the ward that I was in at the time, there was a lady opposite me in the ward that had had full body burns from her Christmas lights going up and I looked at her and I thought ok, you need to be happy about this, it could have… its an impact but its… I'm left with a scar on my leg that is not the end of the world. [NBT 12]

So it's quite visible, it's from my chin, it's very thick on my neck, you can't miss it. I get funny looks from children and so yeah, and just… I feel like I'm being remembered for the girl with the burns and stuff like that. [NBT 13]

Pain

In the immediate aftermath of the burn, pain was an important concern, often the most important concern, for the majority of patients.

'Yeah. Yeah I don't think I would have thought of anything else. Yeah, just concerned about the pain.'

'At first its just, when it first happens its just about the pain. That's it. You can't really… because it wasn't… it was only that area, I was still completely compos mentis and I was in pain but really that is just all you're focussing on at the time, its just trying to mentally manage the pain and…'

Pain was also a concern for other family members

'It is just the realisation of sort of thinking [inaudible] when I realised how much pain he was in, how painful it was to clean it, how painful it was to dress it and you he is only tiny.' [parent]

However, some participants reported that pain was not an early concern.

'Yeah, my adrenalin was going so much that I didn't notice the pain actually.'

'There was no pain really. It's really strange, yeah. I think because I think the wound got quite deep with the acid burn it sort of killed away part of the nerves-'

Pain experienced by participants occurred not just as a result of the burn but also due to treatments received such as cleaning the wound, dressing changes, surgeries and skin grafts.

For peer review only

Adequate pain management was identified as important both in the short term and after discharge from hospital.

'Yeah, they kept me very well medicated. I don't remember being in terrible pain. I remember when they would ask me every day what my pain scale (inaudible) one to whatever and I'm sure I always answered quite low because they kept it so well'.

'they literally just on the Saturday morning came up and said consultant will be coming round, he might be able to sign you off. He didn't come round, the nurse came up and said ok you're good to go and just pushed me out the door with no medication, no occupational therapist, no guidance as to what expect from the wound, no knowledge as to what to expect with pain, no pain management tablets, no nothing, they literally just pushed me out the door.'

For some participants, adequate pain management needed to be balanced against its potential side effects

'But for me I preferred to cope with that for five minutes [of wound being washed down] than take a lot of painkillers which can give you other problems. '

For participants who experienced deeper burns, pain increased in the medium term due to nerve regeneration

'I thought that it doesn't bother me because I've got on a previous burn, um, I couldn't feel anything to start off with either but gradually I'm getting little shooting pains and I spoke to one of the doctors and he said it's because all the nerves are finally like reattaching and then it's a bit confused as to where to kind of attach to so that's where the little itchy kind of sharp pain is coming from so I'm hoping, 'cos this is only two months old-' [NBT009]

Although pain was less commonly reported as an issue in the long-term, it was clear that some participants continued to experience pain or sensitivity in the affected area and this was accepted as a part of life.

'because it's just sensitive, it's the sensitivity of it. Yeah, it's very sensitive on the little finger, my thumb and the palm of my hand. But I think as she said, give that another year and it will get better. But it's fine. ' [NBT027]

'Um, I don't know if its psychological but there's always a bit of pain, I don't know but you get used to it but I was worried at first could I do any movement or not, you know, because I didn't want to damage- 'cos they've done such good work as well.' [NBT007]

'So biggest problem is sometimes, you know, a bit of pain in the leg for example, but it doesn't stop me doing things, you know, like walking but sometimes like this morning I couldn't (inaudible) and its more painful than- it is perhaps because its tighter than it should be, I don't know, but it was more painful but its easing.' [NBT007]

Itching

Itching was a commonly reported problem amongst participants. For most, itching was not experienced immediately after the injury but commenced as the wound began to heal.

Oh itching was terrible! (laughs) And not being able to actually scratch it, so the only way I could do that was to move my foot around and the dressing would sort of work its way off (laughs) so, yeah I did get a lot of itching.[NBT 014]

sometimes I think the itch was worse than the pain [C&W 06]

For peer review only

Not initially. Probably after, ooh I reckon a month. Obviously when it started to heal I think, or that's what I assumed anyway. [NBT 014]

In addition to being unpleasant, some participants reported that itching interfered with daily living. Itching was described as being worse at night which affected sleep. Two participants described their anxiety about itching in public.

Yes. Yes. I mean it itches much less than it was but when I get hot, particularly at night that's when it's such- it's worse [NBT 011]

Itchiness. I feel like that was one of the most unbearable things ever and the constant anxiety that I'm going to start itching randomly in public and I can't control it [C&W 02]

Itching continued to be a problem for a long time for some participants, although most reported that it did ease in the long term.

would say, nearly five years in now, but the itch has gone. I can remember talking to another patient that I met that had a burn injury previous to mine and they said that it took quite a few years and it did, I think it took about four years to actually- [C&W 06]

Occasionally it itches. Very occasionally. Not very often though, no, but I still get that fizzy feeling sometimes, very rarely now but occasionally [NBT 014]

Tightness

Several participants talked about tightness in the area of their burns as they healed. For some participants this tightness was a source of discomfort and distress.

when I think back to it now my burns were actually rigid, they would not move. They were literally as if they were set in stone- [C&W 002]

I think there was- the skin was quite taut so therefore uncomfortable. [NBT011]

so once I started getting the movement back I was just scared, I literally thought that all my skin was going to just rip in half. It's quite a scary experience and I'm not- I don't think I'm alone on that, she said quite a lot of people feel that way. [NBT 013]

Tightness restricted participants ability to move and impacted on their activities. It was also a source of pain.

like if I go in the garden and do some weeding because it's (inaudible)- So to then down ways, I used to bend down ways with the legs straight but they say no you have to bend down like you would so it's trying to build down- now to protect your-[NBT 007]

So biggest problem is sometimes, you know, a bit of pain in the leg for example, but it doesn't stop me doing things, you know, like walking but sometimes like this morning I couldn't (inaudible) and its more painful than- it is perhaps because its tighter than it should be, I don't know, but it was more painful but its easing. [NBT 007]

Several participants described having treatments to help with the tightness which reduced over time. However, some participants reported that they continued to experience tightness in the longer term.

For peer review only

Infection

Several participants reported experiencing one or more infections in the period following their injury. Participants described the appearance of the infected area, smell and general feelings of being unwell. The appearance and smell were also a source of distress for some patients and family members.

> *'and then on the afternoon I looked and I could see all my wrist and hand getting quite puffy and red and swollen and I thought oh I don't like the look of that' [NBT 009]*

> *'cos the infection just made me feel really ill as well.' [NBT 002]*

> *'I'm sure dad won't mind me saying, when the infection was there the smell was horrendous,' [NBT 010]*

Infections sometimes led to a lengthened hospital stay or readmission to hospital. Participants were also concerned about the possibility that infection could spread from the burn site.

> *'But I think I was most worried about the infection, when they said that it was infected and obviously it could be in his bloodstream, I think that scared me the most.' [BCH 019]*

> *'Well when the nurse mentioned sepsis and things like that you start to panic.'[NBT014]*

Worries about developing an infection were a common theme for many participants in the early period following their injury, although there was some variability in this.

> *'Yeah, so pain it was ok and you could see it was slowly getting better and I was really panicked about infections. Infections really bothered me but once the sort of the weeping stopped it was ok, you could see it was healing so that went.' [BCH 018]*

> *'I worried an awful lot about that. I did worry an awful lot about infection.' [C&W 006]*

These worries were greatest in those who had experienced a prior infection.

> *'And then I think it was probably about a month later it looked- I wondered if it was going to be infected again so I went off to the A&E and they reassured me it wasn't.' [NBT014]*

Participants were also worried about keeping the injury clean in order to prevent infection

> *'I think it was more that he has trouble staying clean, you know, he's very hands on and, you know, likes playing out in the garden and when we went swimming and things like that, I think that was-' [BCH 03]*

Worries about developing an infection were not a concern in the long-term.

Appearance of the wound

Several participants talked about the appearance of their wound in the days following their injury. Participants described the blistering, the colour and the smell of their wound. Wound appearance was a source of distress for some patients and their significant others.

> *I was pretty horrified when I saw it. I don't know whether it was just 'cos it was just raw flesh. It was just raw. It was disgusting. [C&W 005]*

> *I think initially it was the look of it because that was pretty scary, that was pretty frightening.[BCH 020]*

The effect of the wound on their appearance was also a source of distress.

For peer review only

she didn't want it [the dressing] taken off because it hurt her and then when she saw her face she just broke down. She said I'm really ugly mummy.[BCH 001]

I was just getting too upset and depressed over the fact that I had burns and I looked so different [C&W 002]

The appearance of the wound informed participants views on the seriousness of their injury.

But when it comes to over 24 hours or whatever it started to look way worse, and I was much more worried about the seriousness of it. [BCH 017]

One participant remarked that they did not realise the seriousness when the wound was dressed preventing them from being able to see it.

I had no idea how bad it was. I was very bandaged up so no. [NBT 013]

For many participants, the appearance of the wound was linked to their feelings about the time it was taking to heal.

like when its fresh you're like oh god, this is never going to heal but yeah.[NBT 002]

I don't know because although its healed lovely, it's still loitering because I can still see- I mean where the skin's purple and sometimes I get like a white raised area in the middle and I think oh what's that? And when I (inaudible) the cream it's gone. It's almost like it's sort of hang on a minute, I'm taking my time, you know. [NBT 014]

Time to healing

Some participants expressed feelings of surprise, frustration or worries about the amount of time it was taking for the wound to heal. Many seemed to feel that the healing took longer than they had expected.

I don't worry about the scar side of it, it was just the… the worry side of it was actually just the time it was taking. [NBT 012]

I was just saying to (husband) the other day, he was like I can't believe how long it's all taking 'cos it happened, I don't know when it happened, like beginning of August and now we're in October and I feel like we're still kind of still doing it. [BCH F.W.]

At the time it took ages and it was a drag coming up here every other day. [NBT 019]

Uncertainty, lack of knowledge and the need for information

A lot of participants described feelings of uncertainty following their injury. These uncertainties related to the how the injury happened, the treatments needed and their possible outcomes as well as the likely course of their recovery.

And I suppose as well, because during the time that we were kind of up in the air as to whether he would have the operation or not, we saw loads of different people on different rotas and stuff, and I kind of felt like there were lots of different opinions flying around about how well it was healing, how well it wasn't healing, what the next course of treatment would be. And although I understand why that was the case, because in the main they were kind of waiting to see what the wound was going to do, we felt very up in the air about how… what was going to happen, and whether what happened eventually would have actually been the right thing. [BCH 0017]

For peer review only

Yeah, you've got no information, no knowledge and the pain materialises, you don't know whether that's supposed to happen, was that not supposed to happen, is something going wrong. [NBT 12]

A clear need for information to lessen feelings of uncertainty was apparent. Many participants spoke positively about staff and services who met their information needs and explained things in a non-technical manner. Some participants felt that they did not get enough information and might have coped better had they had done so. For participants with more severe burns requiring a hospital stay, adequate information at the point of discharge home was important as was the availability of a point of contact for any future questions.

Very good. (A) was particularly good because he spent so much time with me 'cos he was in and out and he explained everything so thoroughly in a way that you could understand yourself, if you know what I mean, rather than having to look words up. (laughs) It's when they talk in jargon, if you know what I mean.[NBT 014]

one bit of advice that I would give throughout this is to give the patient clarity as to what's happening and the expectations where I wasn't given enough information and knowledge of what's happening now, what's going to happen, what could happen, what might not happen, I was signed off from the hospital and they hadn't said to me that the nerve endings will come back and then you'll start to suffer pain. So when I was signed off I didn't have really very much pain within 36 hours, it nearly broke me where the- going from there down to stood up, the rush of blood to the burn, I didn't know to expect it so it was quite a shock to the system. So I would just say just information, information, information. [NBT 012]

Although increased knowledge was generally seen as a positive thing, some patients mentioned that there were downsides to having too much information.

me and my family are very inquisitive people that need, you know, to know every possible scenario so there were a couple of times when we thought I'd have to have operations on things that I didn't need to have an operation on in the end so I think on the one hand it's great to be told of the things that are potentially going to go wrong or are going wrong and on the other it gave us maybe unnecessary cause for concern about procedures I ended up not needing. [C&W 004]

Several patients acknowledged that some aspects of their treatment and recovery were simply uncertain and that it would never be possible to have complete information.

I'm just obviously concerned about the future and things that no-one can tell me like in two years' time will he still need to wear the sleeve or is he still going to have to be creaming but in regards to everything that I need to know now, I feel that they've been brilliant, they've explained everything to me and to (child). [BCH 019]

Although themes of uncertainty and the need for knowledge and information were most commonly described by patients during the earlier stages of their recovery, some participants who had been left with more serious scarring described long-term term uncertainties and informational needs, particularly in relation to scar management treatments.

the problem I'm having at the moment is that there's no (sighs) there's still so few guarantees, or there are none so it's always so difficult 'cos you don't want to be making something worse if what you care about is the aesthetic. [CW 001]

And then I think in terms of, this is a big ask really but I think in terms of kind of follow-up, if there are new things, you know, I took myself off to my GP and said can you re-refer me back into the system

For peer review only

'cos I want to see if there are new things but if there was ever an opportunity to be a forum or something to be kept abreast of new developments-[CW 001]

Psychological effects

Several participants reported feelings of shock or fear in the immediate aftermath of their injury. These feelings arose as a result of the circumstances in which the injury occurred or due to the extent of the injuries received.

The first part, on the day, it was just, yeah, the whole scorch thing, the instant impact of it all going off, the shock factor.[NBT 12]

Amongst participants whose injury required a hospital stay, several participants commented on the value of psychological support being available.

just to know someone was there actually so it didn't mean that I had to deal with it on my own. I didn't make full use of it, I didn't feel the need but it was there. [C&W 004]

In the weeks following the injury, some participants reporting difficulty sleeping or of having nightmares or flashbacks.

Yeah. I do remember having quite a lot of hallucinations and I now know they're not real but at the time, for the first couple of weeks when I was up on the burns ward I didn't sleep unless they knocked me out because I lived in fear that someone was going to come and get me and put me on fire again.[NBT 0013]

Some participants also experienced guilt as they blamed themselves for the injury.

I suppose because I was so upset, and because I felt completely responsible for what had happened, I was predicting that, and I was quite vocal about that to a few people. Not many people, you know, two or three people. So that in turn made them more worried about how much kind of guilt I was taking on and I [inaudible] responsible I felt for what happened. [BCH 017]

In some cases, participants' experiences of receiving treatments also had some psychological consequences. This was more common when the person who had the burn was a child. Parents described how their child was now fearful of situations that reminded them of unpleasant or painful treatments (e.g. bathing) or of their experiences of being in hospital.

I don't know if it's got anything to do with it but like he mentally changed when he was in hospital I think because so many people were just coming into our room and hurting him he was just really upset with everybody… and I'm becoming emotional. Yeah, so even when people came to our house he was really scared of everybody so that kind of ruined our everyday life because we couldn't have friends over 'cos he'd just scream constantly at people and I think because having the nurses at home as well he couldn't trust anybody coming to our house.[BCH F.W.]

Several participants described feelings of anxiety related to general uncertainties.

Night sweats, worrying about all sorts, just want, you know, really just completely in the dark about what was going to happen and I think because there's so much forewarning that it [infection] can happen, you know, then the worst does. It's a very upsetting thing to go through. [C&W 04]

For peer review only

Several participants talked about the importance of having a positive mental attitude throughout the process in order to cope with what was happening to them. Some remarked that this was something that they felt that they had to actively work to achieve.

I've tried to stay positive, stay mentally positive the fact that it could have been worse, it could have been a lot worse, yes its happened, I can't do anything about it… From day one I was trying to get a positive mental approach on it and on that second staff infection I have to admit I was starting to get down with it because I had tried and tried and tried to… I was on the sofa for four weeks without even really moving just to try and let the skin heal and seal and maximise its recovery. So that was tough but I still stayed positive all the way through but at the end, where I was… I think I went back on the 12th of September so June, July, I was probably two and a half months on those last couple of weeks was just- was an emotional struggle of just… just get to a stage where you can only stay positive so long (laughs) before I started to get a little bit down on the second staff infection, that it got to a stage where I was just thinking I've had enough of this now, I just want it to go away so I can get back on with day to day life.[NBT 012]

With burns, what you don't realise is that as horrific and as awful as it is, if you've got that mentality you won't let anything get in your way.[C&W 02]

It was clear that participants' mood states during recovery were not constant and many reported having periods where they felt very down.

But it is a long- you just don't… I have really down days where I think nothing's ever going to change and I get really frustrated. [NBT 013]

In the longer-term, there was variability among participants with respect to how well they were coping. Many participants reported that they were coping well emotionally. The end of active treatment and/or a return to normal functioning was sometimes associated with a more positive mood state.

Yeah, when he knew we didn't have to come back anymore he was really happy and the sleeping got a bit better, so he used to really panic about coming in 'cos it would hurt. [BCH 018]

.. once I got back to work and got back to the gym and all the day to day things, I'm totally fine with it now. Totally fine with it. [NBT 12]

Some participants described feeling more cautious or fearful of situations that were similar to those in which the injury occurred.

and I would test it like eight times and he will be really- won't get near anything if it's hot. He is a bit more over-cautious with stuff. Very obsessive but- So that might be a long-term thing that comes from it. [BCH 18]

Psychological impact on significant others (family and friends)

Many participants talked about the psychological impact that their injury had had on significant others. Family members often experienced distress at the time of the injury. Some parents expressed guilt that their child had been burned.

once we got to the medical tent we were just screaming so I just felt really guilty I must admit, that he'd burnt himself. And to be honest I should know better. Hot drinks. And I felt really bad 'cos I'm vegan so they had milk in a hot chocolate and I'd normally test but I didn't test it. [BCH 18]

The psychological impact on other family members continued beyond the short-term.

sleep-wise as well, (female sibling) was waking up in the night, obviously she's 11 so she normally sleeps fine, and she was waking up in the night and having nightmares and putting lights on and (son, patient) didn't sleep so between them both I was up every hour pretty much for weeks and then obviously I had panic attacks… it all went a bit…[BCH 018]

Several participants also discussed how family or friends had been a source of support to help them cope with what has happened to them.

my mates do the usual reminders of sending me stupid videos of people having vapes exploding in their pocket and this catching on fire because that's what blokes do don't they really? … and the canoeing trip that we went on, they had a playlist of everything, Come on Baby Light My Fire, its just everything but that's what blokes do. But that's their way of making sure, I know it sounds stupid, making sure I'm emotionally alright because if I'd had turned around and told them to turn it off or punch them in the face they would realise that I'm not taking it well at the moment so…[NBT 012]

Return to normal functioning/ability to perform daily activities

The desire to return to their normal levels of functioning was a very common theme amongst participants. In the short-term period immediately after the injury, this was often about being able to perform basic tasks of self-care. Participants described difficulties feeding themselves, moving around, using the bathroom. These activities were difficult to carry out because participants were unable to move the affected area or suffered too much pain when doing so. Tiredness and feeling weak were also identified as limiting factors.

particularly bathroom and (inaudible), I absolutely hated the bedpans but I was too weak to get out of bed- [C&W 06]

The eating was very difficult and I had to be fed through a tube which I really didn't like.

Yeah, I really didn't like being fed through a tube but then eventually when they took it out I was so weak I couldn't actually feed myself. [C&W 06]

Some participants were also worrying at this stage about being able to perform activities in the future such as being able to return to work or education.

I was concerned with my hands because firstly I work with my hands. These, all these three fingers, the skin was right off down to the raw bone, seriously raw flesh on this where it had all come off. And obviously both palms, you can't really see it now because they've healed so well but I was concerned with my hands to start off with because that is my livelihood.[NBT 012]

For patients who were hospitalised following their injury, the ability to be able to perform basic care tasks was also linked to being able to be discharged from hospital and return home.

like cooking, shopping and, yeah, so all the practical things, like (hospital) tried to help me with that before I left and then I went to (burns unit) and I had to live in a flat on my own but that never worked out 'cos somebody was in the apartment so I never actually (inaudible). So if I'm honest I didn't feel fully prepared at the time but anyway, that was (inaudible) it's just the everyday practical stuff and washing, that was difficult, getting in and out of the bath [C&W 06]

In the weeks following the injury, many participants still found that their injuries limited their abilities to perform basic tasks. Several participants described having to make adaptations, take extra time or have help in order to function.

For peer review only

I had to wake up at five thirty because getting dressed was really difficult for me. Although it's not too far, obviously back then my arms and my hands couldn't move as well as it could today, they were still very rigid sometimes, they didn't move very well and if I wanted to unbutton my shirt, 'cos the dress code was smart, if I wanted to button up my shirt it took me a good ten, fifteen minutes to work on the buttons [C&W 02]

I made- I had like an extendable backscratcher and I Sellotaped- I got a bit of plaster tape off the nurses (laughs) and Sellotaped my fork to it so I could-[NBT 08]

Yeah, (partner) would usually cut everything up and then I would have my scoop and- [NBT 08]

In the medium to long-term, participants described how their injures impacted upon their ability to take part in recreation or leisure activities. Some participants talked about how limited movement of the affected area or fear of causing damage to their healing wound restricted their ability to play sports. Swimming was also commonly identified by participants as an activity that they were not confident to do. Concerns about chlorine stinging or the visibility of their scars when in a swimsuit were mentioned.

At the moment I've got a friend who does some swimming, some aqua aerobic and I used to do some aqua aerobic with her but I don't think I would now because at first I was worried about chlorine in the water, but then after it's not just for that, it's as well the look of it.[NBT 007]

A lot of participants talked about how their burn limited their ability to go out in the sun. This impacted on playing sports, swimming and also, for some participants, their holiday choices. Several participants mentioned having been advised to keep the area covered to protect it from sun damage, but for some participants they were also motivated to keep the area covered in hot weather due to concerns about appearance.

Just occasionally, you know, like yesterday, I took the grandchildren to the park, finding a seat that wasn't in the sun because I'm anxious- if I feel the sun on it I feel anxious because I've been told at least a year but it's going to be more than a year 'cos its obviously it is more than a year.[C&W 005]

You need to wear sunscreen wherever you go and now I have to wear double sunscreen not only for the burns 'cos it can burn but like 'cos of the discoid lupus and vitiligo could get worse. You can't go to beaches, you can't go out in the heat, your body can't tolerate certain things.[C&W 02]

Moving into the long-term, some participants talked about how they felt they coped much better psychologically once they were able to return to their normal activities. For others, the extent of their injury meant that they would never return to their pre-injury level of functioning and this was harder to accept.

so, you know, four, five months ago but over the last two or three months I've really been just back in normal life and probably the things I had decided are important have now disappeared into the (inaudible) and the normal functioning life and its less mulling over existential questions and just getting on with the day to day tasks. [C&W 04]

I think what troubles me now I've got carers and that means I've got to go and ask them and I've never been a person- [NBT 10]

Physiological effects of burn or treatment

Temperature – several participants reported feeling too hot in hospital in the short-term. Temperature problems were an issue in the longer term for a few patients. Two reported rapidly changing temperatures.

For one participant, the loss of sweat glands has caused long-term permanent issues with temperature control.

Thirst – feelings of thirst and dehydration were reported by several hospitalised participants.

Tiredness – tiredness an issue in the short-term but persisted beyond that for some interviewees.

Nutrition – Several participants mentioned that staff had impressed upon them the importance of eating to help with their short-term recovery. They also described issues they had with using feeding tubes or a lack of appetite. In the longer-term, one participant reported having lost a lot of weight and a second described speaking with a nutritionist to get advice regarding optimum nutrition for healing.

Length of hospital stay

Participants whose injuries necessitated a stay in hospital expressed differing views about the length of their stay. Some participants found it difficult and expressed the desire to go home.

> *For me I would- the thing that brought me down the most was having to be in the hospital- [NBT 08]*

> *Oh no, not that I wanted to get out of the hospital but it's not nice being- I mean they're very good, I mean I couldn't have been in a better place, they're absolutely amazing but you do don't you naturally want to go home? [NBT 014]*

One participant described how she refused to be admitted and insisted on receiving treatment (antibiotics) at home instead.

> *so they wanted to keep me in the hospital but I refused and I managed ok at home 'cos of antibiotics at home because I didn't want to stop in the hospital. But my first initial thing here I was upset because they wanted to keep me in hospital. [NBT 019]*

For others, the length of hospital stay felt just right or was experienced as a positive.

> *It didn't really bother me particularly because I had all the- I just slept and I was feeling so ill about it all and, um, and I just chatted to visitors and stuff like that but I think if I was in any longer I would have gone a bit stir crazy. I was getting to the point where I was like no I just need to go home now. [NBT 002]*

> *Like being in hospital I found, even though it's kind of a long time and everyone is kind of like I want to go home, I think being in hospital a long time is handy. I don't know if (inaudible) but I wouldn't have wanted to go home any earlier.[BCH F.W.]*

For peer review only

2. Interviews with professionals

Initial treatment, preventing infection, promoting healing and reduce scarring

Interviewed staff members mentioned a range of factors that are crucial around the time patients are admitted to hospital. Staff discussed reasons for importance of these factors which can jointly be seen as a chain reaction of events where each is an important influencing factor for the next.

To begin with, staff involved in the treatment of smaller burns were mostly worried about adequate management of the injury and caring for the wound during the very acute phase. This included a quick and smooth process of assessment, cleaning, scrubbing and dressing.

> "[…] they need initial scrubbing, whether that be in theatre or in the ward, and getting them dressed up so that's the initial phase I would say is only to do acutely without thinking anything else." [BCH012]

Further prompting revealed that the underlying reason is the prevention of infection. Infections were mentioned by the majority of staff as the most important concern at the point of admission to hospital.

> "For infection, just to make sure there's no infection. If there's a lot of dead skin hanging around or blisters aren't de-roofed you could potentially get an infection just in the fluid that's still around or the dead skin." [BCH001]

Staff often mentioned that the prevention is important to avoid prolonging the healing process. Healing was seen as an important outcome, especially in connection with smaller burns.

> "[…]or that they do get a small infection, you know, the process is delayed then isn't it and the healing time is prolonged" [MH001]

> "[…] quite often we're very focused on dealing with the physical aspects of the burn injury, so making sure the burn wound heals, preventing infection. […] we're very focused on managing that wound and […] making sure the wound heals […]. [BCH014]

> "Infection is what we keep an eye out for and that the right dressings are going on to maximise healing." [BCH013]

> "[…] having them being cleaned and assessed and then you kind of do think about the healing as well because they're smaller, they'll heal within two weeks and then you kind of do think more about the healing […]" [BCH012]

Whilst scarring was not important for staff at the acute stage of the injury, it was often mentioned by staff as one of patients' major concerns. Participant BCH014 described the whole chain of events nicely:

> "You know, or if you're going along that journey of the wound healing, you know, sometimes you can come into problems like infection, for example. If there's anything delaying healing, then scarring can obviously still happen. […] And that's something that is important to us is dressing the wound in a way that will kind of protect the wound [...] [a]nd help it heal quickly." [BCH014]

Pain and distress management

Almost all staff considered pain management a vital part during the acute phase of injury treatment. For smaller burns a similar chain reaction of events was observed, where the underlying reason to control pain was to reduce the distress that patients and their carers were subject to.

> "Make things worse in time... in terms of like dealing with their pain management, can be quite distressing for the family. So I think feelings of, yeah, distress for the parent and in terms of managing that child's pain is probably the most paramount when they first see us.

> […] I've... remember once I had one parent who did panic when I was cleaning the wound, and wasn't quite expecting for me to be removing skin. So she felt as though I was doing something that I perhaps shouldn't..." [BCH014]

Further prompting elicited that reduced psychological trauma was a key motivation to manage the distress.

For peer review only

"Because being burnt is painful and we scrub the wound and take the blisters off which is very painful. And the children need to come back for repeat dressing changes so we need to make the first one as good as it can be. It's never nice but we need to make it as well managed and painless as possible." [BCH013]

Being in control

A small number of staff mentioned that feeling in control of the procedural steps and the situation is most important to them to ensure a high standard of burns treatment delivery. Participants mentioned the importance of underlying factors such as being well prepared for the treatment or presence of a well-functioning team.

"It's being in control of that procedure I feel as a clinical professional, making sure that, yeah, that if you lose- I think once you've lost the control of the procedure in some way then you've lost the parent confidence in you." [*BCH006]*

Other consequences of a potential lack of control were acknowledged by two staff members. These included personal impacts on self-confidence, patients' confidence in the treatment and potential further causes of stress. In this sense, this theme may be linked to previous findings where interviewees mentioned that a well-managed procedure also contributes to reducing distress (see also quote from BCH013 above).

"You don't want them to leave distressed and overwhelmed by it all. And then in the future you know that they're not going to mind so much coming back for future dressings so it just helps" [BCH001]

Survival

Interestingly, only two members of staff (BCH007, NBT006) mentioned survival as the most important outcome and BCH007 noted "*the most important outcome is survival because there are those patients that are very poorly and you just want them to survive*". This is only applicable for severe and larger body surface burns.

Facilitate independence from healthcare

Following discharge, it was important to the majority of staff to help patients gain independence from the care of the hospital. Several sub-themes emerged that could contribute to facilitating independence.

Self-management of wound

A major concern for staff was that patients are able to manage their wound when returning home. This included continued appropriate wound care to prevent further scarring and getting the wound to "a normal state" [MH002].

"I would say our main concern is will the families be able to continue with the treatment that we've taught them. So, it might be creaming and massage […] and protecting it from the sun." [BCH30102019]

"[…] making sure that we instruct them […] on the massage and moisturisation at home, which they should be doing several times a day to kind of help." [BCH014]

This included the self-efficacy and confidence to carry out the wound treatment and providing the tools and information to carry it out in the right way (e.g. how to do the dressings, cleaning etc).

I guess that they feel equipped and empowered to be the main carer for their child [BCH006]

For peer review only

to take ownership of their own wounds from the outset so that they can manage their own lives 'cos of course they've come here, you have these rather complicated looking dressings put on and everything looks kind of amazingly difficult and complicated whereas commonly it's really straightforward and a lot of what we do isn't necessarily super sterile, its clean and these are things often times that can be managed very well at home with a bit of support from family [MH001]

Patient knowledge and understanding

At the point of discharge, it was considered critical that patients are given the advice needed so they would be *"well equipped with the information that they need" [BCH006]* to help patient understanding of the wound and treatment. This encompassed process-related information related to the self-management of the wound as described above.

"It's explaining to them what they need to be doing to achieve this wound to heal, and like I say before, being honest about it" [NBT026]

I think that's quite important to pass that on and advice about sun protection is really important because it's new skin so that they need to be advised about that as well. [BCH008]

Staff repeatedly mentioned it was necessary to ensure that patients understand signs of infection and closely monitor the wound for e.g. redness, swelling or changes of the wound and starting to feel unwell.

"It's just knowing that the patient understands how to look after their wounds, and to look out for signs of infection […] if they start [t]o feel unwell and it's their understanding of that really" [NBT026].

It was vital for staff to emphasise that patients' understanding includes the management of their expectations with regards to the outcome of their injury.

Knowledge of availability of support

After discharge, staff frequently mentioned it was important to them to communicate the constant support available to patients and when to make use of it.

"My biggest goal and concern for them is can they access everything they want to access." [BCH007]

"Identify when they need help and be able to just call us and we would say come back straight away if there's a problem" [BCH006]

Independence from healthcare was less of a priority as time progressed, however, availability of psychological support was mentioned as important in the long-term and is further described in the 'Psychology' theme.

Discomfort during healing

Concerns about contractures and the desire to improve any tightness of the skin were common concerns for larger burns.

"So giving them a contractual release to allow them to return to as normal a state as possible, even if it's not specifically function in terms of what they do but how they can stand, how they can hold their heads" [NBT001]

A minority of staff were concerned about patients' pain control after discharge or experiencing itching.

"When they heal can get quite itchy, so by putting the moisturiser on, keeping it... you know, preventing it from drying, scabbing, you are going to help that itchiness." [BCH014]

For peer review only

"We want them to go home pain-free, or very near pain-free, where they're able to control their pain at home". [NBT026]

Other interviewees had different opinions and focused solely on self-management of the wound:

"I mean that's the only real priority there is then because they shouldn't be in any pain, they're not going to need any further follow-up so it's just making sure that they manage the healed wound" [BCH006]

Psychology

A large number of interviewees mentioned the importance of potential psychological impacts in the long-term.

"I mean long-term the important outcomes really are how they are psychologically" [BCH30102019].

"I suppose what I'm saying is the psychology, […] can be long-term, so we would be perhaps seeing families where could be a long time previous that the injury… when their children were toddlers and then they perhaps go into puberty and if they've had chest scarring or whatever or where teenagers are wanting to wear strappy tops or bikinis or proms, that kind of thing, it all floods back and that's when could be years ago that they had the actual burn but we would then perhaps get involved." [BCHRP30102019]

"I think their general wellbeing and happiness is also a long-term goal." [BCH007]

"In some, you know, the injuries that had really knocked someone for six and kind of really turned their lives upside down in the process." [NBT0004]

Psychological wellbeing and impact of injury & scarring

In particular, concerns were raised most frequently in connection with how the injury happened or the appearance of the scar (cosmesis). Probing revealed several aspects affecting the psychological wellbeing. Specifically, underlying internal (e.g. self-esteem, acceptance and normalisation of the injury) and external influences (e.g. bullying, worry related to intimate relationships and other people's views) were mentioned by staff.

"I would say cosmesis is the most one 'cos burns can be disfiguring, they can leave scars which are not very nice and not very slightly, so it affects patients psychologically and in kind of their social groups as well and also the parents as well." [BCH012].

"I'd be still cautious about psychology and how they've coped, especially if it is going to scar, what impact that's going to have on their self-esteem and bullying potentially at the school." [BCH013]

"Some people cope with it, some people don't. Everybody's different, and just giving them coping mechanisms for the community really." [NBT026]

"Definitely anxiety… yeah. Just the whole impact of the injury, I think it's just frightens them in many ways." [BCH008]

In particular, staff thought it was important that psychological support was available in the long-term which recognised signs of PTSD (e.g. sleeping problems, flashbacks and impacts on relationships).

[…] that psychologically they're supported."

For peer review only

"I suppose it's some sort of way of keeping an eye on people without being intrusive so that if stuff emerges down the line [...] It's about, A, if they're still getting post traumatic symptoms and, B, the impact it's having on them and if, for example, [...] they're frightened to go to sleep or it's affecting their relationship or whatever then you'd start to pay more attention." [NBT022]

Impact on families and daily activities

Professionals often mentioned the psychological impact of the burn on daily activities.

"[...] even if they haven't got scarring but then just integrating back into going to school, that might be hard so that could impact their lives that way." [BCH30102019]

When the injury involved children, staff highlighted the added psychological impact on parents and the family as a whole.

"So I think there's a huge psychological aspect on all the patients and actually the mobility and the functional side is only the more severe side of a patient 'cos obviously the less injured patients will get better very quickly from the function and mobility point of view and it's never a problem. So I think there's, yeah, psychological with all of them and mobility and function with most of them I'd say." [BCH007]

"it depends how the injury happened, if there's any like psychological impact on either the child... I mean, quite often the children we see are under the age of five and they don't often remember what happened. But there can be quite big like load of guilt on either the parent... if it involves the parent or quite often involves grandparents who have put, you know, a cup of coffee on the side and the child has grabbed it and sort of tipped it down themselves, if it's created... if it caused a large burn and a large injury, and they had quite a big treatment period in hospital, then that person, the grandparent or the parent, can have a lot of guilt and a lot of distress around that situation. So quite often it's, I guess, dealing with that." [BCH014]

Functional impacts

Impacts of the injury (or scar) on how it affects patients returning to normal function was an important concern for staff after discharge and in the long-run.

"Well to be living as normal a functioning, happy life as possible after having a burn injury." [BCH006]

"Function essentially, yeah [...] Returning to their pre-burn level of function." [MH003]

Return to normal activities

One of the most important outcomes for staff during the medium-term (for smaller burns) and in the long-term (for larger burns) was that patients can return to their normal activities. This predominantly included the ability to return to work and school, and carry out day-to-day tasks and hobbies or other leisure activities.

"It's just encourage them just to get back into normal life now, just carry on things as normal." [BCH008]

"The most important outcome is, for me personally and professionally, it's their physical outcome. So it's are they walking, are they active, are they engaging in every activity that they would want to engage in." [BCH007]

"So initially I think it's kind of their return to their sort of family roles and their normal activities, when can I go back to work, when can I do my leisure activities." [MH002]

For peer review only

Movement restrictions and contractures

Staff directly involved in the treatment of burns affecting relevant areas (OT, Physios) were worried about the impact of the wound on movement.

> *"[…] hoping that the burn isn't affecting them anymore and that it is all settled down and, yeah, just living a normal life. [BCH008]*

> *"[…] the focus is very strongly on functionality. In other words my scar is preventing me from doing this, can you sort my scar out." [NBT001]*

> *"I would say mainly functional is the later one. […] I think the ultimate aim is them to be not functionally restricted with the scars and the scar that is down and not very angry and almost back to normal skin. " [BCH012]*

> *"Long-term would be the scarring and whether contracture is affecting movement" [BCH013]*

Specific location of burns were frequently mentioned as requiring additional attention because they affect mobility.

> *just being mindful [...] that [...] if the burn injury went over a certain area, so if it goes over a joint, sometimes we might book […] an appointment with the OTs anyway, like three months down the line.[BCH014]*

> *"And then… especially around joints, so they lose their mobility of their joint as well, and it may cause… maybe, you know, they've been quite into their sport, and that may impact, so they… they can't any longer play the sport that they were doing before, or maybe the job…" [NBT026]*

Scarring and continued scar management

A commonly mentioned important long-term concern for staff was the level of scarring and associated treatments to manage it.

> *"I think scarring probably is like the biggest." [BCH014]*

Worry about scarring was twofold: staff were concerned about the functional impacts in the medium to long-term as well as the appearance of the scar.

> *"I think the ultimate aim is them to be not functionally restricted with the scars and the scar that is down and not very angry and almost back to normal skin" [BCH012]*

Concerns about the appearance were determined by patients' perception and worry about scarring, linked to potential psychological impacts.

> *"From a clinician's point of view it's a great outcome, her scar wasn't lumpy or bumpy, there's not really much there that we can see but from their point of view it affects them and how they cope, especially in teenage years as well." [BCH012]*

For larger burns, getting the wounds to "their optimum" [BCH30102019] is a priority for staff involved in the ongoing longer-term management of scars. This includes the appropriate treatment under the scar management path, e.g. pressure garments or surgery to "just get[…] the best outcome from that injury that you can." [BCH30102019]

> *"scarring, needing scar management help, perhaps like pressure garments or steroid creams" [BCH001]*

For peer review only

"So in terms of therapies by scar management teams and maybe pressure garments, silicones, that sort of thing, and then ultimately potentially like steroid injections or some form of surgery […]." [BCHRP30102019]

Psychological effects of the continued and potentially years-long treatment were also acknowledged by staff.

"So, not only do they have to get over that initial situation, they have to live with their… their scars and ongoing surgery, and things like that. So some people it's lifelong." [NBT026]

"The size of the injury is important to a certain extent and the amount of scarring someone's going to have and the potential reconstructive surgery because of course we're potentially re-traumatising people or people are having new things that they've got to kind of cope with." [MH002]

"Some children have a really tough journey in that way, for years, who are under scar management, under therapy for repeated […] reconstructive surgery [BCH006].

3. Combining themes

Information from the descriptive report were compared and contrasted and eventually condensed into combined themes. During this process, decisions were made to combine, separate or discard themes and suitable labels were agreed in team discussions. Tables S4 and S5 list the identified themes, how these are linked to combined themes and which outcome domains could be mapped to themes' conceptual content.

Table S4: Patient interviews themes, combined themes, and mapped outcome domain

**Themes (sub-themes)**	**Combined themes**	**Outcome domains (outcome category) as per outcome classification by Young et al (2019)**
Scarring	Scarring	• Scar texture (scar-related)<br>• Scar colour (scar-related)<br>• Scar size (scar-related)<br>• Appearance (patient-reported)
Pain	Pain and discomfort	• Burn wound pain (patient-reported)<br>• Pain during procedures (patient-reported)<br>• Itch (patient-reported)<br>• Scar pain (patient-reported)<br>• Effect of scar on movement [contractures] (patient-reported)<br>• Comfort of dressings (patient-reported)
Tightness		
Itching		
Infection	Infection	• Burn wound infection (complications)<br>• Sepsis (complications)
Appearance of the wound	Healing	• Burn wound healing (pathophysiological)<br>• Appearance (patient-reported)
Time to healing		
Psychological effects (Psychological impact on significant others [family and friends])	Psychological wellbeing	• Psychological well-being (patient-reported)<br>• Generalised anxiety (patient-reported)<br>• Mental ability (patient-reported)<br>• Quality and quantity of sleep (patient-reported)

For peer review only

Return to normal functioning/ability to perform daily activities	Function	• Ability to carry out daily tasks (patient-reported)<br>• Return to work/school or previous function (patient-reported)<br>• Appearance (patient-reported)
Uncertainty, lack of knowledge and the need for information	Uncertainty	None identified
Length of hospital stay	Discarded	
Physiological effects of burn or treatment	Discarded	

Table S5: Professional interviews themes, combined themes, and mapped outcome domain

**Themes (sub-themes)**	**Combined themes**	**Outcome domains (outcome category) as per outcome taxonomy**
Initial treatment, preventing infection, promoting healing and reduce scarring	Healing	• Burn wound healing (pathophysiological)
	Infection	• Burn wound infection (complications)
Pain and distress management	Pain and discomfort	• Use of medicines to treat symptoms (healthcare-related)<br>• Burn wound pain (patient-reported)<br>• Effect of scar on movement [contractures] (patient-reported)
Discomfort during healing		
Scarring and continued scar management	Scarring	• Treatment for scars (scar-related)<br>• Scar texture (scar-related)<br>• Scar colour (scar-related)<br>• Scar size (scar-related)<br>• Appearance (patient-reported)
Functional impacts (Return to normal, Movement restrictions)	Function	• Mobility (pathophysiological)<br>• Ability to carry out daily tasks (patient-reported)<br>• Return to work/school or previous function (patient-reported)
Psychology (Psychological wellbeing and impact of injury & scarring, Impact on families and daily activities)	Psychological wellbeing	• Psychological well-being (patient-reported)<br>• Generalised anxiety (patient-reported)<br>• Mental ability (patient-reported)<br>• Quality and quantity of sleep (patient-reported)
Survival	Survival	• Death from burn injury (complications)<br>• Death from any cause (complications)
Being in control	Sense of control	None identified
Facilitate independence from healthcare (Self-management of wound/ independence, Patient knowledge and understanding, Knowledge of availability of support)	Patient knowledge, understanding & support	None identified

For peer review only

Standards for Reporting Qualitative Research (SRQR)*

http://www.equator-network.org/reporting-guidelines/srqr/

Title and abstract

	Page/line no(s).
**Title** - Concise description of the nature and topic of the study Identifying the study as qualitative or indicating the approach (e.g., ethnography, grounded theory) or data collection methods (e.g., interview, focus group) is recommended	1
**Abstract** - Summary of key elements of the study using the abstract format of the intended publication; typically includes background, purpose, methods, results, and conclusions	2

Introduction

**Problem formulation** - Description and significance of the problem/phenomenon studied; review of relevant theory and empirical work; problem statement	4
**Purpose or research question** - Purpose of the study and specific objectives or questions	4

Methods

**Qualitative approach and research paradigm** - Qualitative approach (e.g., ethnography, grounded theory, case study, phenomenology, narrative research) and guiding theory if appropriate; identifying the research paradigm (e.g., postpositivist, constructivist/ interpretivist) is also recommended; rationale**	5
**Researcher characteristics and reflexivity** - Researchers' characteristics that may influence the research, including personal attributes, qualifications/experience, relationship with participants, assumptions, and/or presuppositions; potential or actual interaction between researchers' characteristics and the research questions, approach, methods, results, and/or transferability	5-6, 16
**Context** - Setting/site and salient contextual factors; rationale**	2, 5
**Sampling strategy** - How and why research participants, documents, or events were selected; criteria for deciding when no further sampling was necessary (e.g., sampling saturation); rationale**	5
**Ethical issues pertaining to human subjects** - Documentation of approval by an appropriate ethics review board and participant consent, or explanation for lack thereof; other confidentiality and data security issues	5, 20-21
**Data collection methods** - Types of data collected; details of data collection procedures including (as appropriate) start and stop dates of data collection and analysis, iterative process, triangulation of sources/methods, and modification of procedures in response to evolving study findings; rationale**	6

For peer review only - http://bmjopen.bmj.com/site/about/guidelines.xhtml

**Data collection instruments and technologies** - Description of instruments (e.g., interview guides, questionnaires) and devices (e.g., audio recorders) used for data collection; if/how the instrument(s) changed over the course of the study	6
**Units of study** - Number and relevant characteristics of participants, documents, or events included in the study; level of participation (could be reported in results)	7-8
**Data processing** - Methods for processing data prior to and during analysis, including transcription, data entry, data management and security, verification of data integrity, data coding, and anonymization/de-identification of excerpts	6-7
**Data analysis** - Process by which inferences, themes, etc., were identified and developed, including the researchers involved in data analysis; usually references a specific paradigm or approach; rationale**	6-7
**Techniques to enhance trustworthiness** - Techniques to enhance trustworthiness and credibility of data analysis (e.g., member checking, audit trail, triangulation); rationale**	5-7

Results/findings

**Synthesis and interpretation** - Main findings (e.g., interpretations, inferences, and themes); might include development of a theory or model, or integration with prior research or theory	9
**Links to empirical data** - Evidence (e.g., quotes, field notes, text excerpts, photographs) to substantiate analytic findings	9-14

Discussion

**Integration with prior work, implications, transferability, and contribution(s) to the field -** Short summary of main findings; explanation of how findings and conclusions connect to, support, elaborate on, or challenge conclusions of earlier scholarship; discussion of scope of application/generalizability; identification of unique contribution(s) to scholarship in a discipline or field	15-16
**Limitations** - Trustworthiness and limitations of findings	16

Other

**Conflicts of interest** - Potential sources of influence or perceived influence on study conduct and conclusions; how these were managed	20
**Funding** - Sources of funding and other support; role of funders in data collection, interpretation, and reporting	20

*The authors created the SRQR by searching the literature to identify guidelines, reporting standards, and critical appraisal criteria for qualitative research; reviewing the reference lists of retrieved sources; and contacting experts to gain feedback. The SRQR aims to improve the transparency of all aspects of qualitative research by providing clear standards for reporting qualitative research.

For peer review only

**The rationale should briefly discuss the justification for choosing that theory, approach, method, or technique rather than other options available, the assumptions and limitations implicit in those choices, and how those choices influence study conclusions and transferability. As appropriate, the rationale for several items might be discussed together.

Reference:
O'Brien BC, Harris IB, Beckman TJ, Reed DA, Cook DA. **Standards for reporting qualitative research: a synthesis of recommendations.** *Academic Medicine*, Vol. 89, No. 9 / Sept 2014 DOI: 10.1097/ACM.0000000000000388

For peer review only

For peer review only - http://bmjopen.bmj.com/site/about/guidelines.xhtml

BMJ Open

Exploring what is important during burn recovery: A qualitative study investigating priorities of patients and healthcare professionals over time

Journal:	*BMJ Open*
Manuscript ID	bmjopen-2021-059528.R3
Article Type:	Original research
Date Submitted by the Author:	19-Jan-2023
Complete List of Authors:	Hoffmann, Christin; University of Bristol, Population Health Sciences<br>Davies, Philippa ; University of Bristol, Population Health Sciences<br>Elliott, Daisy; University of Bristol, Population Health Sciences<br>Young, Amber; University of Bristol, Population Health Sciences; University Hospitals Bristol and Weston NHS Foundation Trust
**Primary Subject Heading**:	Qualitative research
Secondary Subject Heading:	Patient-centred medicine, Health services research
Keywords:	Quality in health care < HEALTH SERVICES ADMINISTRATION & MANAGEMENT, QUALITATIVE RESEARCH, Clinical trials < THERAPEUTICS

SCHOLARONE™
Manuscripts

I, the Submitting Author has the right to grant and does grant on behalf of all authors of the Work (as defined in the below author licence), an exclusive licence and/or a non-exclusive licence for contributions from authors who are: i) UK Crown employees; ii) where BMJ has agreed a CC-BY licence shall apply, and/or iii) in accordance with the terms applicable for US Federal Government officers or employees acting as part of their official duties; on a worldwide, perpetual, irrevocable, royalty-free basis to BMJ Publishing Group Ltd ("BMJ") its licensees and where the relevant Journal is co-owned by BMJ to the co-owners of the Journal, to publish the Work in this journal and any other BMJ products and to exploit all rights, as set out in our licence.

The Submitting Author accepts and understands that any supply made under these terms is made by BMJ to the Submitting Author unless you are acting as an employee on behalf of your employer or a postgraduate student of an affiliated institution which is paying any applicable article publishing charge ("APC") for Open Access articles. Where the Submitting Author wishes to make the Work available on an Open Access basis (and intends to pay the relevant APC), the terms of reuse of such Open Access shall be governed by a Creative Commons licence – details of these licences and which Creative Commons licence will apply to this Work are set out in our licence referred to above.

Other than as permitted in any relevant BMJ Author's Self Archiving Policies, I confirm this Work has not been accepted for publication elsewhere, is not being considered for publication elsewhere and does not duplicate material already published. I confirm all authors consent to publication of this Work and authorise the granting of this licence.

For peer review only

Exploring what is important during burn recovery: A qualitative study investigating priorities of patients and healthcare professionals over time

Submission category: Original research

Authors:

C Hoffmann[1], c.hoffmann@bristol.ac.uk

P Davies[1], Philippa.Davies@bristol.ac.uk

D Elliott[1], daisy.elliott@bristol.ac.uk

A E Young[1,2,*]

Author affiliations:

[1] National Institute for Health Research Bristol Biomedical Research Centre, Bristol Centre for Surgical Research, Bristol Medical School: Population Health Sciences, University of Bristol, 39 Whatley Road, Clifton, Bristol, BS8 2PS, UK

[2] Bristol Royal Hospital for Children, University Hospitals Bristol and Weston NHS Foundation Trust, Upper Maudlin St, Bristol BS2 8BJ

*The author sadly passed away on 17 September 2022. The paper was revised and resubmitted for publication by the corresponding author.

Correspondence: Christin Hoffmann [c.hoffmann@bristol.ac.uk]

Running title: PRIORITIES OF BURNS PATIENTS AND PROFESSIONALS OVER TIME

Keywords: Burns; Qualitative; Outcomes; Outcome measurement

Word count: 4993 (excluding title page, abstract, tables and references)

For peer review only - http://bmjopen.bmj.com/site/about/guidelines.xhtml

Abstract

Objectives: This qualitative study aimed to investigate: i) priorities of patients and healthcare professionals during recovery from a burn injury, ii) how priorities change over time, and ii) how priorities map to outcomes currently reported in burns research.

Design: Semi-structured interviews were conducted. Interviews were audio recorded, transcribed and analysed thematically.

Setting, participants: A total of 53 patients and healthcare professionals were recruited from four National Health Service (NHS) burn services across England and Wales across England and Wales. Patient participants (n=32) included adults, adolescents, and parents of paediatric patients, with a variety of burn injuries in terms of severity and cause of burn injury. Healthcare professionals (n=21) were NHS staff members involved in burn care and included professionals with a range of clinical experience and roles (e.g. nurses, surgeons, occupational therapists, physiotherapist, administration).

Results: Ten themes relating to priorities (outcomes) during recovery from a burn injury were identified for patients and professionals. Of those, six were identified for patients and professionals ('pain and discomfort', 'psychological wellbeing', 'healing', 'scarring', 'function', 'infection'), three were unique to professionals ('patient knowledge, understanding & support', 'sense of control', 'survival') and one was unique to patients ('uncertainty'). Results highlighted that importance of these priorities changes over time (e.g. 'survival' was only a concern in the short term). Likewise, priorities differed between patients and professionals (e.g. 'pain' was important to patients throughout their recovery, but not for professionals). Seven out of ten themes overlapped with outcomes commonly assessed in burn research.

Conclusion: Professionals' and patients' priorities (important outcomes) change over time after burn injury and differ between those groups. Burn care research should consider measuring outcomes at different time points during the recovery from a burn injury to accurately reflect complexity of burn recovery.

(287 words)

For peer review only - http://bmjopen.bmj.com/site/about/guidelines.xhtml

Strengths and limitations of this study

- A diverse sample of professionals and patients were recruited from four NHS burn services across England and Wales to elicit a wide range of perspectives.
- The study employed semi-structured interviews with a large sample of 52 participants
- Multiple layers of analyses were applied, involving thematic analysis, comparison of views between patients and professionals, and mapping themes to outcomes
- Identified themes are broadly relevant to patients with all types of burn injuries, but a more homogeneous sample of burn types may provide more detailed and nuanced insights into priorities of specific groups of patients.

For peer review only

For peer review only - http://bmjopen.bmj.com/site/about/guidelines.xhtml

INTRODUCTION

Each year, approximately 10,000 patients with burn injuries are treated in the United Kingdom National Health Service (NHS) burn services [1]. Burns can affect many aspects of patients' lives, including their function, cosmesis and psychological well-being. Injury management incurs challenges due to their varying complexity (e.g. burn size and location) and factors unique to patients' personal circumstances (e.g. age, profession) [2]. On-going development of treatments to improve outcomes (e.g. quality of life, scarring) means that evidence for optimal strategies for treating burns is still evolving [3]. Accurate measurement of patient relevant outcomes to evaluate strategies is key to achieving high-quality, patient-centred care [3,4].

The importance of relating patients' perceptions to measurable patient-relevant outcomes in burn research has been recognised [5–7]. Burns can impact affected individuals and their families in the short and in the long term in different ways [8]. For larger burns, for example, survival can be the main priority in the acute stages of treatment, whilst scarring may be a long-term concern. Similarly, scarring may be more or less important for some population groups and professionals involved their care [9]. It is probable that perceptions about what matters most to patients and professionals may vary between those groups and over time. Consequently, the importance of outcomes may also vary. Current methods in burns research, however, commonly assume equal importance of outcomes at a specific time for all stakeholders [10]. Measuring a certain outcome at a certain time might not, however, provide a true picture of patients' recovery, and decisions about optimal treatment may therefore be ill informed. It is unclear how specific priorities differ across time as well as between patient and professionals as recovery from a burn injury progresses.

There is a need to identify which priorities are important at which time points during burns recovery. This may facilitate optimisation of outcome measurement and ensure evaluation of treatment strategies is patient-centred. Qualitative research is important in burns care [11] and has been widely used to explore perspectives and concepts important to burns survivors [3,6,12]. Previous work has shown that (mostly objective) clinical outcomes thought to be important, may not align with patients' views and perspectives [13]. A large number of qualitative studies have been conducted to explore concepts important to burns survivors only [6]. Likewise, outcome taxonomies have been proposed in this area and include patient-reported and patient-relevant concepts [14,15]. Less attention, however, has been paid to exploring professionals' views and literature often focusses on specific aspects of the recovery, such as scarring characteristics [15]. It is therefore important to understand professionals' *and* patients' priorities during burn recovery as both need to

For peer review only - http://bmjopen.bmj.com/site/about/guidelines.xhtml

play a role in making decisions about treatment and care [16,17]. Likewise, understanding how they are linked to commonly measured outcomes in burn care research may guide future study design.

Aim

This qualitative study had three objectives:

i. Investigate priorities of burns patients (including adults, adolescents and parents of paediatric patients) and healthcare professionals during burn recovery and explore how they differ.
ii. Explore how priorities of patients and healthcare professionals change over time.
iii. Map identified themes to outcomes currently reported in burns research.

METHODS

This study uses qualitative methods, employing in-depth, semi-structured interviews to explore participants' perspectives over time.

Conduct and reporting of this study adhered to standards for reporting qualitative research (SRQR) [18]. This study adopted an interpretivist approach to understand participants perspective, because it is acknowledged that experiences of all burn survivors are unique and that individuals' perceptions are construed within their own particular setting which may implicate their families and social networks [19].

Setting and participants

This study recruited two groups of participants (patients and healthcare professionals) from four NHS burn service across England and Wales. Three were adult services and one specialised in paediatric burns. Patient participants were NHS patients with lived experience of burn injuries. These included adults (of 16 years and older), adolescents (10-15 years of age) and parents of paediatric patients (of less than ten years of age) (henceforth simplified to 'patients'). Patients with varying injury severity and burn sizes were eligible. Interviews were conducted at least 30 days after injury and/or with patients who were not in acute treatment (e.g. larger burns). This ensured that all participants were able to reflect on their experiences around the time when the injury happened and during acute treatment, and that they were also able to articulate what is most important to them when in the following stages of recovery. Healthcare professional participants were

For peer review only - http://bmjopen.bmj.com/site/about/guidelines.xhtml

multidisciplinary NHS employees of burn services at the selected study sites (henceforth simplified to 'professionals').

Eligible participants were approached by research nurses or clinical / academic researchers (KC, AS, CS, AY) and invited to participate in the study. Information about the study was provided and any questions were answered before written consent was obtained. A clinical (AY) or academic (CH) researcher, both trained and experienced in qualitative research, contacted participants to arrange a suitable interview method, location, date and time.

Purposive sampling was employed to select participants with from a wide range of backgrounds [20]. Participant characteristics were reviewed regularly to ensure maximum possible variation between patients (e.g. age, gender, severity and cause of burn injury) and professionals (e.g. clinical experience, role). Recruitment continued until no new ideas and concepts evolved from the interviews (see "analysis" section for further detail on our approach for data saturation).

Data collection

Interviews with patients and professionals were completed face-to-face or over the phone, depending on participant preference. Face-to-face interviews were carried out in a quiet and private space in one of the four hospitals to ensure no interruptions or time limitations were impacting the interviews. In-depth interviews were conducted independently by two study members (AY, CH). AY is a consultant anaesthetist in paediatric burn care with 23 years of clinical experience and substantial experience of burns research. CH is a social scientist and qualitative researcher with no involvement in burns care or research prior to this study.

Interviews followed a semi-structured format using a topic guide with open-ended questions to guide discussions (see Supplemental file_Table S1 and Supplemental file_Table S2 for examples). Participants were asked to retrospectively reflect on their experience or priorities relating to different times after burn injury. There are no consensus definitions of time frames in burn care. To ensure consistency of explored perspectives, significant points during recovery from a burn injury were used as temporal anchors (e.g. point of discharge is considered a crucial point for outcome assessment [21]). For the purpose of this study, time periods were therefore defined *a priori* as: short term (immediately after the injury), medium term (from around the time of discharge and/or wound healing) and long term (during rehabilitation). An initial topic guide was developed based on the study objectives of exploring priorities retrospectively over time. This was piloted prior to study

For peer review only - http://bmjopen.bmj.com/site/about/guidelines.xhtml

commencement but allowed to evolve during the study whereby iterative refinements were made as data collection progressed.

Analysis

All interviews were audio-recorded using an encrypted device, and de-identified during transcription. Random samples of verbatim transcripts were checked for accuracy by one researcher (CH).

Analysis of qualitative data was undertaken using a qualitative data management software (NVivo Version 12). In a first step, transcripts were analysed using thematic analysis [22] to understand priorities of patients and professionals (study objective i). Interview transcripts were analysed separately for patients and professionals to accommodate concepts unique to each group and facilitate later examination of differences. This study used deductive and inductive approaches to coding [23]. Codes for short-, medium- and long-term priorities were developed *a priori*, to explore perspectives over time (study objective ii). *A priori* codes corresponded to time points (short term, medium term, long term) defined for the purpose of this study (described above). Additional codes were identified through line-by-line coding following principles of inductive theme development in line with six steps of thematic analysis [22]. Transcripts were read and re-read to allow familiarisation with the data. Initial codes were assigned from which themes were developed: Coded excerpts were grouped into themes of similar meaning and organised into a hierarchical structure of themes and sub-themes. This thematic structure was refined as new themes emerged. These were then reviewed and refined through discussions amongst the study team until final definitions and labels were agreed: This process involved iterative rounds of interpretation of contexts specific to the interviewees' injury and identification of relationships between themes. Diagrams illustrating themes and relationships were used to visualise the data and aid discussions between team members. In a second step, differences in priorities between patients and professionals were examined by comparing narrative accounts of each group. Connections were drawn between patients and professionals by qualitatively contrasting themes identified for each group to find similarities and differences in their perspectives (study objective i). This step also involved combining themes by condensing and relabelling which was shaped by the data and the team's experience and knowledge with defining, identifying and classifying outcomes in burns research.

Coding and analyses were undertaken independently by two researchers (CH, PD) who met regularly to discuss emerging themes from the data. Close contact between the coders during data analyses ensured consistency in coding and agreement of interpretation of the data. Independent double-

For peer review only

For peer review only - http://bmjopen.bmj.com/site/about/guidelines.xhtml

coding was also performed for 25% of transcripts by the two reviewing authors. A purposive sample of transcripts that underwent double-coding was used to ensure maximum variation by participant group, socio-demographic background and injury severity. Data from double-coding were compared and discussed to ensure consistency in approaches to further coding. Any uncertainties and discrepancies during this process were discussed with the wider study team and in consultation with an experienced qualitative researcher who was independent to the study team (DE). Interim interpretation and structure of themes was discussed with the wider study group and if necessary, iteratively modified.

Analyses were carried out in parallel to data collection to determine data saturation separately for both participant groups. This means, no new codes or meaning were identified through additional interviews and sufficient data was collected to address the research objectives [24–27]. Specifically, interim results during analyses were reviewed regularly by the study team and decisions of whether saturation was achieved were based on impressions of whether collected data provides sufficient conceptual depth [28,29]. If saturation was expected, a small number of additional interviews were conducted to confirm that no new codes or meaning were identified [30]. This process was overseen by the senior author (AY) who has a clinical background in burn care. A final thematic structure and meaning of themes were agreed in team discussions. Findings were collected in two descriptive accounts (one for patients, one for professionals) to be able to identify similarities and differences in key findings.

Themes identified through analyses described above were mapped against common outcomes in trials to assess whether perceptions explored in this work align with routinely measured outcomes in burns research (objective iii). This step involved comparing identified themes and their conceptual content to an existing outcome classification (see Supplemental file_Table S3) which was developed through synthesis of 955 unique outcomes reported across 147 randomised controlled trials in burn care [14].

This multi-layer analysis allowed to elicit priorities qualitatively from experiences of patients and professionals while linking experiential data to previously identified and classified outcomes.

Participant characteristics were summarised using descriptive statistics. Data related to burn size were grouped into categories (minor, moderate, major) according to percentage total body surface area (TBSA), following established classification guidelines [31].

For peer review only - http://bmjopen.bmj.com/site/about/guidelines.xhtml

Patient and Public Involvement

This study is part of a wider project that aims to standardise outcome reporting and measurement in studies of burns injuries. Patient representatives and carers were actively involved in the design, conduct, analysis and dissemination of aspects of the wider project.

RESULTS

Participant characteristics

A total of 53 participants (32 patient participants, 21 professional participants) took part in semi-structured interviews, lasting between 15 and 45 minutes (mean = 32 minutes). Four patient interviews were conducted with two participants (three with a young person and their parents, one included a patient and their carer). Participants were recruited from four NHS trusts between June 2017 and February 2020.

Patient participants' age ranged from 1 – 82 years (*Mean*=38; SD=27.4) and 19 (59%) participants were female. The sample mainly consisted of adults (n=20 participants, 65%). Scalds were the most common burn type (n=10, 36%). Participants primarily suffered from minor burns (n=14, 50%) with six (21%) patients who experienced major burns.

Healthcare professionals sampled were mostly female (n=17, 82%) with the majority of participants in a nursing role (n=11, 52%). Detailed participant characteristics are presented by participant group in Table 1.

Table 1 Participant characteristics (N=53)

	**Patients (N=32)**	**Professionals (N=21)**
**Age, mean (SD; range)**	38 (27.4; 1-82)	
**Female, n (%)**	19 (59)	17 (82)
**Occupation, n (%)**		
Consultant clinical psychologist		3 (14)
Physiotherapist		1 (5)
Consultant, registrar		2 (10)
Occupational therapist		3 (14)
Research, Specialist, Clinic or Senior nurse		11 (52)
Administration		1 (5)
**Patient type, n (%)**		
Adult (16 years and older)	20 (63)	
Young person (10-15 years)	3 (9)	

For peer review only - http://bmjopen.bmj.com/site/about/guidelines.xhtml

Parent (of child aged <10 years)	6 (19)
Parent (of young person aged 10-15)	3 (9)
**Burn size, n (%)***	
Minor (<10% TBSA)	14 (50)
Moderate (10-20% TBSA)	2 (7)
Major (>20% TBSA)	6 (21)
Not reported	6 (21)
**Burn reason, n (%)***	
Scald	10 (36)
Flame	8 (29)
Chemical	3 (11)
Flash	3 (11)
Contact	2(7)
Electric	1 (4)
Not reported	1 (4)

** % data based on number of patient interviews (n=28) because this reflects the number of injuries*

Priorities of patients and professionals over time (objectives i and ii)

A total of ten combined themes were identified as relating to participants' priorities during burn recovery. Six themes were most important for patients and professionals ('pain and discomfort', 'psychological wellbeing', 'healing', 'scarring', 'function', 'infection') and four themes were identified as most important to only patients or professionals ('uncertainty', 'patient knowledge, understanding & support', 'sense of control', 'survival'). There were differences in the relative importance of these priorities over time, and differences between patients and professionals. An overview of combined themes and their importance for patients and professionals at different time points during recovery can be found in Figure 1. A detailed account of all themes identified for each stakeholder group and how each is linked to a combined theme and outcome domains can be found in Tables S1-S2 of Supplemental file_Descriptive reports. The combined themes are summarised below and presented in order of their prevalence in the data, ascertained by the number of coded references.

<u>*Pain and discomfort (patients and professionals)*</u>

Pain was a concern common to patients and professionals in the short and medium term. Pain was a major cause of distress for the majority of patients and their family members as a result of the injury and initial treatment.

Professionals were particularly concerned about pain and adequate pain management immediately after the injury to prevent distress affecting patients in the long term. Reducing discomfort during

For peer review only - http://bmjopen.bmj.com/site/about/guidelines.xhtml

wound healing, such as easing itching and skin tightness were also raised as important in the medium term.

Some patients were worried about pain throughout their recovery. Especially, patients with larger burns, who complained about different aspects of long-term pain. For example, itching, scar tightness and contractures causing discomfort, neuropathic pain during nerve regeneration were all mentioned. Professionals, however, did not mention pain as a general concern in the long term.

> *"Managing the pain is the immediate thing." [professional, BCH30]*
>
> *"When it first happens it's just about the pain. That's it. […] that is just all you're focussing on at the time" [patient, NBT012]*
>
> *"So the biggest problem is sometimes, you know, a bit of pain in the leg for example, but it doesn't stop me doing things, you know, like walking but sometimes […] it is perhaps because it's tighter than it should be, I don't know, but it was more painful but its easing.' [patient NBT007]*

<u>*Psychological wellbeing (patients and professionals)*</u>

Psychological wellbeing and related impacts were concerns common for patients and professionals in the long term. Both groups' concerns were predominantly linked to scar appearance, mentioning internal (e.g. self-esteem, acceptance and normalisation of the injury) and external (e.g. bullying, worry related to intimate relationships and other people's views) influences on psychological wellbeing.

Professionals were mainly worried about long lasting impacts on patients' psychological wellbeing, manifesting in post-traumatic stress disorder (PTSD) from distress caused by injury and scarring. In particular, patients with larger burns described psychological impacts during the later phases of recovery (e.g. coping, PTSD).

For patients, the psychological effects of having a burn were apparent during all stages of recovery. In the short term, injuries affected individuals (e.g. worry and anxiety) and family members (e.g. anxiety and guilt, in particular parents). Psychological impacts were also highlighted in the medium term by patients (e.g. problems sleeping, flashbacks).

> *"I mean long-term the important outcomes really are how they are psychologically" [professional, BCH031].*
>
> *"When she saw her face she just broke down. She said 'I'm really ugly mummy'." [patient, BCH001]*

For peer review only - http://bmjopen.bmj.com/site/about/guidelines.xhtml

"I was just getting too upset and depressed over the fact that I had burns and I looked so different and had too many worries and thoughts in my head that I just really didn't want to think about" [patient, CW002]

Healing (patients and professionals)

Healing of the wound was a common concern, expressed frequently by professionals and patients. Importance of healing across the stages of injury recovery, however, differed across participant groups.

Professionals worried about healing in the short term. This was evident through narratives about concerns relating to adequate management of the wound during the acute injury phase, including a quick and smooth process of assessment, cleaning and dressing.

Patients' worry about healing was apparent in the medium to long term of recovery and often linked to feelings of surprise, frustration or worries about the amount of time it was taking for the wound to heal. The appearance of the wound was strongly linked to perceived length of healing times.

"Quite often we're very focused on dealing with the physical aspects of the burn injury, so making sure the burn wound heals, preventing infection. […] we're very focused on managing that wound and […] making sure the wound heals […]. [professional, BCH014]

"When you see it it's quite gnarly, […] like when its fresh you're like oh god, this is never going to heal" [patient, NBT002]

Scarring (patients and professionals)

Scarring was repeatedly mentioned by both participant groups to be of importance in the long term.

Professionals were mostly concerned about appropriate scar management to optimise their appearance and avoid restrictions on movement.

Similarly, patients' worries in the later phases of recovery spanned any need for further treatment and potential impacts on movement as well as the appearance of scars influencing self-esteem and body image that could affect psychological wellbeing. For patients, the worries about risk of visible scarring were also evident in the short and medium term of the recovery. Those concerns affected individuals and their family members in terms of worry about their appearance and associated future impacts on the patient (e.g. affecting psychological wellbeing).

"Long-term would be the scarring and whether contracture is affecting movement" [professional, BCH013]

For peer review only - http://bmjopen.bmj.com/site/about/guidelines.xhtml

"Well the worries were the scarring and whether we'd need to have- at that point in time we didn't know- no-one could tell us how bad it was." [patient, BCH001]

Just wondering what the scarring would do, whether the skin would be very tight. Because you do see people who've had burns, like facial burns and sometimes their skin is very pink and red and then you do notice it. But yes, I think that was about it really. It's just how functional your hands would be when they're recovered. [patient, NBT027]

Function (patients and professionals)

Return to normal functioning was a concern common to patients and professionals in the medium and long term. Participants discussed worries about impacts of the injury (or scar) on daily activities and how it affected patients returning to their normal lives was an important concern following discharge.

Professionals' priorities in relation to function were also focussed on aspects of mobility, primarily ensuring physical movement.

For patients, return to normal function was also a priority in the short term by highlighting concerns about the ability to return to work.

"The earlier you can intervene with physio then that's going to help with movement, […] it's important ongoing as well if the wounds are that bad that there is movement issues." [professional, BCH030]

"So initially I think it's kind of their return to their sort of family roles and their normal activities, when can I go back to work, when can I do my leisure activities." [professional, NBT004]

"I was concerned with my hands to start off with because that is my livelihood." [patient, NBT012]

"Particularly bathroom […], I absolutely hated the bedpans but I was too weak to get out of bed" [patient, CW006]

Infection (patients and professionals)

Infection was mentioned by patients and professionals. Importance of infection over time, however, differed across participant groups.

Preventing infection was one of the main priorities for professionals in the short and medium term. Following patients' hospital admission, professionals highlighted the importance of adequate cleaning and preparation of the wound to reduce healing time and scarring. Worry about cleanliness extended to the medium term of burn recovery when professionals wanted to ensure *"that the*

For peer review only - http://bmjopen.bmj.com/site/about/guidelines.xhtml

patient understands how to look after their wounds, and to look out for signs of infection" [professional, NBT026].

Patients discussed worries about infection related to the medium term of their recovery. Whilst most interviewed patients were aware of the need to keep the wound clean, infection was discussed as major concern primarily by those patients who had experienced symptoms of infection.

> *"Just to make sure there's no infection. If there's a lot of dead skin hanging around or blisters aren't de-roofed you could potentially get an infection just in the fluid that's still around or the dead skin." [professional, BCH001]*

> *"I thought that I had been released for good and was at home and actually I was extremely unwell. Night sweats, worrying about all sorts, just want, you know, really just completely in the dark about what was going to happen and I think because there's so much forewarning that it can happen, you know, then the worst does. It's a very upsetting thing to go through." [patient, CW004]*

<u>*Patient knowledge, understanding & support (professionals only)*</u>

This theme was identified as a unique priority for professionals. In the medium term, at the point of discharge, professionals felt it was crucial to ensure patients are *"well equipped with the information that they need" [professional, BCH006].* This encompassed information related to support the self-management of the wound (e.g. creaming, massaging, sun protection). Professionals' main objectives were also to help patients understand their injury (e.g. recognise signs of infection) and gain self-confidence in further wound treatment, so they remain as independent from the service as possible. Interviewees also repeatedly mentioned the importance of communicating the constant support available to patients which included readiness to answer any questions and psychological support.

> *"It's explaining to them what they need to be doing to achieve this wound to heal" [professional, NBT026]]*

> *"My biggest goal and concern for them is can they access everything they want to access." [professional, BCH007]*

<u>*Uncertainty (patients only)*</u>

Uncertainty was identified as a unique theme for patients and was an important concern during the earlier stages of recovery. Feelings of uncertainty related to how the injury happened, the treatments needed and their possible outcomes as well as the likely course of recovery. Participants associated feelings of anxiety with uncertainty but also described how information provision (e.g.

For peer review only - http://bmjopen.bmj.com/site/about/guidelines.xhtml

For peer review only

about recovery and further treatment) can lessen those feelings. For patients with more severe burns, uncertainties remained in the long term and were mostly related to scar management and future treatment plans.

> *"During the time that we were kind of up in the air as to whether he would have the operation or not, we saw loads of different people on different rotas and stuff, and I kind of felt like there were lots of different opinions flying around about how well it was healing, how well it wasn't healing, what the next course of treatment would be. And although I understand why that was the case, because in the main they were kind of waiting to see what the wound was going to do, we felt very up in the air about […] what was going to happen, and whether what happened eventually would have actually been the right thing." [patient, BCH017]*

> *"Yeah, you've got no information, no knowledge and the pain materialises, you don't know whether that's supposed to happen, was that not supposed to happen, is something going wrong." [patient, NBT012]*

Sense of control (professionals only)

For a smaller number of professionals, the sense of feeling in control of the treatment process was identified as a priority in the short term. This included major concerns about being in control of the treatment situation (e.g. managing distress well) and technical steps involved. Professionals described the importance of feeling well prepared for the patients' treatment and the presence of a well-functioning team to support a smooth treatment. Consequence of lack of control were mentioned and included impacts on self-confidence and patients' confidence in the care.

> *"It's being in control of that procedure. I feel as a clinical professional, […] once you've lost the control of the procedure in some way then you've lost the parents' confidence in you." [professional, BCH006]*

Survival (professionals only)

Some professionals mentioned survival to be the key priority in the short term and was only discussed in the context of larger burns. For those professionals involved in their care, mortality-related outcomes and physiological signs (e.g. organ failure, fever) were a major concern. Only a minority of patients experienced life-threatening injuries, but those with major burns generally did not discuss survival. This may be caused by *"a kind of hole in their memory and they don't remember what happened to them" [professional, MH006]*, aggravated by severe pain relief and reduced levels of consciousness.

For peer review only - http://bmjopen.bmj.com/site/about/guidelines.xhtml

"Well you'd be looking at their physical signs and outcomes, things like their vital signs, their bloods, whether they're being ventilated and they're on inotropes, and also whether they're having runs of sepsis and whether they've gone into acute renal failure" [professional, NBT003]

"The most important outcome is survival because there are those patients that are very poorly and you just want them to survive". [professional, BCH007]

Mapping themes to outcomes (objective iii)

A total of seven out of 10 themes (70%) were identified as recognised outcomes in burn research. These were evident in a recently published systematic review that investigated all outcomes reported in burn care research [14] and identified research studies (see Table 2). An overview of identified exemplary studies assessing an outcome for which conceptual content aligns with themes identified in this study is shown below.

For peer review only

Table 2 Comparison of identified themes and outcomes measured in trials

Themes identified	Outcome domains mapped for both patients and professionals	Outcome domains mapped for patients only	Outcome domains mapped for professionals only
**Pain and discomfort**	• Effect of scar on movement (contractures)<br>• Burn wound pain	• Pain during procedures<br>• Itch<br>• Scar pain<br>• Comfort of dressings	• Use of medicines to treat symptoms
**Psychological wellbeing**	• Quality and quantity of sleep<br>• Psychological well-being<br>• Generalised anxiety<br>• Mental ability		
**Function**	• Ability to carry out daily tasks<br>• Return to work/school or previous function	• Appearance	• Mobility
**Healing**	• Burn wound healing	• Appearance	
**Infection**	• Burn wound infection	• Sepsis	
**Survival**		• Death from burn injury<br>• Death from any cause	
**Uncertainty**	None identified		
**Patient knowledge, understanding & support**	None identified		
**Sense of control**	None identified		

For peer review only - http://bmjopen.bmj.com/site/about/guidelines.xhtml

There were three themes (30%) for which no outcome domains could be mapped. In particular, 'uncertainty', 'patient knowledge, understanding & support' and 'sense of control' were all themes uniquely identified in this study [14].

DISCUSSION

This study used semi-structured interviews to investigate perspectives of 53 burn patients, and professionals involved in their care, to explore what is most important as the patients recover. The study identified a total of ten themes across both participant groups that related to priorities during recovery from a burn injury. Of those, six were identified across both participant groups ('pain and discomfort, 'psychological wellbeing', 'healing', 'scarring', 'function' and 'infection'), three were unique to professionals ('patient knowledge, understanding & support', 'sense of control', 'survival') and one was unique to patients ('uncertainty'). The study results also showed that the importance of recovery priorities changes over time (e.g. 'survival' was a concern in the short term but not in the medium to long term), and that priorities differed between patients and professionals (e.g. 'pain and discomfort' was a concern in the short to medium term for professionals but important to many patients throughout their recovery). Finally, this work established that seven identified themes overlapped with outcomes commonly reported in burn research, whereas three themes could not be mapped to reported outcomes ('uncertainty', 'patient knowledge, understanding & support', 'sense of control').

This work identified unique differences between what patients and professionals view as most important during burn recovery and supports the time sensitivity of priorities of patients and professionals. We were unable to find existing empirical evidence from burn care research that shows similar findings. This work, however, does complement other recent efforts to improve patient-centred outcome measurement in burn care research [3,6,7,12] by highlighting the need to align outcome assessment with the changing importance of priorities for different participant groups, and with the change in relevance of priorities at different times after injury or intervention. Research in other healthcare areas has started to acknowledge these complexities of outcome assessment during recovery. Development of a core outcome set for childhood asthma, for example, reported the differences in perspectives of patients and professionals as to which outcomes are most important [32]. Other core outcome sets for lower limb amputation or induction of labour, for example, included separate outcomes relevant to short and medium term recovery [33–35]. This

For peer review only - http://bmjopen.bmj.com/site/about/guidelines.xhtml

study supports a recommendation for core outcome sets in burns care to differentiate between different time points [3], but current core outcome sets do not yet incorporate these complexities [16].

An aim of on-going research is to ensure that frequently measured outcomes adequately capture all important priorities related to burns recovery [6]. The results from mapping themes to commonly measured outcomes in burn care research highlighted potentially important priorities currently not considered. . Specifically, perceptions related to 'uncertainty' (patients), 'sense of control' and 'patient knowledge, understanding & support' (professionals) were all identified as important priorities at varying time points during recovery. The existence and impacts of uncertainty on patients are well known [36] and, as supported by our findings, and have also been linked to the level of information provision [37,38]. Conversely, professionals in our study highlighted that 'patient knowledge, understanding & support' is one of their key priorities during the medium term of patients' recovery from a burn injury. Routine assessment of perceptions relevant to level of uncertainty and information provision as well as assessing patients' level of knowledge and understanding and support received can be important beyond commonly measured outcomes. In addition, these measures of subjective perception might provide useful care quality indicators to highlight where improvements, e.g. in information provision, might be required. Measurement instruments assessing patients' sense of control in healthcare interventions exist [39,40]. Very few studies, however, report the need to feel in control from the perspectives of healthcare professionals [41], and we were unable to find a measure assessing professional specific sense of control. In our study, only a small number of professionals mentioned this as a priority which warrants further research investigating the importance and impacts of perceived sense of control.

Strengths and limitations

Our findings integrated views from a large sample of patients and healthcare professionals (n=53) from various backgrounds, allowing investigation of a variety of perspectives. This means that the findings are broadly representative of commonly occurring burn injuries but simultaneously may not account for unique concerns of specific patient groups. For instance, where burn injuries were related to mental health issues (e.g. self-inflicted injuries), patients may display different patterns of priorities. Similarly, analysis focused on the most important and most commonly raised concerns which implies that individual experiences and priorities may differ to themes identified in this study. Other limitations should be acknowledged. First, our sample consisted of patients with a variety of burn size which has implications for elicited perceptions across different time frames investigated.

For peer review only - http://bmjopen.bmj.com/site/about/guidelines.xhtml

For instance, patients with larger burns experience longer healing [42].This study defined time frames (short, medium and long term) *a priori* which were agreed after expert consultation and discussions within the multi-disciplinary study team. Individuals' definitions of short, medium and long-term recovery, however, are highly personal and might depend on contextual complexities (e.g. size and location of burn, time since injury, socio-demographic details of patients, any wound complications), and might therefore differ to the definitions used in this study. Challenges to standardise time frames for recovery from burn injury are recognised considering the variabilities and complexities of injuries [42–44]. Long-term outcome measurement, for example, can range from a few months [45] to years [46] across studies assessing functional outcomes for large burns. Further research is warranted to pursue consensus definitions of time frames in burn recovery. In addition, time periods between participants' time of injury and interview varied across the sample, but no systematic variation in length of experience of recovery was pursued during recruitment. Due to resource limitations and practical considerations, the current work also did not employ a longitudinal study design but merely provides a snapshot of participants' perceptions. Quantitative and qualitative evidence suggests that varying recall periods may influence data collection in health research [47,48]. We were unable to collect reliable data on injury date to be able to assess the impact of potential recall bias caused by varying intervals on the results.

Second, our recruitment strategy meant that some patient groups may be underrepresented (e.g. patients with larger burns, adolescent patients). A small number of qualitative studies showed that different population groups experience recovery differently. For example, women were better able to adapt to living with a burn injury [49] and older adults require more support and information [50] following severe burn injury. Future research could explore priorities in more homogeneous patient groups with larger sample size, to investigate whether these change between different populations and type of injury. Likewise, professional participants were mainly female and had nursing roles and there were challenges in recruiting surgeons, mainly due to individuals' time constraints. It is unclear if, for example, perspectives of male and female professionals differ and whether a more gender balanced sample would have impacted the results. There is value in exploring this difference in future work. It is also acknowledged that our sample lacks ethnic diversity. Again, it would be of value to explore perspectives over time specific to certain ethnic minority groups to reveal potentially unique and important culturally dependent priorities [51,52].

Continued consensus work to refine existing core outcome and core measurement sets is crucial. Modular components of core sets using findings from this study could be developed, whereby different sets of outcomes are considered "core" to address priorities specific to time points and specific to different stakeholder groups. The use of applicable modules can accommodate

For peer review only - http://bmjopen.bmj.com/site/about/guidelines.xhtml

differences in stakeholder priorities and optimise outcome measurement relevant to short-, medium- and long-term priorities. This would reduce research waste and outcome measurement burden, but requires effective strategies for implementation in practice [53]. Further research is also needed to better understand impacts of time sensitivity of outcomes during the recovery from burn injuries. This work provided a starting point to understanding the variability of the most important and broad priorities of patients and professionals during burn recovery but does not provide an outcome categorisation. Additional qualitative work might assess more granular aspects of themes identified in this work amongst more homogenous patient groups by drawing on existing outcome taxonomies [6,7,14,54]. This can include, for instance, exploring more nuanced psychological concepts that are important to specific patient groups (e.g. adolescents or ethnic minority patients) or specific injury patterns (e.g. large burns) over time. Likewise, priorities important across all stages of recovery require further detailed examination to be able to inform specific outcome measurement. Indeed, this work does not attempt to recommend how outcomes should be measured and further in-depth work is required for this. For example, different concepts related to pain (scar pain, burn wound pain, pain during the procedure) might be relevant at different time points. Further empirical work is necessary to explore commonly understood time frames for recovery periods (e.g. short, medium, long term) which may facilitate the standardisation of time frames for outcome measurement in trials. This would allow efficient integration of essential and meaningful outcome measures into trial design, avoiding research waste and enabling tailored treatment and intervention development in burns care.

CONCLUSION

The study recommends that priorities (important outcomes) in burns care are measured at different time points during the recovery from a burn injury to accurately reflect complexity of burn care. In addition, it is suggested that different outcomes are considered for both patients and professionals and the inclusion of priorities not currently considered has the potential to uncover important areas for improvement of outcomes assessment. This may ultimately benefit burn victims by more effective and improved patient-centred care. Further work is needed to elucidate nuances of outcomes for specific patient groups and/or specific burn presentations and harmonise time periods within outcome measurement for efficient integration of time sensitivity of outcomes.

For peer review only - http://bmjopen.bmj.com/site/about/guidelines.xhtml

REFERENCES

1 NHS Digital. HES Data Dictionary: Admitted Patient Care. 2019.

2 NHS England. NHS STANDARD CONTRACT FOR SPECIALISED BURNS CARE (ALL AGES). 2013. https://www.england.nhs.uk/wp-content/uploads/2014/04/d06-spec-burn-care-0414.pdf (accessed 5 Jun 2021).

3 Falder S, Browne A, Edgar D, *et al.* Core outcomes for adult burn survivors: A clinical overview. Burns. 2009;**35**:618–41. doi:10.1016/j.burns.2008.09.002

4 Brown T, Mills S, Muller M. If it can't be measured it can't be managed: The paucity of outcome measures in burn care. Burns. 2003;**29**:757. doi:10.1016/j.burns.2003.09.006

5 Lemon J, Cooper J, Defres S, *et al.* Understanding parental perspectives on outcomes following paediatric encephalitis: A qualitative study. *PLoS One* 2019;**14**:1–15. doi:10.1371/journal.pone.0220042

6 Mathers J, Moiemen N, Bamford A, *et al.* Ensuring that the outcome domains proposed for use in burns research are relevant to adult burn patients: A systematic review of qualitative research evidence. *Burn Trauma* 2021;**8**:30. doi:10.1093/BURNST/TKAA030

7 Jones LL, Calvert M, Moiemen N, *et al.* Outcomes important to burns patients during scar management and how they compare to the concepts captured in burn-specific patient reported outcome measures. *Burns* 2017;**43**:1682–92. doi:10.1016/j.burns.2017.09.004

8 Mc Kittrick A, Gustafsson L, Hodson T, *et al.* Exploration of individuals perspectives of recovery following severe hand burn injuries. *Burns* Published Online First: 29 April 2022. doi:10.1016/J.BURNS.2022.04.026

9 Robert R, Meyer W, Bishop S, *et al.* Disfiguring burn scars and adolescent self-esteem. *Burns* 1999;**25**:581–5. doi:10.1016/S0305-4179(99)00065-0

10 Mathers J. Towards the Holistic Assessment of Scar Management Interventions. *Eur Burn J 2022, Vol 3, Pages 207-210* 2022;**3**:207–10. doi:10.3390/EBJ3010018

11 Kornhaber RA, De Jong AEE, McLean L. Rigorous, robust and systematic: Qualitative research and its contribution to burn care. An integrative review. *Burns* 2015;**41**:1619–26. doi:10.1016/J.BURNS.2015.04.007

12 Simons M, Price N, Kimble R, *et al.* Patient experiences of burn scars in adults and children and development of a health-related quality of life conceptual model: A qualitative study. In: *Burns*. Elsevier Ltd 2016. 620–32. doi:10.1016/j.burns.2015.11.012

13 Kool MB, Geenen R, Egberts MR, *et al.* Patients' perspectives on quality of life after burn. *Burns* 2017;**43**:747–56. doi:10.1016/j.burns.2016.11.016

14 Young AE, Davies A, Bland S, *et al.* Systematic review of clinical outcome reporting in randomised controlled trials of burn care. *BMJ Open* 2019;**9**:e025135. doi:10.1136/BMJOPEN-2018-025135

15 Simons M, Lim PCC, Kimble RM, *et al.* Towards a clinical and empirical definition of burn scarring: A template analysis using qualitative data. *Burns* 2018;**44**:1811–9. doi:10.1016/J.BURNS.2018.04.006

16 Young A, Brookes S, Rumsey N, *et al.* Agreement on what to measure in randomised controlled trials in burn care: Study protocol for the development of a core outcome set. *BMJ*

For peer review only - http://bmjopen.bmj.com/site/about/guidelines.xhtml

Open 2017;**7**:e017267. doi:10.1136/bmjopen-2017-017267

17 Williamson PR, Altman DG, Blazeby JM, *et al.* Developing core outcome sets for clinical trials: Issues to consider. *Trials* 2012;**13**:1–8. doi:10.1186/1745-6215-13-132

18 O'Brien BC, Harris IB, Beckman TJ, *et al.* Standards for reporting qualitative research: A synthesis of recommendations. *Acad Med* 2014;**89**:1245–51. doi:10.1097/ACM.0000000000000388

19 Bunniss S, Kelly DR. Research paradigms in medical education research. *Med Educ* 2010;**44**:358–66. doi:10.1111/J.1365-2923.2009.03611.X

20 Patton MQ. *Qualitative research & evaluation methods : integrating theory and practice*. SAGE Publications 2002.

21 Van Baar ME, Essink-Bot ML, Oen IMMH, *et al.* Functional outcome after burns: A review. *Burns* 2006;**32**:1–9. doi:10.1016/J.BURNS.2005.08.007

22 Braun V, Clarke V. Using thematic analysis in psychology. *Qual Res Psychol* 2006;**3**:77–101. doi:10.1191/1478088706qp063oa

23 Joffe H, Yardley L. Content and thematic analysis. In: Marks D, Yardley L, eds. *Research methods for clinical and health psychology*. London: : Sage 2004. 56–69.

24 Fusch PI, Ness LR. Are We There Yet? Data Saturation in Qualitative Research. *Qual Rep* 2015;**20**:1408–16.http://www.nova.edu/ssss/QR/QR20/9/fusch1.pdf (accessed 12 May 2022).

25 Hennink MM, Kaiser BN, Marconi VC. Code Saturation Versus Meaning Saturation: How Many Interviews Are Enough? *Qual Health Res* 2017;**27**:591–608. doi:10.1177/1049732316665344

26 Saunders B, Sim J, Kingstone T, *et al.* Saturation in qualitative research: exploring its conceptualization and operationalization. *Qual Quant* 2018;**52**:1893. doi:10.1007/S11135-017-0574-8

27 Fofana F, Bazeley P, Regnault A. Applying a mixed methods design to test saturation for qualitative data in health outcomes research. *PLoS One* 2020;**15**. doi:10.1371/JOURNAL.PONE.0234898

28 Nelson J. Using conceptual depth criteria: addressing the challenge of reaching saturation in qualitative research: *https://doi.org/101177/1468794116679873* 2016;**17**:554–70. doi:10.1177/1468794116679873

29 Morse JM. Data were saturated... *Qual Health Res* 2015;**25**:587–8. doi:10.1177/1049732315576699

30 Morse WC, Lowery DR, Steury T. Exploring Saturation of Themes and Spatial Locations in Qualitative Public Participation Geographic Information Systems Research. *http://dx.doi.org/101080/089419202014888791* 2014;**27**:557–71. doi:10.1080/08941920.2014.888791

31 Edlich RF, Larkham N, O'Hanlan JT, *et al.* Modification of the american burn association injury severity grading system. *J Am Coll Emerg Physicians* 1978;**7**:226–8. doi:10.1016/S0361-1124(78)80381-5

32 Sinha IP, Gallagher R, Williamson PR, *et al.* Development of a core outcome set for clinical trials in childhood asthma: a survey of clinicians, parents, and young people. *Trials 2012 131* 2012;**13**:1–9. doi:10.1186/1745-6215-13-103

For peer review only - http://bmjopen.bmj.com/site/about/guidelines.xhtml

33 Ambler GK, Brookes-Howell L, Jones JAR, *et al.* Development of Core Outcome Sets for People Undergoing Major Lower Limb Amputation for Complications of Peripheral Vascular Disease. *Eur J Vasc Endovasc Surg* 2020;**60**:730–8. doi:10.1016/J.EJVS.2020.06.021

34 Ambler GK, Bosanquet DC, Brookes-Howell L, *et al.* Development of a core outcome set for studies involving patients undergoing major lower limb amputation for peripheral arterial disease: study protocol for a systematic review and identification of a core outcome set using a Delphi survey. *Trials 2017 181* 2017;**18**:1–7. doi:10.1186/S13063-017-2358-9

35 Dos Santos F, Drymiotou S, Antequera Martin A, *et al.* Development of a core outcome set for trials on induction of labour: an international multistakeholder Delphi study. *BJOG An Int J Obstet Gynaecol* 2018;**125**:1673–80. doi:10.1111/1471-0528.15397

36 Auerbach SM, Martelli MF, Mercuri LG. Anxiety, information, interpersonal impacts, and adjustment to a stressful health care situation. *J Pers Soc Psychol* 1983;**44**:1284–96. doi:10.1037/0022-3514.44.6.1284

37 Andrew JM. Recovery from surgery, with and without preparatory instruction, for three coping styles. *J Pers Soc Psychol* 1970;**15**:223–6. doi:10.1037/H0029442

38 Gammon J, Mulholland CW. Effect of preparatory information prior to elective total hip replacement on post-operative physical coping outcomes. *Int J Nurs Stud* 1996;**33**:589–604. doi:10.1016/S0020-7489(96)00019-3

39 Tomsis Y, Perez E, sharabi L, *et al.* Postpartum Post-Traumatic Stress Symptoms Following Cesarean Section—the Mediating Effect of Sense of Control. *Psychiatr Q 2021 924* 2021;**92**:1839–53. doi:10.1007/S11126-021-09949-0

40 Lau-Walker MO, Cowie MR, Roughton M. Coronary heart disease patients' perception of their symptoms and sense of control are associated with their quality of life three years following hospital discharge. *J Clin Nurs* 2009;**18**:63–71. doi:10.1111/J.1365-2702.2008.02386.X

41 Southwick FS, Southwick SM. The Loss of a Sense of Control as a Major Contributor to Physician Burnout: A Neuropsychiatric Pathway to Prevention and Recovery. *JAMA Psychiatry* 2018;**75**:665–6. doi:10.1001/JAMAPSYCHIATRY.2018.0566

42 Pham TN, Kramer CB, Wang J, *et al.* Epidemiology and outcomes of older adults with burn Injury: An analysis of the national burn repository. *J Burn Care Res* 2009;**30**:30–6. doi:10.1097/BCR.0b013e3181921efc

43 Ryan CM, Lee A, Kazis LE, *et al.* Recovery trajectories after burn injury in young adults: Does burn size matter? *J Burn Care Res* 2015;**36**:118–29. doi:10.1097/BCR.0000000000000214

44 Mason ST, Esselman P, Fraser R, *et al.* Return to work after burn injury: A systematic review. J. Burn Care Res. 2012;**33**:101–9. doi:10.1097/BCR.0b013e3182374439

45 Cucuzzo NA, Ferrando A, Herndon DN. The Effects of Exercise Programming vs Traditional Outpatient Therapy in the Rehabilitation of Severely Burned Children. *J Burn Care Rehabil* 2001;**22**:214–20. doi:10.1097/00004630-200105000-00006

46 Druery M, Brown TLH, Muller M. Long term functional outcomes and quality of life following severe burn injury. *Burns* 2005;**31**:692–5. doi:10.1016/J.BURNS.2005.03.001

47 Stull DE, Leidy NK, Parasuraman B, *et al.* Optimal recall periods for patient-reported outcomes: challenges and potential solutions. *https://doi.org/101185/03007990902774765* 2009;**25**:929–42. doi:10.1185/03007990902774765

48 Dolan AR, Goldberg EB, Cannuscio CC, *et al.* Patient Perceptions About Opioid Risk

For peer review only - http://bmjopen.bmj.com/site/about/guidelines.xhtml

Communications Within the Context of a Randomized Clinical Trial. *JAMA Netw Open* 2022;**5**:e2227650–e2227650. doi:10.1001/JAMANETWORKOPEN.2022.27650

49 Williams NR, Davey M, Klock-Powell K. Rising from the Ashes. *http://dx.doi.org/101300/J010v36n04_04* 2008;**36**:53–77. doi:10.1300/J010V36N04_04

50 Jones BA, Buchanan H, Harcourt D. The experiences of older adults living with an appearance altering burn injury: An exploratory qualitative study. *J Health Psychol* 2017;**22**:364–74. doi:10.1177/1359105315603473

51 Rawlins JM, Khan AA, Shenton AF, *et al.* Burn patterns of Asian ethnic minorities living in West Yorkshire, UK. *Burns* 2006;**32**:97–103. doi:10.1016/j.burns.2005.08.013

52 Tan KT, Prowse PM, Falder S. Ethnic differences in burn mechanism and severity in a UK paediatric population. *Burns* 2012;**38**:551–5. doi:10.1016/j.burns.2011.10.005

53 Hughes KL, Clarke M, Williamson PR. A systematic review finds Core Outcome Set uptake varies widely across different areas of health. *J Clin Epidemiol* 2021;**129**:114–23. doi:10.1016/J.JCLINEPI.2020.09.029

54 Dodd S, Clarke M, Becker L, *et al.* A taxonomy has been developed for outcomes in medical research to help improve knowledge discovery. *J Clin Epidemiol* 2018;**96**:84–92. doi:10.1016/j.jclinepi.2017.12.020

For peer review only

For peer review only - http://bmjopen.bmj.com/site/about/guidelines.xhtml

Acknowledgements

We would like to thank the research nurses Karen Coy, Catherine Spry and Anthony Sack for their help with recruitment for this study. We also thank all participants for dedicating their time and views to this study.

Professor Amber Young, senior author, colleague, and friend sadly passed away before the publication of this manuscript. Amber dedicated her career to advancing burn care research and worked tirelessly to improve outcomes for burn survivors. It was our privilege to work with Amber and to benefit from her skills, passion, and knowledge. She will be truly missed.

Contributors

AEY conceived the project. CH wrote the manuscript with the support of AEY, PD and DE. CH and AEY performed data collection. CH and PD performed data analysis with support of AYE and DE. DE is a qualitative researcher and provided oversight and guidance on methods. AEY and DE edited and critically revised draft manuscripts. AYE provided strategic oversight over the project. All authors have read and approved the publication.

Funding

This research is funded by The Scar Free Foundation, UK. The Scar Free Foundation is the only medical research charity focused on scarring with the mission to achieve scar free healing within a generation. The project was also supported by the National Institute for Health and Care Research (NIHR) Biomedical Research Centre (BRC) at the University Hospitals Bristol and Weston NHS Foundation Trust and the University of Bristol (BRC-1215-20011). AY has received an NIHR Doctoral Research Fellowship (DRF-2016-09-031 NIHR). This paper represents independent research funded by The Scar Free Foundation. The views expressed in this publication are those of the authors and do not necessarily reflect those of the Scar Free Foundation, the UK NHS or the NIHR.

Competing interests

All authors declare no competing interests.

For peer review only

Patient consent for publication

As part of the consent process, patient participants agreed to their anonymised quotes being published in scientific journals.

Ethics approval

A favourable opinion for the project was granted by the South West - Frenchay Research Ethics Committee, reference 17/SW/0025. AY and CH obtained the written informed consent of each participant before conducting the interviews. It was also ensured participants had read the study information sheet sent to them prior to the interview and that they were informed about their rights as participants, that the conversation would remain confidential, and that the transcript would be anonymised.

Provenance and peer review

Not commissioned; externally peer reviewed

Data availability statement

All relevant data is included in the main body or the supplementary files. The raw datasets used and analysed during the current study are available from the corresponding author upon reasonable request.

For peer review only

For peer review only - http://bmjopen.bmj.com/site/about/guidelines.xhtml

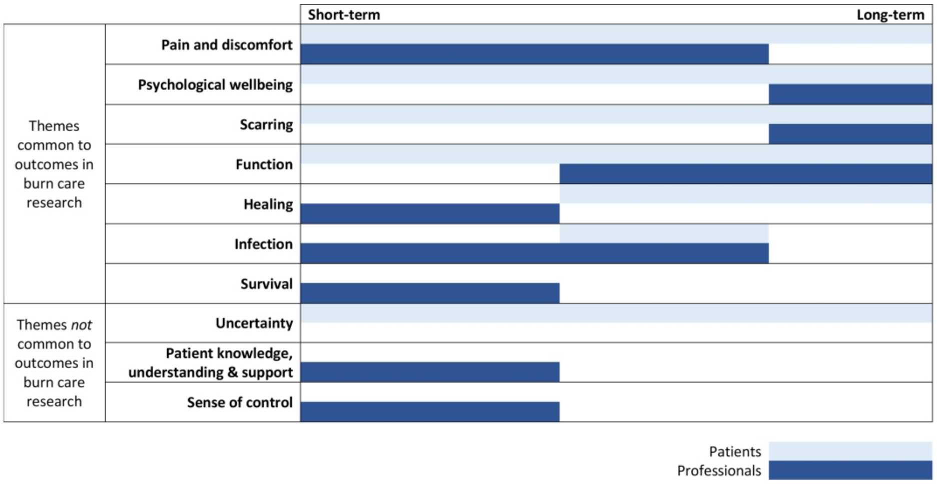

Figure 1: Overview of patients' and professionals' priorities over time

246x129mm (600 x 600 DPI)

Supplemental File - Table S1: Exemplary interview topic guide for patient participants

Topic	Questions
**Intro**	• Introduction to researcher, topic, aims and objectives of the study.<br>• Ask if any questions<br>⇒ *Check that participant is happy to audio record the interview and check consent*
**Background/ warm-up**	• First of all, could you tell me a bit about you?<br>○ *Prompt: do you work, do you have children etc*<br>○ *Prompt for children: how old are you?*<br>• Could you tell me about how you/your child came to have the burn?
**Short-term: Immediately after injury**	• Thinking about the time when you were burned, what were your worries about the immediate effects of the burn at that time?<br>○ *Prompt: e.g. survival, pain*<br>○ *Anything else you can think of? Any other concerns you may have had?*<br>• Still thinking about the time when you were burned, what were your concerns about future effects?<br>○ *Prompt: scarring, movement*<br>*Areas of your life that it was most important to ensure the burn didn't affect eg function (eg related to work, school), the look of the burn (confidence, work, school, friends)? Anything else?*<br>• **What would you say were the most important aspects during that time?**
**Medium-term: After discharge**	• Thinking about the time when you started to recover/got home/were discharged, what were your concerns in relation to your recovery?<br>○ *Was your daily life affected? If so, in what ways? Return to normal.*<br>▪ *Prompts could include work life; social life; family life; relationships (including intimacy)*<br>○ *Were there any issues that affected you that the doctors didn't ask about at your out-patient appointments?*<br>○ *Were there issues that bothered you more than they seemed to bother the medical staff?*<br>○ *Were there issues that the doctors asked about that didn't worry you?*<br>• During the recovery (at home), what were your concerns specifically about the future?<br>○ Prompt: QoL, physical,<br>• Were your family and friends (and if applicable, partner) worried about any effects of your burn at any time after the burn injury? If so, what were they worried about?<br>○ *Prompt: (as above; work life; social life; family life; relationships (including intimate relationships)*<br>○ *Anything else? What other concerns were there?*<br>• **What, in your view, was most important to you during the time after discharge?**
**(Current) Recovery & Future**	• What matters to you (now) while you are getting better?<br>○ *Prompts: similar to above and laddering questions*<br>• And thinking about the future, what are you particularly worried about?<br>• **And again, thinking about your situation now, what is most important to you at the moment in relation to your recovery?**<br>○ Do you think this will matter further down the line?<br>○ If there are any areas of healthcare that could have been improved, what would that/they be?<br>○ *Prompt: treatment of infection, management of pain, improving success of skin-grafting?*
**Wrap-up questions**	• To finish with, could we summarise what the most important areas of your recovery after the injury were to you?<br>⇒ *Wrap up interview*

For peer review only

Supplemental File - Table S2: Exemplary interview topic guide for professional participants

Topic	Questions
**Intro**	• Introduction to researcher, topic, aims and objectives of the study.<br>• Ask if any questions<br>⇒ *Check that participant is happy to audio record the interview and check consent*
**Background/ warm-up**	• First of all, could you tell me a bit about you and your role as a healthcare professional?<br>○ *Prompt: how long have you been at the hospital for, what are your responsibilities?*<br>○ *What is your involvement with burns injuries? How long working in burns?*<br>• What are your experiences been with burns patients?<br>• *Which part of the recovery journey are you involved in? When/how regular do you see them?*
**Short-term: Immediately after injury**	• Going back to the very beginning, when patients are admitted to the hospital, what are the relevant outcomes from a clinical perspective?<br>○ E.g. survival, scaring, pain?<br>• **What is the most important outcome?**<br>• What do you think patients are worried about the most during that time?<br>• **What do you think is the most important outcome for patients at that time?**
**Medium-term: After discharge**	• Moving on to when the patient gets discharged for recovery at home, what are relevant outcomes for you from a clinical viewpoint?<br>• **And what was the most important one taking into consideration the recent discharge?**<br>• And again, what do you think the patients are concerned about when they get discharged from hospital?<br>○ *Prompt: daily life, QoL, work, social,*<br>• **What do you think is most important to the patients at the time they are being discharged?**<br>• How do you think do the patients views differ from their families and friends? What are they worries about?<br>• *Prompt: (as above; work life; social life; family life; relationships (including intimate relationships)*
**(Current) Recovery & Future**	• Thinking about burns injury outcomes further down the line, what is important for you in the long run? What is most important?<br>○ What do you think is the most important <u>to patients</u> when thinking about the really long-term effects in the future?
**Wrap-up questions**	• To finish with, could we summarise what the most important areas of patient recovery after the injury?<br>⇒ *Thanks to interviewee*

For peer review only - http://bmjopen.bmj.com/site/about/guidelines.xhtml

Supplemental File – Table S3: Outcome classification for burn care research (adapted from Young et al. 2019)

Outcome category	Outcome domain
**Patient-reported**	Ability to carry out daily tasks
	Anxiety about medical procedures and appointments
	Generalised anxiety
	Appearance
	Blister fluid
	Burden of care
	Comfort of dressings
	Psychological well-being
	Mental ability
	Quality and quantity of sleep
	Effect of scar on movement (contractures)
	Return to work/school or previous function
	Burn wound pain
	blister fluid
	Pain during procedures
	Scar pain
	Itch
**Pathophysiological**	Ability to fight infection
	Body weight maintenance
	Bone strength
	Breathing and lungs
	Donor site problems after healing
	Effect of burn on genes
	Effect of burn on how the body uses energy
	Effect on heart and blood circulation
	Fitness
	Growth in children
	How well muscles work
	Mobility
	Kidney function
	Liver function
	Medical tests to indicate how unwell a patient is
	More than one organ failing (multiorgan failure)
	Muscle strength
	Stomach and bowel function
	Burn wound healing
	Donor site healing
**Complications**	Complications of drug treatment
	Blood product transfusion
	Burn wound infection
	Death from burn injury
	Death from any cause
	Effects of fluid from a drip
	Infections other than burn wound infection

For peer review only

	Sepsis
**Scar-related**	Scar colour
	Scar texture
	Scar size
	Treatment for scars
**Healthcare-related**	Costs of treatment for NHS/hospital
	Length of hospital stay
	Length of stay in intensive care unit
	Length of time on life support machine
	Use of medicines to treat symptoms

Young AE, Davies A, Bland S, Brookes S, Blazeby JM. Systematic review of clinical outcome reporting in randomised controlled trials of burn care. BMJ open. 2019 Feb 1;9(2):e025135.

For peer review only

Supplemental File – Descriptive reports

1. Interviews with patients

Scarring

Worries about future scarring were apparent for some participants and their families in the early days following the injury.

> *Well the worries were the scarring and whether we'd need to have- at that point in time we didn't know- no-one could tell us how bad it was. Before we got to (city) burns no-one could tell us how bad it was, so our worry was whether she needed to have a skin graft or was she going to have permanent scars because it was so horrible on her face-[BCH 01, parent]*

> *Just wondering what the scarring would do, whether the skin would be very tight. Because you do see people who've had burns, like facial burns and sometimes their skin is very pink and red and then you do notice it. But yes, I think that was about it really. It's just how functional your hands would be when they're recovered.[NBT 27]*

The majority of participants interviewed went on to develop some scarring as their wound healed. Many of these participants described the physical appearance of their scars, talking about the colour (*'really pink' [BCH FW] 'quite red, it's not a natural skin colour [NBT 005]*) and the shape (*'quite gnarly' [NBT 02], 'pretty bumpy' [BCH 19]*). One participant talked about how the appearance of their scar was getting worse during the healing process.

> *you look progressively worse as your function gets progressively better. So I had a- I struggled with the fact that I was physically feeling up to it, going out, doing things and I was looking more scarred, more bumpy because scar tissue takes a while to form and, you know, you just end up, as the peeling process happens, looking worse as time goes on until a point where then you'll stop and it will start to improve but you need to do a lot to improve the appearance so for the last three to four months I've been looking progressively worse.[C&W 004]*

Several participants talked about how their scars negatively affected their feelings about their appearance.

> *I felt I looked like Frankenstein [CW 002]*

> *How it looked was a big- I wouldn't be able to go out on a beach and just feel confident in a bikini or something [NBT 002]*

> *I worry this will be it, it's not going to get any better but I've said that every month since I was discharged and there's always been changes. But I cry… every time I go in I cry going is this it? I can't look like this for the rest of my life. [NBT 013]*

The reactions of others to their scars also had an impact. Several participants described being stared at by others. For some, the reactions of others were a strong determinant of how they themselves felt about their scars.

> *some guy on the bus, this was a grown man in his thirties, I was on the bus on my way to hospital once and I was standing 'cos there were no seats, he was looking at me and doing wretch noises as though he was vomiting, looking at me and doing-*

For peer review only

if other people were like accepting of it I would be more accepting of it, I wouldn't be as embarrassed but you get these people that are just really naïve. [C&W 002]

It's more a question of having an overall appearance that causes people to look at you in the street which is uncomfortable. If you had scars that were maybe subtle, not a big deal, some people might notice when they're close up but I think, you know, as the healing process happens with burns you go very red, very bumpy, it's really obvious and I think anything obvious in terms of the reaction you see from other people affects you more than how you see yourself. [C&W 004]

However, some participants said that they were not concerned about their scars.

Personally doesn't bother me, doesn't bother me at all. I can see why it might bother some people but personally no. [NBT 08]

Yeah, don't get me wrong, its not nice to have a scar like that but its not the end of the world, I'm not too vain that I can't still put shorts on sort of thing, but yeah, it is what it is. [NBT 12]

Older participants tended to be less affected by their scarring. The location of the scar was also important – participants with scarring in less visible places tended to be less bothered by it.

It could have been on my face which the ward that I was in at the time, there was a lady opposite me in the ward that had had full body burns from her Christmas lights going up and I looked at her and I thought ok, you need to be happy about this, it could have… its an impact but its… I'm left with a scar on my leg that is not the end of the world. [NBT 12]

So it's quite visible, it's from my chin, it's very thick on my neck, you can't miss it. I get funny looks from children and so yeah, and just… I feel like I'm being remembered for the girl with the burns and stuff like that. [NBT 13]

Pain

In the immediate aftermath of the burn, pain was an important concern, often the most important concern, for the majority of patients.

'Yeah. Yeah I don't think I would have thought of anything else. Yeah, just concerned about the pain.'

'At first its just, when it first happens its just about the pain. That's it. You can't really… because it wasn't… it was only that area, I was still completely compos mentis and I was in pain but really that is just all you're focussing on at the time, its just trying to mentally manage the pain and…'

Pain was also a concern for other family members

'It is just the realisation of sort of thinking [inaudible] when I realised how much pain he was in, how painful it was to clean it, how painful it was to dress it and you he is only tiny.' [parent]

However, some participants reported that pain was not an early concern.

'Yeah, my adrenalin was going so much that I didn't notice the pain actually.'

'There was no pain really. It's really strange, yeah. I think because I think the wound got quite deep with the acid burn it sort of killed away part of the nerves-'

Pain experienced by participants occurred not just as a result of the burn but also due to treatments received such as cleaning the wound, dressing changes, surgeries and skin grafts.

For peer review only

Adequate pain management was identified as important both in the short term and after discharge from hospital.

'Yeah, they kept me very well medicated. I don't remember being in terrible pain. I remember when they would ask me every day what my pain scale (inaudible) one to whatever and I'm sure I always answered quite low because they kept it so well'.

'they literally just on the Saturday morning came up and said consultant will be coming round, he might be able to sign you off. He didn't come round, the nurse came up and said ok you're good to go and just pushed me out the door with no medication, no occupational therapist, no guidance as to what expect from the wound, no knowledge as to what to expect with pain, no pain management tablets, no nothing, they literally just pushed me out the door.'

For some participants, adequate pain management needed to be balanced against its potential side effects

'But for me I preferred to cope with that for five minutes [of wound being washed down] than take a lot of painkillers which can give you other problems. '

For participants who experienced deeper burns, pain increased in the medium term due to nerve regeneration

'I thought that it doesn't bother me because I've got on a previous burn, um, I couldn't feel anything to start off with either but gradually I'm getting little shooting pains and I spoke to one of the doctors and he said it's because all the nerves are finally like reattaching and then it's a bit confused as to where to kind of attach to so that's where the little itchy kind of sharp pain is coming from so I'm hoping, 'cos this is only two months old-' [NBT009]

Although pain was less commonly reported as an issue in the long-term, it was clear that some participants continued to experience pain or sensitivity in the affected area and this was accepted as a part of life.

'because it's just sensitive, it's the sensitivity of it. Yeah, it's very sensitive on the little finger, my thumb and the palm of my hand. But I think as she said, give that another year and it will get better. But it's fine. ' [NBT027]

'Um, I don't know if its psychological but there's always a bit of pain, I don't know but you get used to it but I was worried at first could I do any movement or not, you know, because I didn't want to damage- 'cos they've done such good work as well.' [NBT007]

'So biggest problem is sometimes, you know, a bit of pain in the leg for example, but it doesn't stop me doing things, you know, like walking but sometimes like this morning I couldn't (inaudible) and its more painful than- it is perhaps because its tighter than it should be, I don't know, but it was more painful but its easing.' [NBT007]

Itching

Itching was a commonly reported problem amongst participants. For most, itching was not experienced immediately after the injury but commenced as the wound began to heal.

Oh itching was terrible! (laughs) And not being able to actually scratch it, so the only way I could do that was to move my foot around and the dressing would sort of work its way off (laughs) so, yeah I did get a lot of itching.[NBT 014]

sometimes I think the itch was worse than the pain [C&W 06]

Not initially. Probably after, ooh I reckon a month. Obviously when it started to heal I think, or that's what I assumed anyway. [NBT 014]

In addition to being unpleasant, some participants reported that itching interfered with daily living. Itching was described as being worse at night which affected sleep. Two participants described their anxiety about itching in public.

Yes. Yes. I mean it itches much less than it was but when I get hot, particularly at night that's when it's such- it's worse [NBT 011]

Itchiness. I feel like that was one of the most unbearable things ever and the constant anxiety that I'm going to start itching randomly in public and I can't control it [C&W 02]

Itching continued to be a problem for a long time for some participants, although most reported that it did ease in the long term.

would say, nearly five years in now, but the itch has gone. I can remember talking to another patient that I met that had a burn injury previous to mine and they said that it took quite a few years and it did, I think it took about four years to actually- [C&W 06]

Occasionally it itches. Very occasionally. Not very often though, no, but I still get that fizzy feeling sometimes, very rarely now but occasionally [NBT 014]

Tightness

Several participants talked about tightness in the area of their burns as they healed. For some participants this tightness was a source of discomfort and distress.

when I think back to it now my burns were actually rigid, they would not move. They were literally as if they were set in stone- [C&W 002]

I think there was- the skin was quite taut so therefore uncomfortable. [NBT011]

so once I started getting the movement back I was just scared, I literally thought that all my skin was going to just rip in half. It's quite a scary experience and I'm not- I don't think I'm alone on that, she said quite a lot of people feel that way. [NBT 013]

Tightness restricted participants ability to move and impacted on their activities. It was also a source of pain.

like if I go in the garden and do some weeding because it's (inaudible)- So to then down ways, I used to bend down ways with the legs straight but they say no you have to bend down like you would so it's trying to build down- now to protect your-[NBT 007]

So biggest problem is sometimes, you know, a bit of pain in the leg for example, but it doesn't stop me doing things, you know, like walking but sometimes like this morning I couldn't (inaudible) and its more painful than- it is perhaps because its tighter than it should be, I don't know, but it was more painful but its easing. [NBT 007]

Several participants described having treatments to help with the tightness which reduced over time. However, some participants reported that they continued to experience tightness in the longer term.

For peer review only

Infection

Several participants reported experiencing one or more infections in the period following their injury. Participants described the appearance of the infected area, smell and general feelings of being unwell. The appearance and smell were also a source of distress for some patients and family members.

> *'and then on the afternoon I looked and I could see all my wrist and hand getting quite puffy and red and swollen and I thought oh I don't like the look of that' [NBT 009]*

> *'cos the infection just made me feel really ill as well.' [NBT 002]*

> *'I'm sure dad won't mind me saying, when the infection was there the smell was horrendous,' [NBT 010]*

Infections sometimes led to a lengthened hospital stay or readmission to hospital. Participants were also concerned about the possibility that infection could spread from the burn site.

> *'But I think I was most worried about the infection, when they said that it was infected and obviously it could be in his bloodstream, I think that scared me the most.' [BCH 019]*

> *'Well when the nurse mentioned sepsis and things like that you start to panic.'[NBT014]*

Worries about developing an infection were a common theme for many participants in the early period following their injury, although there was some variability in this.

> *'Yeah, so pain it was ok and you could see it was slowly getting better and I was really panicked about infections. Infections really bothered me but once the sort of the weeping stopped it was ok, you could see it was healing so that went.' [BCH 018]*

> *'I worried an awful lot about that. I did worry an awful lot about infection.' [C&W 006]*

These worries were greatest in those who had experienced a prior infection.

> *'And then I think it was probably about a month later it looked- I wondered if it was going to be infected again so I went off to the A&E and they reassured me it wasn't.' [NBT014]*

Participants were also worried about keeping the injury clean in order to prevent infection

> *'I think it was more that he has trouble staying clean, you know, he's very hands on and, you know, likes playing out in the garden and when we went swimming and things like that, I think that was-' [BCH 03]*

Worries about developing an infection were not a concern in the long-term.

Appearance of the wound

Several participants talked about the appearance of their wound in the days following their injury. Participants described the blistering, the colour and the smell of their wound. Wound appearance was a source of distress for some patients and their significant others.

> *I was pretty horrified when I saw it. I don't know whether it was just 'cos it was just raw flesh. It was just raw. It was disgusting. [C&W 005]*

> *I think initially it was the look of it because that was pretty scary, that was pretty frightening.[BCH 020]*

The effect of the wound on their appearance was also a source of distress.

For peer review only

she didn't want it [the dressing] taken off because it hurt her and then when she saw her face she just broke down. She said I'm really ugly mummy.[BCH 001]

I was just getting too upset and depressed over the fact that I had burns and I looked so different [C&W 002]

The appearance of the wound informed participants views on the seriousness of their injury.

But when it comes to over 24 hours or whatever it started to look way worse, and I was much more worried about the seriousness of it. [BCH 017]

One participant remarked that they did not realise the seriousness when the wound was dressed preventing them from being able to see it.

I had no idea how bad it was. I was very bandaged up so no. [NBT 013]

For many participants, the appearance of the wound was linked to their feelings about the time it was taking to heal.

like when its fresh you're like oh god, this is never going to heal but yeah.[NBT 002]

I don't know because although its healed lovely, it's still loitering because I can still see- I mean where the skin's purple and sometimes I get like a white raised area in the middle and I think oh what's that? And when I (inaudible) the cream it's gone. It's almost like it's sort of hang on a minute, I'm taking my time, you know. [NBT 014]

Time to healing

Some participants expressed feelings of surprise, frustration or worries about the amount of time it was taking for the wound to heal. Many seemed to feel that the healing took longer than they had expected.

I don't worry about the scar side of it, it was just the... the worry side of it was actually just the time it was taking. [NBT 012]

I was just saying to (husband) the other day, he was like I can't believe how long it's all taking 'cos it happened, I don't know when it happened, like beginning of August and now we're in October and I feel like we're still kind of still doing it. [BCH F.W.]

At the time it took ages and it was a drag coming up here every other day. [NBT 019]

Uncertainty, lack of knowledge and the need for information

A lot of participants described feelings of uncertainty following their injury. These uncertainties related to the how the injury happened, the treatments needed and their possible outcomes as well as the likely course of their recovery.

And I suppose as well, because during the time that we were kind of up in the air as to whether he would have the operation or not, we saw loads of different people on different rotas and stuff, and I kind of felt like there were lots of different opinions flying around about how well it was healing, how well it wasn't healing, what the next course of treatment would be. And although I understand why that was the case, because in the main they were kind of waiting to see what the wound was going to do, we felt very up in the air about how... what was going to happen, and whether what happened eventually would have actually been the right thing. [BCH 0017]

For peer review only

Yeah, you've got no information, no knowledge and the pain materialises, you don't know whether that's supposed to happen, was that not supposed to happen, is something going wrong. [NBT 12]

A clear need for information to lessen feelings of uncertainty was apparent. Many participants spoke positively about staff and services who met their information needs and explained things in a non-technical manner. Some participants felt that they did not get enough information and might have coped better had they had done so. For participants with more severe burns requiring a hospital stay, adequate information at the point of discharge home was important as was the availability of a point of contact for any future questions.

Very good. (A) was particularly good because he spent so much time with me 'cos he was in and out and he explained everything so thoroughly in a way that you could understand yourself, if you know what I mean, rather than having to look words up. (laughs) It's when they talk in jargon, if you know what I mean.[NBT 014]

one bit of advice that I would give throughout this is to give the patient clarity as to what's happening and the expectations where I wasn't given enough information and knowledge of what's happening now, what's going to happen, what could happen, what might not happen, I was signed off from the hospital and they hadn't said to me that the nerve endings will come back and then you'll start to suffer pain. So when I was signed off I didn't have really very much pain within 36 hours, it nearly broke me where the- going from there down to stood up, the rush of blood to the burn, I didn't know to expect it so it was quite a shock to the system. So I would just say just information, information, information. [NBT 012]

Although increased knowledge was generally seen as a positive thing, some patients mentioned that there were downsides to having too much information.

me and my family are very inquisitive people that need, you know, to know every possible scenario so there were a couple of times when we thought I'd have to have operations on things that I didn't need to have an operation on in the end so I think on the one hand it's great to be told of the things that are potentially going to go wrong or are going wrong and on the other it gave us maybe unnecessary cause for concern about procedures I ended up not needing. [C&W 004]

Several patients acknowledged that some aspects of their treatment and recovery were simply uncertain and that it would never be possible to have complete information.

I'm just obviously concerned about the future and things that no-one can tell me like in two years' time will he still need to wear the sleeve or is he still going to have to be creaming but in regards to everything that I need to know now, I feel that they've been brilliant, they've explained everything to me and to (child). [BCH 019]

Although themes of uncertainty and the need for knowledge and information were most commonly described by patients during the earlier stages of their recovery, some participants who had been left with more serious scarring described long-term term uncertainties and informational needs, particularly in relation to scar management treatments.

the problem I'm having at the moment is that there's no (sighs) there's still so few guarantees, or there are none so it's always so difficult 'cos you don't want to be making something worse if what you care about is the aesthetic. [CW 001]

And then I think in terms of, this is a big ask really but I think in terms of kind of follow-up, if there are new things, you know, I took myself off to my GP and said can you re-refer me back into the system

For peer review only

'cos I want to see if there are new things but if there was ever an opportunity to be a forum or something to be kept abreast of new developments-[CW 001]

Psychological effects

Several participants reported feelings of shock or fear in the immediate aftermath of their injury. These feelings arose as a result of the circumstances in which the injury occurred or due to the extent of the injuries received.

The first part, on the day, it was just, yeah, the whole scorch thing, the instant impact of it all going off, the shock factor.[NBT 12]

Amongst participants whose injury required a hospital stay, several participants commented on the value of psychological support being available.

just to know someone was there actually so it didn't mean that I had to deal with it on my own. I didn't make full use of it, I didn't feel the need but it was there. [C&W 004]

In the weeks following the injury, some participants reporting difficulty sleeping or of having nightmares or flashbacks.

Yeah. I do remember having quite a lot of hallucinations and I now know they're not real but at the time, for the first couple of weeks when I was up on the burns ward I didn't sleep unless they knocked me out because I lived in fear that someone was going to come and get me and put me on fire again.[NBT 0013]

Some participants also experienced guilt as they blamed themselves for the injury.

I suppose because I was so upset, and because I felt completely responsible for what had happened, I was predicting that, and I was quite vocal about that to a few people. Not many people, you know, two or three people. So that in turn made them more worried about how much kind of guilt I was taking on and I [inaudible] responsible I felt for what happened. [BCH 017]

In some cases, participants' experiences of receiving treatments also had some psychological consequences. This was more common when the person who had the burn was a child. Parents described how their child was now fearful of situations that reminded them of unpleasant or painful treatments (e.g. bathing) or of their experiences of being in hospital.

I don't know if it's got anything to do with it but like he mentally changed when he was in hospital I think because so many people were just coming into our room and hurting him he was just really upset with everybody… and I'm becoming emotional. Yeah, so even when people came to our house he was really scared of everybody so that kind of ruined our everyday life because we couldn't have friends over 'cos he'd just scream constantly at people and I think because having the nurses at home as well he couldn't trust anybody coming to our house.[BCH F.W.]

Several participants described feelings of anxiety related to general uncertainties.

Night sweats, worrying about all sorts, just want, you know, really just completely in the dark about what was going to happen and I think because there's so much forewarning that it [infection] can happen, you know, then the worst does. It's a very upsetting thing to go through. [C&W 04]

For peer review only

Several participants talked about the importance of having a positive mental attitude throughout the process in order to cope with what was happening to them. Some remarked that this was something that they felt that they had to actively work to achieve.

> *I've tried to stay positive, stay mentally positive the fact that it could have been worse, it could have been a lot worse, yes its happened, I can't do anything about it… From day one I was trying to get a positive mental approach on it and on that second staff infection I have to admit I was starting to get down with it because I had tried and tried and tried to… I was on the sofa for four weeks without even really moving just to try and let the skin heal and seal and maximise its recovery. So that was tough but I still stayed positive all the way through but at the end, where I was… I think I went back on the 12th of September so June, July, I was probably two and a half months on those last couple of weeks was just- was an emotional struggle of just… just get to a stage where you can only stay positive so long (laughs) before I started to get a little bit down on the second staff infection, that it got to a stage where I was just thinking I've had enough of this now, I just want it to go away so I can get back on with day to day life.[NBT 012]*

> *With burns, what you don't realise is that as horrific and as awful as it is, if you've got that mentality you won't let anything get in your way.[C&W 02]*

It was clear that participants' mood states during recovery were not constant and many reported having periods where they felt very down.

> *But it is a long- you just don't… I have really down days where I think nothing's ever going to change and I get really frustrated. [NBT 013]*

In the longer-term, there was variability among participants with respect to how well they were coping. Many participants reported that they were coping well emotionally. The end of active treatment and/or a return to normal functioning was sometimes associated with a more positive mood state.

> *Yeah, when he knew we didn't have to come back anymore he was really happy and the sleeping got a bit better, so he used to really panic about coming in 'cos it would hurt. [BCH 018]*

> *.. once I got back to work and got back to the gym and all the day to day things, I'm totally fine with it now. Totally fine with it. [NBT 12]*

Some participants described feeling more cautious or fearful of situations that were similar to those in which the injury occurred.

> *and I would test it like eight times and he will be really- won't get near anything if it's hot. He is a bit more over-cautious with stuff. Very obsessive but- So that might be a long-term thing that comes from it. [BCH 18]*

Psychological impact on significant others (family and friends)

Many participants talked about the psychological impact that their injury had had on significant others. Family members often experienced distress at the time of the injury. Some parents expressed guilt that their child had been burned.

> *once we got to the medical tent we were just screaming so I just felt really guilty I must admit, that he'd burnt himself. And to be honest I should know better. Hot drinks. And I felt really bad 'cos I'm vegan so they had milk in a hot chocolate and I'd normally test but I didn't test it. [BCH 18]*

The psychological impact on other family members continued beyond the short-term.

For peer review only

sleep-wise as well, (female sibling) was waking up in the night, obviously she's 11 so she normally sleeps fine, and she was waking up in the night and having nightmares and putting lights on and (son, patient) didn't sleep so between them both I was up every hour pretty much for weeks and then obviously I had panic attacks… it all went a bit…[BCH 018]

Several participants also discussed how family or friends had been a source of support to help them cope with what has happened to them.

my mates do the usual reminders of sending me stupid videos of people having vapes exploding in their pocket and this catching on fire because that's what blokes do don't they really? … and the canoeing trip that we went on, they had a playlist of everything, Come on Baby Light My Fire, its just everything but that's what blokes do. But that's their way of making sure, I know it sounds stupid, making sure I'm emotionally alright because if I'd had turned around and told them to turn it off or punch them in the face they would realise that I'm not taking it well at the moment so…[NBT 012]

Return to normal functioning/ability to perform daily activities

The desire to return to their normal levels of functioning was a very common theme amongst participants. In the short-term period immediately after the injury, this was often about being able to perform basic tasks of self-care. Participants described difficulties feeding themselves, moving around, using the bathroom. These activities were difficult to carry out because participants were unable to move the affected area or suffered too much pain when doing so. Tiredness and feeling weak were also identified as limiting factors.

particularly bathroom and (inaudible), I absolutely hated the bedpans but I was too weak to get out of bed- [C&W 06]

The eating was very difficult and I had to be fed through a tube which I really didn't like.

Yeah, I really didn't like being fed through a tube but then eventually when they took it out I was so weak I couldn't actually feed myself. [C&W 06]

Some participants were also worrying at this stage about being able to perform activities in the future such as being able to return to work or education.

I was concerned with my hands because firstly I work with my hands. These, all these three fingers, the skin was right off down to the raw bone, seriously raw flesh on this where it had all come off. And obviously both palms, you can't really see it now because they've healed so well but I was concerned with my hands to start off with because that is my livelihood.[NBT 012]

For patients who were hospitalised following their injury, the ability to be able to perform basic care tasks was also linked to being able to be discharged from hospital and return home.

like cooking, shopping and, yeah, so all the practical things, like (hospital) tried to help me with that before I left and then I went to (burns unit) and I had to live in a flat on my own but that never worked out 'cos somebody was in the apartment so I never actually (inaudible). So if I'm honest I didn't feel fully prepared at the time but anyway, that was (inaudible) it's just the everyday practical stuff and washing, that was difficult, getting in and out of the bath [C&W 06]

In the weeks following the injury, many participants still found that their injuries limited their abilities to perform basic tasks. Several participants described having to make adaptations, take extra time or have help in order to function.

For peer review only

I had to wake up at five thirty because getting dressed was really difficult for me. Although it's not too far, obviously back then my arms and my hands couldn't move as well as it could today, they were still very rigid sometimes, they didn't move very well and if I wanted to unbutton my shirt, 'cos the dress code was smart, if I wanted to button up my shirt it took me a good ten, fifteen minutes to work on the buttons [C&W 02]

I made- I had like an extendable backscratcher and I Sellotaped- I got a bit of plaster tape off the nurses (laughs) and Sellotaped my fork to it so I could-[NBT 08]

Yeah, (partner) would usually cut everything up and then I would have my scoop and- [NBT 08]

In the medium to long-term, participants described how their injures impacted upon their ability to take part in recreation or leisure activities. Some participants talked about how limited movement of the affected area or fear of causing damage to their healing wound restricted their ability to play sports. Swimming was also commonly identified by participants as an activity that they were not confident to do. Concerns about chlorine stinging or the visibility of their scars when in a swimsuit were mentioned.

At the moment I've got a friend who does some swimming, some aqua aerobic and I used to do some aqua aerobic with her but I don't think I would now because at first I was worried about chlorine in the water, but then after it's not just for that, it's as well the look of it.[NBT 007]

A lot of participants talked about how their burn limited their ability to go out in the sun. This impacted on playing sports, swimming and also, for some participants, their holiday choices. Several participants mentioned having been advised to keep the area covered to protect it from sun damage, but for some participants they were also motivated to keep the area covered in hot weather due to concerns about appearance.

Just occasionally, you know, like yesterday, I took the grandchildren to the park, finding a seat that wasn't in the sun because I'm anxious- if I feel the sun on it I feel anxious because I've been told at least a year but it's going to be more than a year 'cos its obviously it is more than a year.[C&W 005]

You need to wear sunscreen wherever you go and now I have to wear double sunscreen not only for the burns 'cos it can burn but like 'cos of the discoid lupus and vitiligo could get worse. You can't go to beaches, you can't go out in the heat, your body can't tolerate certain things.[C&W 02]

Moving into the long-term, some participants talked about how they felt they coped much better psychologically once they were able to return to their normal activities. For others, the extent of their injury meant that they would never return to their pre-injury level of functioning and this was harder to accept.

so, you know, four, five months ago but over the last two or three months I've really been just back in normal life and probably the things I had decided are important have now disappeared into the (inaudible) and the normal functioning life and its less mulling over existential questions and just getting on with the day to day tasks. [C&W 04]

I think what troubles me now I've got carers and that means I've got to go and ask them and I've never been a person- [NBT 10]

Physiological effects of burn or treatment

Temperature – several participants reported feeling too hot in hospital in the short-term. Temperature problems were an issue in the longer term for a few patients. Two reported rapidly changing temperatures.

For peer review only

For one participant, the loss of sweat glands has caused long-term permanent issues with temperature control.

Thirst – feelings of thirst and dehydration were reported by several hospitalised participants.

Tiredness – tiredness an issue in the short-term but persisted beyond that for some interviewees.

Nutrition – Several participants mentioned that staff had impressed upon them the importance of eating to help with their short-term recovery. They also described issues they had with using feeding tubes or a lack of appetite. In the longer-term, one participant reported having lost a lot of weight and a second described speaking with a nutritionist to get advice regarding optimum nutrition for healing.

Length of hospital stay

Participants whose injuries necessitated a stay in hospital expressed differing views about the length of their stay. Some participants found it difficult and expressed the desire to go home.

> *For me I would- the thing that brought me down the most was having to be in the hospital- [NBT 08]*

> *Oh no, not that I wanted to get out of the hospital but it's not nice being- I mean they're very good, I mean I couldn't have been in a better place, they're absolutely amazing but you do don't you naturally want to go home? [NBT 014]*

One participant described how she refused to be admitted and insisted on receiving treatment (antibiotics) at home instead.

> *so they wanted to keep me in the hospital but I refused and I managed ok at home 'cos of antibiotics at home because I didn't want to stop in the hospital. But my first initial thing here I was upset because they wanted to keep me in hospital. [NBT 019]*

For others, the length of hospital stay felt just right or was experienced as a positive.

> *It didn't really bother me particularly because I had all the- I just slept and I was feeling so ill about it all and, um, and I just chatted to visitors and stuff like that but I think if I was in any longer I would have gone a bit stir crazy. I was getting to the point where I was like no I just need to go home now. [NBT 002]*

> *Like being in hospital I found, even though it's kind of a long time and everyone is kind of like I want to go home, I think being in hospital a long time is handy. I don't know if (inaudible) but I wouldn't have wanted to go home any earlier.[BCH F.W.]*

2. Interviews with professionals

Initial treatment, preventing infection, promoting healing and reduce scarring

Interviewed staff members mentioned a range of factors that are crucial around the time patients are admitted to hospital. Staff discussed reasons for importance of these factors which can jointly be seen as a chain reaction of events where each is an important influencing factor for the next.

To begin with, staff involved in the treatment of smaller burns were mostly worried about adequate management of the injury and caring for the wound during the very acute phase. This included a quick and smooth process of assessment, cleaning, scrubbing and dressing.

For peer review only

> "[…] they need initial scrubbing, whether that be in theatre or in the ward, and getting them dressed up so that's the initial phase I would say is only to do acutely without thinking anything else." [BCH012]

Further prompting revealed that the underlying reason is the prevention of infection. Infections were mentioned by the majority of staff as the most important concern at the point of admission to hospital.

> "For infection, just to make sure there's no infection. If there's a lot of dead skin hanging around or blisters aren't de-roofed you could potentially get an infection just in the fluid that's still around or the dead skin." [BCH001]

Staff often mentioned that the prevention is important to avoid prolonging the healing process. Healing was seen as an important outcome, especially in connection with smaller burns.

> "[…]or that they do get a small infection, you know, the process is delayed then isn't it and the healing time is prolonged" [MH001]

> "[…] quite often we're very focused on dealing with the physical aspects of the burn injury, so making sure the burn wound heals, preventing infection. […] we're very focused on managing that wound and […] making sure the wound heals […]. [BCH014]

> "Infection is what we keep an eye out for and that the right dressings are going on to maximise healing." [BCH013]

> "[…] having them being cleaned and assessed and then you kind of do think about the healing as well because they're smaller, they'll heal within two weeks and then you kind of do think more about the healing […]" [BCH012]

Whilst scarring was not important for staff at the acute stage of the injury, it was often mentioned by staff as one of patients' major concerns. Participant BCH014 described the whole chain of events nicely:

> "You know, or if you're going along that journey of the wound healing, you know, sometimes you can come into problems like infection, for example. If there's anything delaying healing, then scarring can obviously still happen. […] And that's something that is important to us is dressing the wound in a way that will kind of protect the wound [...] [a]nd help it heal quickly." [BCH014]

Pain and distress management

Almost all staff considered pain management a vital part during the acute phase of injury treatment. For smaller burns a similar chain reaction of events was observed, where the underlying reason to control pain was to reduce the distress that patients and their carers were subject to.

> "Make things worse in time... in terms of like dealing with their pain management, can be quite distressing for the family. So I think feelings of, yeah, distress for the parent and in terms of managing that child's pain is probably the most paramount when they first see us.

> […] I've... remember once I had one parent who did panic when I was cleaning the wound, and wasn't quite expecting for me to be removing skin. So she felt as though I was doing something that I perhaps shouldn't..." [BCH014]

Further prompting elicited that reduced psychological trauma was a key motivation to manage the distress.

For peer review only

"Because being burnt is painful and we scrub the wound and take the blisters off which is very painful. And the children need to come back for repeat dressing changes so we need to make the first one as good as it can be. It's never nice but we need to make it as well managed and painless as possible." [BCH013]

Being in control

A small number of staff mentioned that feeling in control of the procedural steps and the situation is most important to them to ensure a high standard of burns treatment delivery. Participants mentioned the importance of underlying factors such as being well prepared for the treatment or presence of a well-functioning team.

"It's being in control of that procedure I feel as a clinical professional, making sure that, yeah, that if you lose- I think once you've lost the control of the procedure in some way then you've lost the parent confidence in you." *[BCH006]*

Other consequences of a potential lack of control were acknowledged by two staff members. These included personal impacts on self-confidence, patients' confidence in the treatment and potential further causes of stress. In this sense, this theme may be linked to previous findings where interviewees mentioned that a well-managed procedure also contributes to reducing distress (see also quote from BCH013 above).

"You don't want them to leave distressed and overwhelmed by it all. And then in the future you know that they're not going to mind so much coming back for future dressings so it just helps" [BCH001]

Survival

Interestingly, only two members of staff (BCH007, NBT006) mentioned survival as the most important outcome and BCH007 noted "*the most important outcome is survival because there are those patients that are very poorly and you just want them to survive*". This is only applicable for severe and larger body surface burns.

Facilitate independence from healthcare

Following discharge, it was important to the majority of staff to help patients gain independence from the care of the hospital. Several sub-themes emerged that could contribute to facilitating independence.

Self-management of wound

A major concern for staff was that patients are able to manage their wound when returning home. This included continued appropriate wound care to prevent further scarring and getting the wound to "a normal state" [MH002].

"I would say our main concern is will the families be able to continue with the treatment that we've taught them. So, it might be creaming and massage […] and protecting it from the sun." [BCH30102019]

"[…] making sure that we instruct them […] on the massage and moisturisation at home, which they should be doing several times a day to kind of help." [BCH014]

This included the self-efficacy and confidence to carry out the wound treatment and providing the tools and information to carry it out in the right way (e.g. how to do the dressings, cleaning etc).

I guess that they feel equipped and empowered to be the main carer for their child [BCH006]

to take ownership of their own wounds from the outset so that they can manage their own lives 'cos of course they've come here, you have these rather complicated looking dressings put on and everything looks kind of amazingly difficult and complicated whereas commonly it's really straightforward and a lot of what we do isn't necessarily super sterile, its clean and these are things often times that can be managed very well at home with a bit of support from family [MH001]

Patient knowledge and understanding

At the point of discharge, it was considered critical that patients are given the advice needed so they would be *"well equipped with the information that they need" [BCH006]* to help patient understanding of the wound and treatment. This encompassed process-related information related to the self-management of the wound as described above.

> *"It's explaining to them what they need to be doing to achieve this wound to heal, and like I say before, being honest about it" [NBT026]*

> *I think that's quite important to pass that on and advice about sun protection is really important because it's new skin so that they need to be advised about that as well. [BCH008]*

Staff repeatedly mentioned it was necessary to ensure that patients understand signs of infection and closely monitor the wound for e.g. redness, swelling or changes of the wound and starting to feel unwell.

> *"It's just knowing that the patient understands how to look after their wounds, and to look out for signs of infection […] if they start [t]o feel unwell and it's their understanding of that really" [NBT026].*

It was vital for staff to emphasise that patients' understanding includes the management of their expectations with regards to the outcome of their injury.

Knowledge of availability of support

After discharge, staff frequently mentioned it was important to them to communicate the constant support available to patients and when to make use of it.

> *"My biggest goal and concern for them is can they access everything they want to access." [BCH007]*

> *"Identify when they need help and be able to just call us and we would say come back straight away if there's a problem" [BCH006]*

Independence from healthcare was less of a priority as time progressed, however, availability of psychological support was mentioned as important in the long-term and is further described in the 'Psychology' theme.

Discomfort during healing

Concerns about contractures and the desire to improve any tightness of the skin were common concerns for larger burns.

> *"So giving them a contractual release to allow them to return to as normal a state as possible, even if it's not specifically function in terms of what they do but how they can stand, how they can hold their heads" [NBT001]*

A minority of staff were concerned about patients' pain control after discharge or experiencing itching.

> *"When they heal can get quite itchy, so by putting the moisturiser on, keeping it... you know, preventing it from drying, scabbing, you are going to help that itchiness." [BCH014]*

For peer review only

"We want them to go home pain-free, or very near pain-free, where they're able to control their pain at home". [NBT026]

Other interviewees had different opinions and focused solely on self-management of the wound:

"I mean that's the only real priority there is then because they shouldn't be in any pain, they're not going to need any further follow-up so it's just making sure that they manage the healed wound" [BCH006]

Psychology

A large number of interviewees mentioned the importance of potential psychological impacts in the long-term.

"I mean long-term the important outcomes really are how they are psychologically" [BCH30102019].

"I suppose what I'm saying is the psychology, […] can be long-term, so we would be perhaps seeing families where could be a long time previous that the injury… when their children were toddlers and then they perhaps go into puberty and if they've had chest scarring or whatever or where teenagers are wanting to wear strappy tops or bikinis or proms, that kind of thing, it all floods back and that's when could be years ago that they had the actual burn but we would then perhaps get involved." [BCHRP30102019]

"I think their general wellbeing and happiness is also a long-term goal." [BCH007]

"In some, you know, the injuries that had really knocked someone for six and kind of really turned their lives upside down in the process." [NBT0004]

Psychological wellbeing and impact of injury & scarring

In particular, concerns were raised most frequently in connection with how the injury happened or the appearance of the scar (cosmesis). Probing revealed several aspects affecting the psychological wellbeing. Specifically, underlying internal (e.g. self-esteem, acceptance and normalisation of the injury) and external influences (e.g. bullying, worry related to intimate relationships and other people's views) were mentioned by staff.

"I would say cosmesis is the most one 'cos burns can be disfiguring, they can leave scars which are not very nice and not very slightly, so it affects patients psychologically and in kind of their social groups as well and also the parents as well." [BCH012].

"I'd be still cautious about psychology and how they've coped, especially if it is going to scar, what impact that's going to have on their self-esteem and bullying potentially at the school." [BCH013]

"Some people cope with it, some people don't. Everybody's different, and just giving them coping mechanisms for the community really." [NBT026]

"Definitely anxiety… yeah. Just the whole impact of the injury, I think it's just frightens them in many ways." [BCH008]

In particular, staff thought it was important that psychological support was available in the long-term which recognised signs of PTSD (e.g. sleeping problems, flashbacks and impacts on relationships).

[…] that psychologically they're supported."

For peer review only

"I suppose it's some sort of way of keeping an eye on people without being intrusive so that if stuff emerges down the line […] It's about, A, if they're still getting post traumatic symptoms and, B, the impact it's having on them and if, for example, […] they're frightened to go to sleep or it's affecting their relationship or whatever then you'd start to pay more attention." [NBT022]

Impact on families and daily activities

Professionals often mentioned the psychological impact of the burn on daily activities.

"[…] even if they haven't got scarring but then just integrating back into going to school, that might be hard so that could impact their lives that way." [BCH30102019]

When the injury involved children, staff highlighted the added psychological impact on parents and the family as a whole.

"So I think there's a huge psychological aspect on all the patients and actually the mobility and the functional side is only the more severe side of a patient 'cos obviously the less injured patients will get better very quickly from the function and mobility point of view and it's never a problem. So I think there's, yeah, psychological with all of them and mobility and function with most of them I'd say." [BCH007]

"it depends how the injury happened, if there's any like psychological impact on either the child... I mean, quite often the children we see are under the age of five and they don't often remember what happened. But there can be quite big like load of guilt on either the parent... if it involves the parent or quite often involves grandparents who have put, you know, a cup of coffee on the side and the child has grabbed it and sort of tipped it down themselves, if it's created... if it caused a large burn and a large injury, and they had quite a big treatment period in hospital, then that person, the grandparent or the parent, can have a lot of guilt and a lot of distress around that situation. So quite often it's, I guess, dealing with that." [BCH014]

Functional impacts

Impacts of the injury (or scar) on how it affects patients returning to normal function was an important concern for staff after discharge and in the long-run.

"Well to be living as normal a functioning, happy life as possible after having a burn injury." [BCH006]

"Function essentially, yeah […] Returning to their pre-burn level of function." [MH003]

Return to normal activities

One of the most important outcomes for staff during the medium-term (for smaller burns) and in the long-term (for larger burns) was that patients can return to their normal activities. This predominantly included the ability to return to work and school, and carry out day-to-day tasks and hobbies or other leisure activities.

"It's just encourage them just to get back into normal life now, just carry on things as normal." [BCH008]

"The most important outcome is, for me personally and professionally, it's their physical outcome. So it's are they walking, are they active, are they engaging in every activity that they would want to engage in." [BCH007]

"So initially I think it's kind of their return to their sort of family roles and their normal activities, when can I go back to work, when can I do my leisure activities." [MH002]

For peer review only

Movement restrictions and contractures

Staff directly involved in the treatment of burns affecting relevant areas (OT, Physios) were worried about the impact of the wound on movement.

> *"[…] hoping that the burn isn't affecting them anymore and that it is all settled down and, yeah, just living a normal life. [BCH008]*

> *"[…] the focus is very strongly on functionality. In other words my scar is preventing me from doing this, can you sort my scar out." [NBT001]*

> *"I would say mainly functional is the later one. […] I think the ultimate aim is them to be not functionally restricted with the scars and the scar that is down and not very angry and almost back to normal skin. " [BCH012]*

> *"Long-term would be the scarring and whether contracture is affecting movement" [BCH013]*

Specific location of burns were frequently mentioned as requiring additional attention because they affect mobility.

> *just being mindful [...] that [...] if the burn injury went over a certain area, so if it goes over a joint, sometimes we might book […] an appointment with the OTs anyway, like three months down the line.[BCH014]*

> *"And then… especially around joints, so they lose their mobility of their joint as well, and it may cause… maybe, you know, they've been quite into their sport, and that may impact, so they… they can't any longer play the sport that they were doing before, or maybe the job…" [NBT026]*

Scarring and continued scar management

A commonly mentioned important long-term concern for staff was the level of scarring and associated treatments to manage it.

> *"I think scarring probably is like the biggest." [BCH014]*

Worry about scarring was twofold: staff were concerned about the functional impacts in the medium to long-term as well as the appearance of the scar.

> *"I think the ultimate aim is them to be not functionally restricted with the scars and the scar that is down and not very angry and almost back to normal skin" [BCH012]*

Concerns about the appearance were determined by patients' perception and worry about scarring, linked to potential psychological impacts.

> *"From a clinician's point of view it's a great outcome, her scar wasn't lumpy or bumpy, there's not really much there that we can see but from their point of view it affects them and how they cope, especially in teenage years as well." [BCH012]*

For larger burns, getting the wounds to "their optimum" [BCH30102019] is a priority for staff involved in the ongoing longer-term management of scars. This includes the appropriate treatment under the scar management path, e.g. pressure garments or surgery to "just get[…] the best outcome from that injury that you can." [BCH30102019]

> *"scarring, needing scar management help, perhaps like pressure garments or steroid creams" [BCH001]*

For peer review only

> *"So in terms of therapies by scar management teams and maybe pressure garments, silicones, that sort of thing, and then ultimately potentially like steroid injections or some form of surgery […]." [BCHRP30102019]*

Psychological effects of the continued and potentially years-long treatment were also acknowledged by staff.

> *"So, not only do they have to get over that initial situation, they have to live with their… their scars and ongoing surgery, and things like that. So some people it's lifelong." [NBT026]*

> *"The size of the injury is important to a certain extent and the amount of scarring someone's going to have and the potential reconstructive surgery because of course we're potentially re-traumatising people or people are having new things that they've got to kind of cope with." [MH002]*

> *"Some children have a really tough journey in that way, for years, who are under scar management, under therapy for repeated […] reconstructive surgery [BCH006].*

3. Combining themes

Information from the descriptive report were compared and contrasted and eventually condensed into combined themes. During this process, decisions were made to combine, separate or discard themes and suitable labels were agreed in team discussions. Tables S4 and S5 list the identified themes, how these are linked to combined themes and which outcome domains could be mapped to themes' conceptual content.

Table S1: Patient interviews themes, combined themes, and mapped outcome domain

**Themes (sub-themes)**	**Combined themes**	**Outcome domains (outcome category) as per outcome classification by Young et al (2019)**
Scarring	Scarring	• Scar texture (scar-related)<br>• Scar colour (scar-related)<br>• Scar size (scar-related)<br>• Appearance (patient-reported)
Pain	Pain and discomfort	• Burn wound pain (patient-reported)<br>• Pain during procedures (patient-reported)<br>• Itch (patient-reported)<br>• Scar pain (patient-reported)<br>• Effect of scar on movement [contractures] (patient-reported)<br>• Comfort of dressings (patient-reported)
Tightness		
Itching		
Infection	Infection	• Burn wound infection (complications)<br>• Sepsis (complications)
Appearance of the wound	Healing	• Burn wound healing (pathophysiological)<br>• Appearance (patient-reported)
Time to healing		
Psychological effects (Psychological impact on significant others [family and friends])	Psychological wellbeing	• Psychological well-being (patient-reported)<br>• Generalised anxiety (patient-reported)<br>• Mental ability (patient-reported)<br>• Quality and quantity of sleep (patient-reported)

For peer review only

Return to normal functioning/ability to perform daily activities	Function	• Ability to carry out daily tasks (patient-reported)<br>• Return to work/school or previous function (patient-reported)<br>• Appearance (patient-reported)
Uncertainty, lack of knowledge and the need for information	Uncertainty	None identified
Length of hospital stay	Discarded	
Physiological effects of burn or treatment	Discarded	

Table S2: Professional interviews themes, combined themes, and mapped outcome domain

**Themes (sub-themes)**	**Combined themes**	**Outcome domains (outcome category) as per outcome taxonomy**
Initial treatment, preventing infection, promoting healing and reduce scarring	Healing	• Burn wound healing (pathophysiological)
	Infection	• Burn wound infection (complications)
Pain and distress management	Pain and discomfort	• Use of medicines to treat symptoms (healthcare-related)<br>• Burn wound pain (patient-reported)<br>• Effect of scar on movement [contractures] (patient-reported)
Discomfort during healing		
Scarring and continued scar management	Scarring	• Treatment for scars (scar-related)<br>• Scar texture (scar-related)<br>• Scar colour (scar-related)<br>• Scar size (scar-related)<br>• Appearance (patient-reported)
Functional impacts (Return to normal, Movement restrictions)	Function	• Mobility (pathophysiological)<br>• Ability to carry out daily tasks (patient-reported)<br>• Return to work/school or previous function (patient-reported)
Psychology (Psychological wellbeing and impact of injury & scarring, Impact on families and daily activities)	Psychological wellbeing	• Psychological well-being (patient-reported)<br>• Generalised anxiety (patient-reported)<br>• Mental ability (patient-reported)<br>• Quality and quantity of sleep (patient-reported)
Survival	Survival	• Death from burn injury (complications)<br>• Death from any cause (complications)
Being in control	Sense of control	None identified
Facilitate independence from healthcare (Self-management of wound/ independence, Patient knowledge and understanding, Knowledge of availability of support)	Patient knowledge, understanding & support	None identified

For peer review only

Standards for Reporting Qualitative Research (SRQR)*

http://www.equator-network.org/reporting-guidelines/srqr/

Title and abstract

	Page/line no(s).
**Title** - Concise description of the nature and topic of the study Identifying the study as qualitative or indicating the approach (e.g., ethnography, grounded theory) or data collection methods (e.g., interview, focus group) is recommended	1
**Abstract** - Summary of key elements of the study using the abstract format of the intended publication; typically includes background, purpose, methods, results, and conclusions	2

Introduction

**Problem formulation** - Description and significance of the problem/phenomenon studied; review of relevant theory and empirical work; problem statement	4
**Purpose or research question** - Purpose of the study and specific objectives or questions	4

Methods

**Qualitative approach and research paradigm** - Qualitative approach (e.g., ethnography, grounded theory, case study, phenomenology, narrative research) and guiding theory if appropriate; identifying the research paradigm (e.g., postpositivist, constructivist/ interpretivist) is also recommended; rationale**	5
**Researcher characteristics and reflexivity** - Researchers' characteristics that may influence the research, including personal attributes, qualifications/experience, relationship with participants, assumptions, and/or presuppositions; potential or actual interaction between researchers' characteristics and the research questions, approach, methods, results, and/or transferability	5-6, 16
**Context** - Setting/site and salient contextual factors; rationale**	2, 5
**Sampling strategy** - How and why research participants, documents, or events were selected; criteria for deciding when no further sampling was necessary (e.g., sampling saturation); rationale**	5
**Ethical issues pertaining to human subjects** - Documentation of approval by an appropriate ethics review board and participant consent, or explanation for lack thereof; other confidentiality and data security issues	5, 20-21
**Data collection methods** - Types of data collected; details of data collection procedures including (as appropriate) start and stop dates of data collection and analysis, iterative process, triangulation of sources/methods, and modification of procedures in response to evolving study findings; rationale**	6

For peer review only - http://bmjopen.bmj.com/site/about/guidelines.xhtml

**Data collection instruments and technologies** - Description of instruments (e.g., interview guides, questionnaires) and devices (e.g., audio recorders) used for data collection; if/how the instrument(s) changed over the course of the study	6
**Units of study** - Number and relevant characteristics of participants, documents, or events included in the study; level of participation (could be reported in results)	7-8
**Data processing** - Methods for processing data prior to and during analysis, including transcription, data entry, data management and security, verification of data integrity, data coding, and anonymization/de-identification of excerpts	6-7
**Data analysis** - Process by which inferences, themes, etc., were identified and developed, including the researchers involved in data analysis; usually references a specific paradigm or approach; rationale**	6-7
**Techniques to enhance trustworthiness** - Techniques to enhance trustworthiness and credibility of data analysis (e.g., member checking, audit trail, triangulation); rationale**	5-7

Results/findings

**Synthesis and interpretation** - Main findings (e.g., interpretations, inferences, and themes); might include development of a theory or model, or integration with prior research or theory	9
**Links to empirical data** - Evidence (e.g., quotes, field notes, text excerpts, photographs) to substantiate analytic findings	9-14

Discussion

**Integration with prior work, implications, transferability, and contribution(s) to the field -** Short summary of main findings; explanation of how findings and conclusions connect to, support, elaborate on, or challenge conclusions of earlier scholarship; discussion of scope of application/generalizability; identification of unique contribution(s) to scholarship in a discipline or field	15-16
**Limitations** - Trustworthiness and limitations of findings	16

Other

**Conflicts of interest** - Potential sources of influence or perceived influence on study conduct and conclusions; how these were managed	20
**Funding** - Sources of funding and other support; role of funders in data collection, interpretation, and reporting	20

*The authors created the SRQR by searching the literature to identify guidelines, reporting standards, and critical appraisal criteria for qualitative research; reviewing the reference lists of retrieved sources; and contacting experts to gain feedback. The SRQR aims to improve the transparency of all aspects of qualitative research by providing clear standards for reporting qualitative research.

For peer review only

For peer review only - http://bmjopen.bmj.com/site/about/guidelines.xhtml

**The rationale should briefly discuss the justification for choosing that theory, approach, method, or technique rather than other options available, the assumptions and limitations implicit in those choices, and how those choices influence study conclusions and transferability. As appropriate, the rationale for several items might be discussed together.

Reference:

O'Brien BC, Harris IB, Beckman TJ, Reed DA, Cook DA. **Standards for reporting qualitative research: a synthesis of recommendations.** *Academic Medicine*, Vol. 89, No. 9 / Sept 2014 DOI: 10.1097/ACM.0000000000000388

For peer review only

For peer review only - http://bmjopen.bmj.com/site/about/guidelines.xhtml
